# Genomic Epidemiology of Gonococcal Resistance to Extended-Spectrum Cephalosporins, Macrolides, and Fluoroquinolones in the United States, 2000–2013

**DOI:** 10.1093/infdis/jiw420

**Published:** 2016-09-16

**Authors:** Yonatan H. Grad, Simon R. Harris, Robert D. Kirkcaldy, Anna G. Green, Debora S. Marks, Stephen D. Bentley, David Trees, Marc Lipsitch

**Affiliations:** 1 Department of Immunology and Infectious Diseases; 2 Center for Communicable Disease Dynamics, Department of Epidemiology, Harvard T. H. Chan School of Public Health; 3 Division of Infectious Diseases, Brigham and Women's Hospital and Harvard Medical School; 4 Department of Systems Biology, Harvard Medical School, Boston, Massachusetts; 5 Centers for Disease Control and Prevention, Atlanta, Georgia; 6 Wellcome Trust Sanger Institute, Hinxton; 7 Department of Medicine, University of Cambridge and Addenbrookes Hospital, Cambridge, United Kingdom

**Keywords:** *Neisseria gonorrhoeae*, gonorrhea, antibiotic resistance, genomic epidemiology, molecular diagnostics, cephalosporins, macrolides, fluoroquinolones

## Abstract

***Background.*** Treatment of *Neisseria gonorrhoeae* infection is empirical and based on population-wide susceptibilities. Increasing antimicrobial resistance underscores the potential importance of rapid diagnostic tests, including sequence-based tests, to guide therapy. However, the usefulness of sequence-based diagnostic tests depends on the prevalence and dynamics of the resistance mechanisms.

***Methods.*** We define the prevalence and dynamics of resistance markers to extended-spectrum cephalosporins, macrolides, and fluoroquinolones in 1102 resistant and susceptible clinical *N. gonorrhoeae* isolates collected from 2000 to 2013 via the Centers for Disease Control and Prevention's Gonococcal Isolate Surveillance Project.

***Results.*** Reduced extended-spectrum cephalosporin susceptibility is predominantly clonal and associated with the mosaic *penA* XXXIV allele and derivatives (sensitivity 98% for cefixime and 91% for ceftriaxone), but alternative resistance mechanisms have sporadically emerged. Reduced azithromycin susceptibility has arisen through multiple mechanisms and shows limited clonal spread; the basis for resistance in 36% of isolates with reduced azithromycin susceptibility is unclear. Quinolone-resistant *N. gonorrhoeae* has arisen multiple times, with extensive clonal spread.

***Conclusions.*** Quinolone-resistant *N. gonorrhoeae* and reduced cefixime susceptibility appear amenable to development of sequence-based diagnostic tests, whereas the undefined mechanisms of resistance to ceftriaxone and azithromycin underscore the importance of phenotypic surveillance. The identification of multidrug-resistant isolates highlights the need for additional measures to respond to the threat of untreatable gonorrhea.

As efforts to develop rapid diagnostic tests that rely on genetic markers of resistance emerge, it is critical to identify the prevalent resistance mechanisms and the extent to which they are emerging through clonal expansion, recombination, or de novo mutation. While a cross-sectional analysis of the mechanisms of resistance in a population of clinically isolated pathogens can provide a snapshot, a large-scale longitudinal analysis reveals temporal trends in resistance mechanisms, low prevalence mechanisms, and the epidemiology of resistance acquisition and spread. These in turn can guide research into the genes underlying resistance and development of molecular diagnostic tests and novel therapeutics.

Previously, we used a population-based genome sequencing analysis to investigate the genetic basis of reduced susceptibility to the oral extended-spectrum cephalosporin (ESC) cefixime in *Neisseria gonorrhoeae* [1]. That initial study covered a narrow time window (2009–2010) and a single antimicrobial agent. Here, we extend our analysis to include 1102 gonococcal isolates drawn from across the United States and over 14 years (2000–2013) and resistance to 3 of the most clinically relevant antimicrobial classes: the ESCs, including cefixime and ceftriaxone; macrolides, specifically azithromycin; and fluoroquinolones, specifically ciprofloxacin. We focus on these 3 classes because the current recommendation for treatment of gonorrhea is dual therapy with ceftriaxone and azithromycin [2] and because advances in development of molecular diagnostic tests may prompt reconsideration of treatment with fluoroquinolones [3, 4], which has not been recommended since the population prevalence of quinolone resistance exceeded 5% [5].

For each of the antimicrobials—the ESCs, azithromycin, and ciprofloxacin—several questions pertain: how much do known resistance mutations explain observed phenotypic resistance? To what extent does resistance appear de novo versus spread through clonal expansion of a resistant strain? How many times has resistance appeared? These questions have implications for the study of the dynamics of resistance, for the development of public health surveillance and intervention strategies, and for the development and use of molecular diagnostic tests that rely on genotype to predict resistance.

## METHODS

### Specimen Collection and Phenotypic Antimicrobial Susceptibility Testing

We obtained isolates of *N. gonorrhoeae* from the Centers for Disease Control and Prevention's Gonococcal Isolate Surveillance Project (GISP), with samples collected as described [6]. Minimum inhibitory concentrations (MICs) were determined by agar dilution susceptibility testing, with some measurements confirmed by the Etest. Antimicrobial susceptibility was interpreted according to Clinical and Laboratory Standards Institute for ciprofloxacin [7], and according to Centers for Disease and Control and Prevention's guidelines for cefixime, ceftriaxone, and azithromycin, for which Clinical and Laboratory Standards Institute resistance criteria have not been established [8]. See the Supplementary Methods for further details.

### DNA Sequencing and Analysis

DNA was prepared from single colonies and sequenced with the Illumina HiSeq platform according to standard protocols [1]. Illumina reads were mapped to a reference strain, FA1090 (GenBank accession number AE004969) using BWA MEM [9]. Single-nucleotide polymorphisms (SNPs) were called (ie, filtered to determine the working set of SNPs from all candidate sites) according to previous parameters [1]. Each Illumina read set was assembled with velvet (version 1.0.12) [10] and VelvetOptimiser (http://bioinformatics.net.au/software.velvetoptimiser.shtml). See the Supplementary Methods for further details of analysis of NG-MAST, recombination, and population structure.

## RESULTS

### Population Structure

The 1102 isolates include 270 with reduced ESC susceptibility (ESC^RS^), 294 Azi^RS^, and 594 quinolone-resistant *N. gonorrhoeae* (QRNG); these totals include multidrug-resistant isolates. The collection spans 2000–2013 and 36 sexually transmitted diseases (STD) clinics from across the United States (Supplementary Methods, Supplementary Table 1, and Supplementary Figure 1).

**Figure 1.**
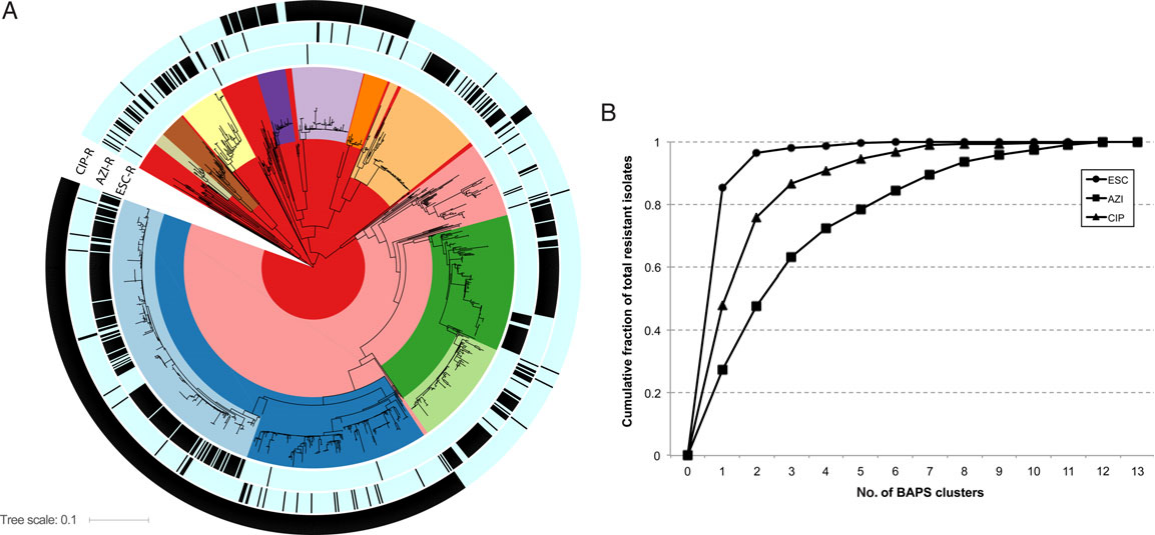
*A*, Maximum likelihood whole-genome-sequence phylogeny of 1102 *Neisseria gonorrhoeae* isolates, based on single nucleotide polymorphisms from mapping to the FA1090 reference genome. The coloring in the clades reflects the groups predicted from Bayesian analysis of population structure (BAPS). The black hashes in the 3 outer rings reflect (from inner to outer rings) reduced susceptibility to the extended-spectrum cephalosporins (ESCs), reduced susceptibility to azithromycin (AZI), and ciprofloxacin (CIP) resistance. *B*, Plot of cumulative fraction of isolates for the ESCs, AZI, and CIP by number of BAPS groups.

In the phylogeny, many of the isolates group into clades, reflected by Bayesian analysis of population structure (BAPS) clusters and corresponding to antimicrobial susceptibility patterns (Figure 1*A*). This clade structure likely reflects the expansion of resistant lineages. Plotting the cumulative fraction of total resistant isolates by the number of BAPS clusters reveals that ESC and ciprofloxacin resistances are primarily attributable to the expansion of a small number of BAPS groups, whereas azithromycin resistance is more distributed throughout the phylogeny (Figure 1*B*). Numerous isolates on long terminal branches suggest existence of substantial unsampled diversity.

### Reduced Susceptibility to the ESCs

**Figure 2.**
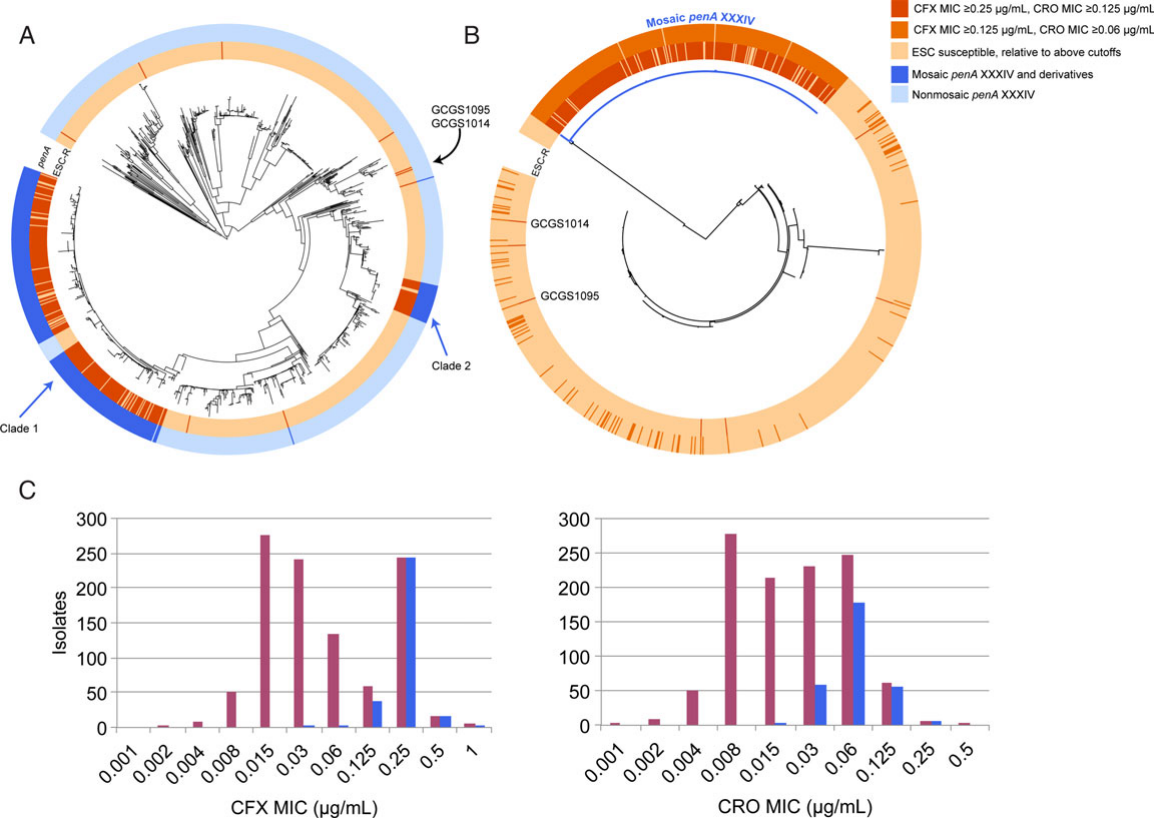
*A*, Maximum likelihood whole-genome-sequence phylogeny of 1102 *Neisseria gonorrhoeae* isolates, based on single-nucleotide polymorphisms from mapping to the FA1090 reference genome, with the inner annotation ring representing those isolates with reduced extended-spectrum cephalosporin (ESC) susceptibility and the outer ring representing which isolates have a mosaic *penA* XXXIV or derivative allele. Clades 1 and 2 are identified. A lineage within clade 1 lacks the mosaic *penA* XXXIV and is susceptible; these isolates appear to have undergone a recombination that replaced the mosaic *penA* XXXIV allele with mosaic *penA* XXXVIII allele. *B*, Maximum likelihood phylogeny of the *penA* locus, extracted from the de novo–assembled genomes for each of the isolates. The branch in blue indicates isolates with the mosaic *penA* XXXIV and derivative alleles. The coloring along the inner and outer annotation rings indicate isolates by the minimum inhibitory concentration (MIC) threshold defined in the key. *C*, Histograms indicating the number of isolates by cefixime (CFX) and ceftriaxone (CRO) MICs. The histogram in purple indicates the total number of isolates—those with and without a mosaic *penA* XXXIV-like allele—per MIC, and the histogram in blue indicates the number of isolates with a mosaic *penA* XXXIV–like allele.

The whole-genome phylogeny reveals that the majority of ESC^RS^ is due to expansion of 2 clades possessing the mosaic *penA* XXXIV allele (Figures 1 and 2), previously seen in our analysis of isolates from 2009 to 2010 [1] and confirming that this is the predominant *penA* allele associated with resistance in the sampled time frame (2005–2013). We additionally identify sporadic isolates with ESC^RS^ that do not possess the mosaic *penA* XXXIV allele (Figure 2 and Supplementary Table 1). Parsimony reconstruction infers 13 independent acquisitions (range, 10–16 acquisitions) and 35 losses (range, 32–35 loss) leading to the 270 ESC^RS^ isolates in this data set.

**Figure 3.**
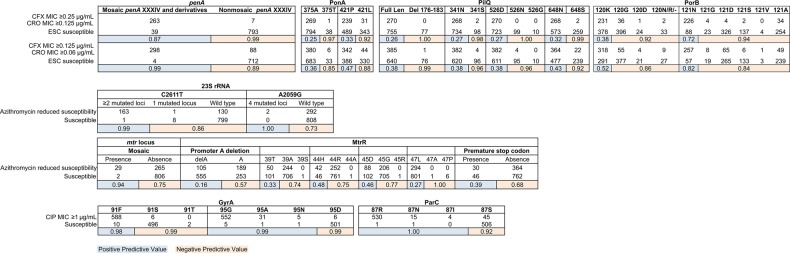
Positive (blue) and negative (tan) predictive values for resistance to the ESCs, azithromycin, and ciprofloxacin as determined in the dataset of 1102 gonococcal genomes. Two MIC thresholds are presented for the ESCs, representing the current threshold for reduced susceptibility and one dilution lower. Azithromycin reduced susceptibility is defined by MIC ≥ 1µg/mL for isolates between 2000-2004 and ≥ 2µg/mL starting in 2005, due to a change in the media used for agar dilution testing (see Supplemental Methods). Abbreviations: CFX, cefixime; CRO, ceftriaxone; ESC, extended-spectrum cephalosporin; MIC, minimum inhibitory concentration; rRNA, ribosomal RNA.

ESC^RS^ is primarily explained by the presence of the mosaic *penA* XXXIV and derivative alleles (Figures 2 and 3), with 98% of isolates with reduced susceptibility to cefixime (MIC ≥0.25 µg/mL) and 91% of isolates with reduced susceptibility to ceftriaxone (MIC ≥0.125 µg/mL) possessing these alleles (Supplementary Table 1). A less stringent MIC cutoff suggests that other pathways may contribute to low-level reduced cephalosporin susceptibility, as the percentage of isolates possessing the mosaic *penA* XXXIV and derivative alleles at a cefixime MIC of ≥0.125 µg/mL and a ceftriaxone MIC of ≥0.06 µg/mL decreases to 91% and 76%, respectively (Supplementary Table 1).

The presence of a mosaic *penA* XXXIV allele does not confer resistance equally to cefixime and ceftriaxone (Figure 2*C*). While only 1% of isolates with mosaic *penA* XXXIV alleles have a cefixime MIC of ≤0.125 µg/mL, 21% of mosaic *penA* XXXIV allele–containing isolates have a ceftriaxone MIC of ≤0.03 µg/mL.

Several of the ESC^RS^ isolates, including 2 with cefixime MICs among the highest observed in this data set (GCGS1095 and GCGS1014, with MICs of 1 and 0.5 µg/mL, respectively), share identical *penA* alleles to those in susceptible isolates (Figure 2*B*), indicating the involvement of other loci in some ESC^RS^ phenotypes.

Several other loci have been previously associated with β-lactam resistance, including *pilQ*, *ponA*, *porB*, and plasmid-borne TEM β-lactamases [11]. *pilQ* mutations can confer ESC resistance in vitro [12], but *pilQ* mutations have not been associated with ESC resistance in clinical isolates [13]. We observe several *pilQ* and *ponA* alleles that have a high negative predictive value for resistance (Figure 3). Mutations at amino acid positions 120 and 121 in the porin-encoding *porB* have been associated with β-lactam resistance [14], but neither the 120K (Figure 3) nor the 120D/121D genotypes are associated with reduced ESC susceptibility in this data set (only 2 isolates have the 120D/121D genotypes, and both are ESC susceptible). The 7 ESC^RS^ isolates lacking a mosaic *penA* do not share a *porB* allele or amino acids at positions 120 and 121 (Supplementary Figure 2), and no *porB* sites are exclusively shared by these 7 isolates. A TEM β-lactamase is present in 35 isolates, but only 3 of these are ESC^RS^ (Supplementary Table 1).

Previously, a model based on samples from 2009 to 2010 that ESC^RS^ circulated primarily among men who have sex with men (MSM), on the West Coast, with an uncertain number of entries into the United States [1]. With this larger data set, we observe that the earliest cluster of mosaic *penA* XXXIV containing ESC^RS^ appeared in 2005 in 2 isolates (GCGS0920 and GCGS0944) from men who have sex with women (MSW), in Miami and Portland (Supplementary Table 1 and Supplementary Figure 3), and subsequently circulated in MSW. The bicoastal appearance and spread of this sublineage of clade 1 among MSW is in contrast with circulation of clade 1 after 2009 predominantly in MSM (n = 146 [69%]) and in the western United States (n = 147 [69%], comprising clinic sites Honolulu, Las Vegas, Los Angeles/Orange County, Phoenix, Portland, San Diego, San Francisco, and Seattle). Further, the existence of long branches separating several subclades within clade 1 is consistent with multiple introductions of clade 1 lineages into the United States (Supplementary Figure 3).

While clade 1 includes isolates from 2013, the last clade 2 isolate was observed in 2011 (Supplementary Table 1), suggesting that this clade no longer circulates in the United States. Given that isolates from clade 2 were identified in cases from 6 STD clinics (Supplementary Table 1), it seems unlikely that disappearance of this clade is due solely to entry into dead-end sex contact networks. Instead, the disappearance may have resulted from relative lack of fitness in the context of the increased treatment dose of ceftriaxone from 125 to 250 mg in 2010–2011 [15, 16], as all clade 2 isolates have a ceftriaxone MIC of ≤0.06 µg/mL.

The 2 ESC^RS^ isolates with the mosaic *penA* XXXIV that are not part of larger clades (Figure 2*A*)—GCGS0099 and GCGS0926—appear in 2010 and 2012, without evidence of continued spread. The nonmosaic *penA* ESC^RS^ (GCGS0870, GCGS0627, GCGS1029, GCGS1035, GCGS1013, GCGS1095, and GCGS1014) have appeared sporadically (during 2000 in Birmingham, during 2003 in Orange County, during 2007 in Cleveland, during 2007 in Detroit, during 2011 in Baltimore, during 2012 in Oklahoma City, and during 2012 in Chicago, respectively), and, other than the closely related isolates of GCGS1095 and GCGS1014, have no evidence of propagation.

### Reduced Susceptibility to Azithromycin

In contrast with ESC^RS^, reduced azithromycin susceptibility appears sporadically across the phylogeny (Figure 1), with 75 episodes (range, 69–84 episodes) of acquisition of resistance through de novo mutation or horizontal gene transfer inferred by ancestral state reconstruction. Azithromycin resistance has less clonal expansion (Figure 1*B*) and evidence of frequent reversion to susceptibility, with an inferred 42 episodes (range, 33–48 episodes) of loss of resistance.

**Figure 4.**
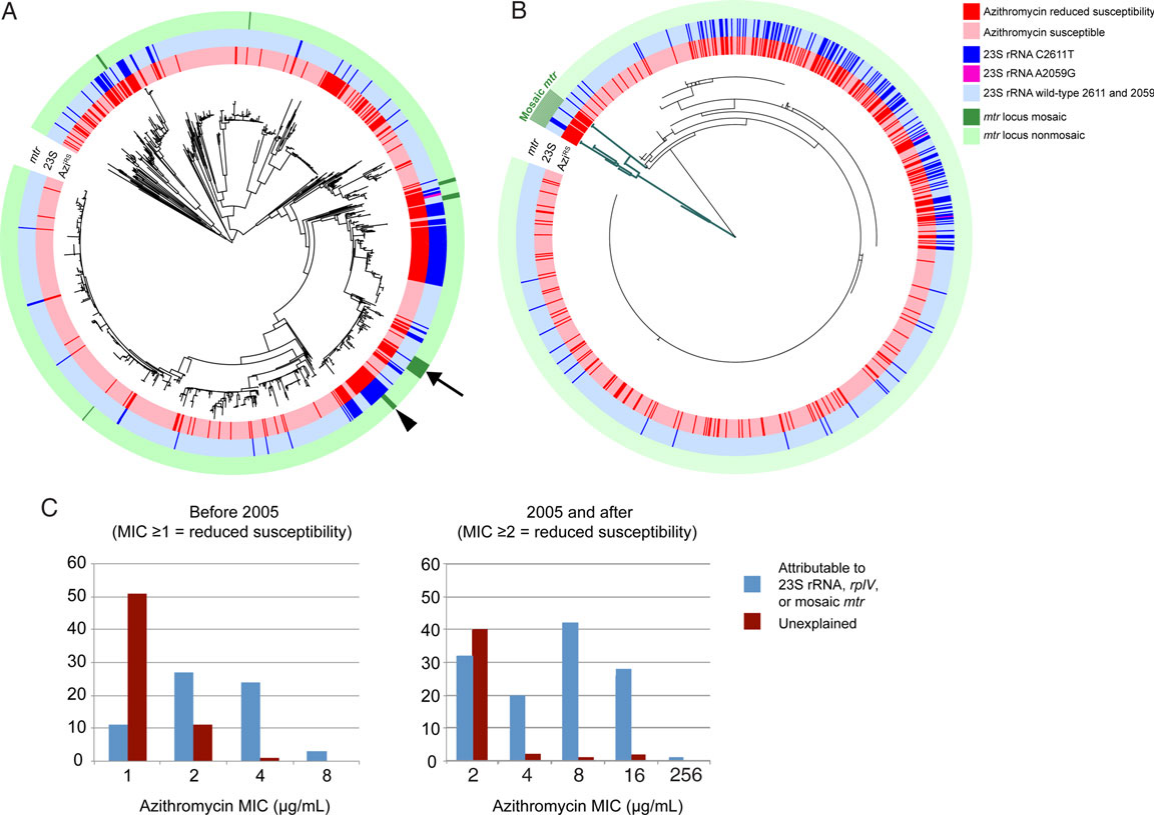
*A*, Maximum likelihood whole-genome-sequence phylogeny of 1102 *Neisseria gonorrhoeae* isolates, based on single-nucleotide polymorphisms from mapping to the FA1090 reference genome, with the inner annotation ring representing reduced azithromycin susceptibility, the middle annotation ring indicating isolates with at least 2 copies of the C2611T 23S ribosomal RNA (rRNA) mutation and 2 isolates with 4 copies of the A2059G 23S rRNA mutation, and the outer annotation ring indicating isolates with a mosaic *mtr* locus. The arrow indicates an example where the *mtr* locus mosaic is inferred to have appeared first, followed by acquisition of the C2611T mutation, and the wedge indicates an example with the opposite order of acquisition. *B*, Maximum likelihood phylogeny of the *mtrR* locus including the 200 base pairs upstream of the coding sequence start site, extracted from the de novo–assembled genomes for each of the isolates. The branches in green indicate isolates with mosaic *mtr* loci. As in panel *A*, the annotation rings proceed from the innermost being reduced azithromycin susceptibility to the outermost being the mosaic *mtr* loci. *C*, Histograms indicating the azithromycin minimum inhibitory concentrations (MICs), separated into 2 sections by when there was a change in the azithromycin MIC testing protocol, such that the cutoff changed from 1 to 2 µg/mL.

The 23S ribosomal RNA (rRNA) mutations C2611T (164 isolates with ≥2 mutated 23S rRNA alleles) and A2059G (2 isolates, in which all 4 alleles are mutant) are highly associated with resistance (Figures 3 and 4), as are interspecies mosaics in the *mtr* operon (across both *mtrCDE* and *mtrR*; Figures 3, 4, and Supplementary Figure 4) encoding the Mtr efflux pump. We observe 7 events of likely interspecies recombination (Figure 4*A* and 4*B*), each of which is associated with acquisition of azithromycin resistance. This includes a set of 4 isolates from Kansas City collected in 2000 that possess an *mtr* mosaic with an *mtrR* sequence that matches perfectly to *N. meningitidis*, with presence of a Correia element corresponding with a reported outbreak of Azi^RS^ in Missouri in 1999 [17]. The subset of 5 isolates that contain both 23S rRNA C2611T mutations and *mtr* locus mosaics have higher MICs (8–16 µg/mL) than those that contain the *mtr* locus mosaics alone (1–4 µg/mL).

**Figure 5.**
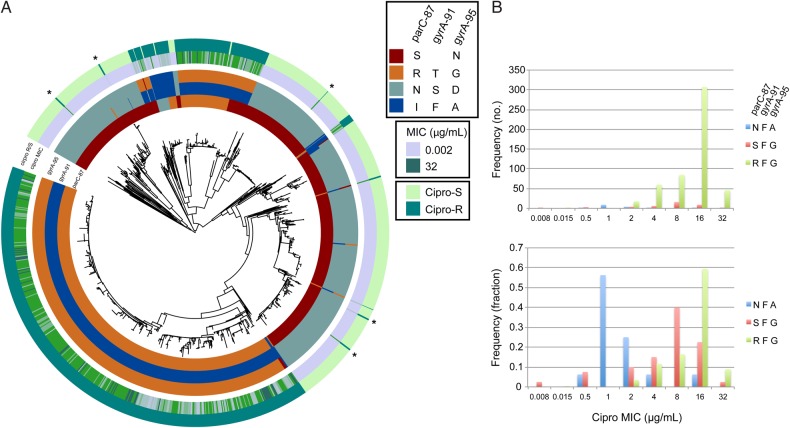
*A*, Maximum likelihood whole-genome-sequence phylogeny of 1102 *Neisseria gonorrhoeae* isolates, based on single-nucleotide polymorphisms from mapping to the FA1090 reference genome, with the inner annotation rings representing amino acid residues at ParC-87, GyrA-91, and GyrA-95. The outer annotation rings represent ciprofloxacin (Cipro) minimum inhibitory concentrations (MICs) and dichotomized resistance (R) and susceptibility (S; cutoff for resistance at 1 µg/mL). The asterisks indicate quinolone-resistant *N. gonorrhoeae* that lack the ParC-87, GyrA-91, and GyrA-95 variants. *B*, Histograms of Cipro MICs of isolates based on haplotypes at ParC-87, GyrA-91, and GyrA-95 in absolute frequency (upper histogram) and in fraction (lower histogram).

In contrast, coding and promoter mutations in the *mtrR* locus that have been associated with increased MICs to macrolides [11] are not associated here with Azi^RS^ (Figure 5), suggesting that genomic background may influence the impact of these variants on azithromycin resistance. A single nucleotide promoter variant upstream of the *mtrC* start codon that is associated with resistance [18] was not observed in this data set.

Mutations in ribosomal proteins L4 (*rplD*) and L22 (*rplV*) yield macrolide resistance [19–21] in other bacteria but have not been previously reported in gonococcus. We identify 2 Azi^RS^ isolates (GCGS0838 and GCGS1026; MIC = 16 µg/mL) that have 6 and 4 amino acid tandem duplications, respectively, in the 3′ end of *rplV* and are predicted to interact with the azithromycin binding site, similar to resistance-conferring insertions described in other organisms (Supplementary Figure 5) [20, 21]. We identify isolates with mutations at G68 (n = 9) and G70 (n = 57) in an *rplD* region previously associated with macrolide resistance. However, only 58% of isolates with either mutation are Azi^RS^, and these have MICs at or just above the resistance threshold, with the exception of those that additionally possess 23S rRNA or *mtr* mosaic variants.

Together, the 23S rRNA mutations, *mtr* locus mosaics, and *rplV* variants account for 188 of 294 isolates (64%) with reduced azithromycin susceptibility (Figure 4*C*). The mechanisms accounting for Azi^RS^ in the remaining 36% are unclear. None of the other genes known or postulated to be involved in resistance that we evaluated (including *mtrA*, *norM*, or *macA/B* [11, 22]) were highly associated with azithromycin resistance (Supplementary Table 2), and we did not observe *ermB*, *ermC*, or *ermF* in our data set. Notably, unexplained resistance appears primarily in isolates with low-level resistance, with 81% of isolates with unexplained resistance having an MIC at the threshold (Figure 4*C*).

Intriguingly, 17 of 19 isolates (89%) containing the *rplD* 70 mutation, with *porB* 121D and *macA* 99N but lacking known 23S rRNA or mosaic *mtr* variants, have reduced azithromycin susceptibility. We hypothesize that these mutations may reflect multiple mechanisms that additively contribute to elevated MICs. While experiments would be required to test this hypothesis, its plausibility is enhanced by the fact that this resistant phenotype with the trio of mutations described appears in 2 separate clonal groups.

The temporal and geographic patterns together with the phylogeny suggest that the azithromycin resistance variants appear frequently, although only some propagate enough to leave multiple descendants in our data set (Figure 4*A*). The *mtr* mosaics, for example, represent 7 distinct recombination episodes (Supplementary Figure 3), each present for 1–2 years in our sampling period, with cluster 3 the only cluster present in 2013 (Supplementary Table 3). Only the largest cluster has spread beyond 2 cities, with expansion in Miami, Philadelphia, and Dallas. The C2611T 23S rRNA variants have expanded clonally but also show multiple instances of no detectable propagation.

### Quinolone Resistance

Phylogenetic analysis and ancestral state reconstruction indicate that quinolone resistance has emerged 11 times (range, 10–12 times) through de novo mutation or recombination (Figures 1 and 5). The few instances of inferred loss of resistance (7; range, 5–8 instances) despite the number of QRNG in this data set is consistent with the observation that most quinolone resistance mutations are associated with limited, if any, fitness cost [23].

In *N. gonorrhoeae*, quinolone resistance has been attributed to variants in *gyrA* and *parC*, including GyrA amino acid positions 91 and 95 and ParC position 87. Specific amino acid residues at these sites are highly predictive of resistance phenotype in our data set (Figure 3). ParC sites 86, 88, and 91 have been reported as varying in QRNG [24]. In our data set, ParC-86 variant D86N appears only 7 times and S88P only once. E91G appears 28 times and only in the context of QRNG with ParC-87S; there is 1 isolate with E91K.

We evaluated the association between haplotypes at the ParC-87, GyrA-91, and GyrA-95 loci and ciprofloxacin MIC (Figure 5*B*). The 3 most common resistant haplotypes are ParC-97N/GyrA-91F/GyrA-95A (“NFA”; n = 16), ParC-87S/GyrA-91F/GyrA-95G (“SFG”; n = 40), and ParC-87R/GyrA-91F/GyrA-95G (“RFG”; n = 515). The geometric mean MIC for SFG is 5.2, whereas for RFG it is 11.9; in contrast, the NFA haplotype is associated with low-level resistance (MICs predominantly 1–2 µg/mL; Figure 5*B*).

Five resistant isolates do not have the characteristic GyrA or ParC variants (GCGS1019, GCGS0850, GCGS1043, GCGS0807, and GCGS0641; starred in Figure 5), suggesting that variants at other loci can also yield quinolone resistance. The rare appearance of these isolates and their lack of propagation suggest that they carry a high fitness cost.

### Multidrug Resistance and Interactions Among Resistance Mechanisms

Five isolates have reduced susceptibility to the ESCs and to azithromycin, and 4 of these isolates exceed the MIC cutoffs for resistance to quinolones (Supplementary Table 1). While these cases have not come to attention as treatment failures, it is possible that, with treatment, (1) the bacterial burden was decreased to the extent that the infections were no longer symptomatic, (2) the infections were cured despite the MICs, and (3) there was no ongoing transmission.

The apparent anticorrelation between azithromycin and ESC resistance in Figure 1 raises the hypothesis that the mechanism for resistance to azithromycin might impact MICs to other antibiotics. Therefore, we tested whether resistance to other drugs was altered in the context of the 23S rRNA C2611T mutation as the most common mechanism of azithromycin resistance. To account for varying genomic background, we performed these tests within BAPS groups; 3 had >10 isolates with the 23S rRNA C2611T mutation (BAPS-4, n = 64; BAPS-7, n = 41; and BAPS-11, n = 18). In BAPS-4, the MICs for cefixime, ceftriaxone, and ciprofloxacin (*P* < .01 for each, by the Mann–Whitney *U* test including Bonferroni correction) and in BAPS-7 the MICs for cefixime and ceftriaxone (*P* = .008 and *P* = .02, respectively) were significantly lower in the presence of the 23S rRNA C2611T mutation; however, in BAPS-7 the ciprofloxacin MIC and in BAPS-11 cefixime, ceftriaxone, and ciprofloxacin MICs were not significantly different. These results could result from the sampling strategy or could represent biological interactions among resistance mechanisms.

## DISCUSSION

Application of genotype-based resistance prediction tools depends on the prevalence and reliability of the genetic markers of resistance. Here, we used a retrospective longitudinal phylogenomic approach to define the distribution of resistance markers to the 3 most clinically relevant classes of antimicrobials for treatment of gonococcal infections.

The observation that ESC^RS^ is highly although not exclusively associated with the mosaic *penA* XXXIV allele and its derivatives affirms previous results from data from gonococcal populations in the United States and Canada [1, 25]. We note several additional findings. First, resistance mechanisms that yield ESC^RS^ in the absence of a mosaic *penA* XXXIV–type allele have appeared sporadically (Figure 2*B*). The mechanism for resistance in these isolates is not clear; they do not share *penA* or *porB* alleles (Figure 2*B* and Supplementary Figure 2). Second, we do not observe mosaic *penA* alleles other than XXXIV and derivatives that are associated with ESC resistance in this population (Figure 2*B*). Third, in contrast with cefixime, resistance to ceftriaxone appears to involve loci in addition to *penA*, although these loci remain to be identified (Figure 2*C*).

ESC^RS^ has spread predominantly through clonal expansion, with 97% of ESC^RS^ isolates belonging to one of two BAPS groups and possessing the mosaic *penA* XXXIV type (Figures 1*B* and 2). Of the 7 ESC^RS^ that do not possess a mosaic *penA* XXXIV–type allele, 2 appear closely related phylogenetically, suggesting at least 6 episodes of de novo emergence over the sampling period (2005–13).

Azi^RS^ is associated with multiple mechanisms of resistance. We identify variants of the ribosome, including known 23S rRNA mutations (C2611T [n = 163] and A2059G [n = 2]; total, 56% of all isolates with reduced azithromycin susceptibility) and previously unreported *rplV* mutations (n = 2 [<1%]). We also identify interspecies mosaic variants of the efflux pump–encoding *mtr* locus (n = 29 [10%]), with 5 isolates containing both the mosaic *mtr* locus and C2611T mutations. Demczuk et al recently found *ermB* and *ermC* in 3 Azi^RS^ isolates in Canada [26], but we found neither of these genes. Notably, many of the *mtrR* variants reported in the literature to result in Azi^RS^ were not highly associated with resistance in this data set. As 36% of Azi^RS^ isolates did not possess alleles that were highly associated with resistance, it is possible that these isolates contain as yet undefined mechanisms of resistance or combinations of loci that additively yield resistance. That multiple loci can combine to yield higher levels of resistance is suggested by MICs for isolates that possess both *mtr* mosaic alleles and 23S rRNA mutations as compared to MICs of the isolates containing the *mtr* mosaics alone. Further, co-occurring alleles of *rplD*, *porB*, and *macA* that are associated with reduced azithromycin susceptibility suggest another possible set of loci that may combinatorially influence azithromycin MIC.

Azi^RS^ appears sporadically more often than the other phenotypes we studied, perhaps indicating that treatment readily selects for Azi^RS^ variants. Likewise, the limited clonal spread as compared to ESC^RS^ and quinolone resistance (Figure 1), as well as the inferred frequent return to susceptibility, implies that azithromycin resistance often incurs significant fitness costs. However, recent reports of an outbreak of azithromycin-resistant gonorrhea in England [27] and an increase in azithromycin resistance in the United States [8] raise concern that these fitness costs may be mitigated in some genomic backgrounds.

Quinolone resistance in this data set is primarily clonal and is highly correlated with *gyrA* and *parC* mutations. Haplotypes of key GyrA and ParC amino acids yield distinct MIC distributions, suggesting that genotype may be used in predicting the extent of resistance. While the vast majority of QRNG possess *gyrA* or *parC* mutations associated with resistance (589 of 594 [>99%]), 5 QRNG do not; the mechanisms of resistance in these instances are unclear.

There are several limitations to this study. As GISP isolates are collected only from men with gonococcal urethritis, it is unclear to what extent the specimens studied here are representative of those from other mucosal sites of infection. Although GISP clinics are distributed across the United States, their representativeness of all cases of gonorrhea in the United States is unclear. The selection for this study of isolates on the basis of their antimicrobial susceptibility profile may skew prevalence estimates, particularly as susceptible comparators for any given drug were usually available for study because they were resistant to another antimicrobial; pan-susceptible isolates are not typically frozen for storage following isolation in GISP. Additionally, susceptibility testing has a margin of error of ±1 dilution, although the very high positive predictive values for genetic markers for resistance to each of the antibiotics suggest a limited impact of MIC error.

The identification of isolates with reduced susceptibility to both azithromycin and the ESCs underscores the imminent risk of treatment-resistant infections and the importance of novel strategies to diagnose and treat gonococcal infections, including use of rapid sequence-based assays to determine antibiotic susceptibility and allow for reintroduction of quinolones and other antibiotics into practice guidelines. The observed diversity and change over time in the mechanisms of resistance, with some remaining to be described, also emphasize the critical need for continued surveillance of phenotypic antibiotic resistance.

## Supplementary Data

Supplementary materials are available at http://jid.oxfordjournals.org. Consisting of data provided by the author to benefit the reader, the posted materials are not copyedited and are the sole responsibility of the author, so questions or comments should be addressed to the author.

Supplementary Data
