# Supplementary material for: Genomic Epidemiology of Gonococcal Resistance to Extended-Spectrum Cephalosporins, Macrolides, and Fluoroquinolones in the United States, 2000–2013
Source: J Infect Dis. 2016 Sep 16;214(10):1579–87. doi: 10.1093/infdis/jiw420 (PMC5091375; doi:10.1093/infdis/jiw420)
Supplement: Supplementary Data [file supp_214_10_1579__index.html]

Genomic epidemiology of gonococcal resistance to extended spectrum cephalosporins, macrolides, and fluoroquinolones in the US, 2000-2013 — Genomic Epidemiology of Gonococcal Resistance to Extended-Spectrum Cephalosporins, Macrolides, and Fluoroquinolones in the United States, 2000–2013 — Genomic Epidemiology of Gonococcal Resistance to Extended-Spectrum Cephalosporins, Macrolides, and Fluoroquinolones in the United States, 2000–2013 — Supplementary Data 

# Genomic Epidemiology of Gonococcal Resistance to Extended-Spectrum Cephalosporins, Macrolides, and Fluoroquinolones in the United States, 2000–2013

## Supplementary Data

Supplementary Data

- Supplementary Data - Docx file
