# Supplementary material for: Genomic Epidemiology of Gonococcal Resistance to Extended-Spectrum Cephalosporins, Macrolides, and Fluoroquinolones in the United States, 2000–2013
Source: J Infect Dis. 2016 Sep 16;214(10):1579–87. doi: 10.1093/infdis/jiw420 (PMC5091375; doi:10.1093/infdis/jiw420)
Supplement: Supplementary Data [file supp_jiw420_jiw420supp.docx]

**Supplemental Material For:**

**Genomic epidemiology of gonococcal resistance to extended spectrum cephalosporins, macrolides, and fluoroquinolones in the US, 2000-2013**

Yonatan H. Grad MD, PhD ^1,2,a^, Simon R. Harris PhD ^3^, Robert D. Kirkcaldy MD ^4^, Anna G. Green BS ^5^, Debora S. Marks PhD ^5^, Stephen D. Bentley PhD ^3,6^, David Trees PhD ^4,^*, Marc Lipsitch DPhil ^1,7,^*

| **Items** | **Pages** |
| --- | --- |
| **Supplemental Figure 1.** | **2** |
| **Supplemental Figure 2.** | **3** |
| **Supplemental Figure 3.** | **4-7** |
| **Supplemental Figure 4.** | **8-9** |
| **Supplemental Table 1.** | **10-35** |
| **Supplemental Table 2.** | **36-43** |
| **Supplemental Table 3.** | **44-45** |
| **Supplemental Table 4.** | **46** |

**Supplemental Figure 1.** Map of the US with the locations of the clinics from which samples were obtained. The size of the circle is proportional to the number of isolates contributed from each site. The pie charts reflect the fraction from each site from men who have sex with men (MSM) in red, men who have sex with women (MSW) in blue, and bisexual in green.

**Supplemental Figure 2.** Maximum likelihood phylogeny on left, with categorical reduced susceptibility or resistance to extended spectrum cephalosporins (ESCs), azithromycin (AZI), and ciprofloxacin (CIP), as well as year of isolation and reported sexual orientation of the infected individual (MSM=men who have sex with men; MSW=men who have sex with women; MSMW=men who have sex with men and women). The cluster of isolates representing the first appearance in the dataset of the mosaic *penA* XXXIV are highlighted in light green.

**
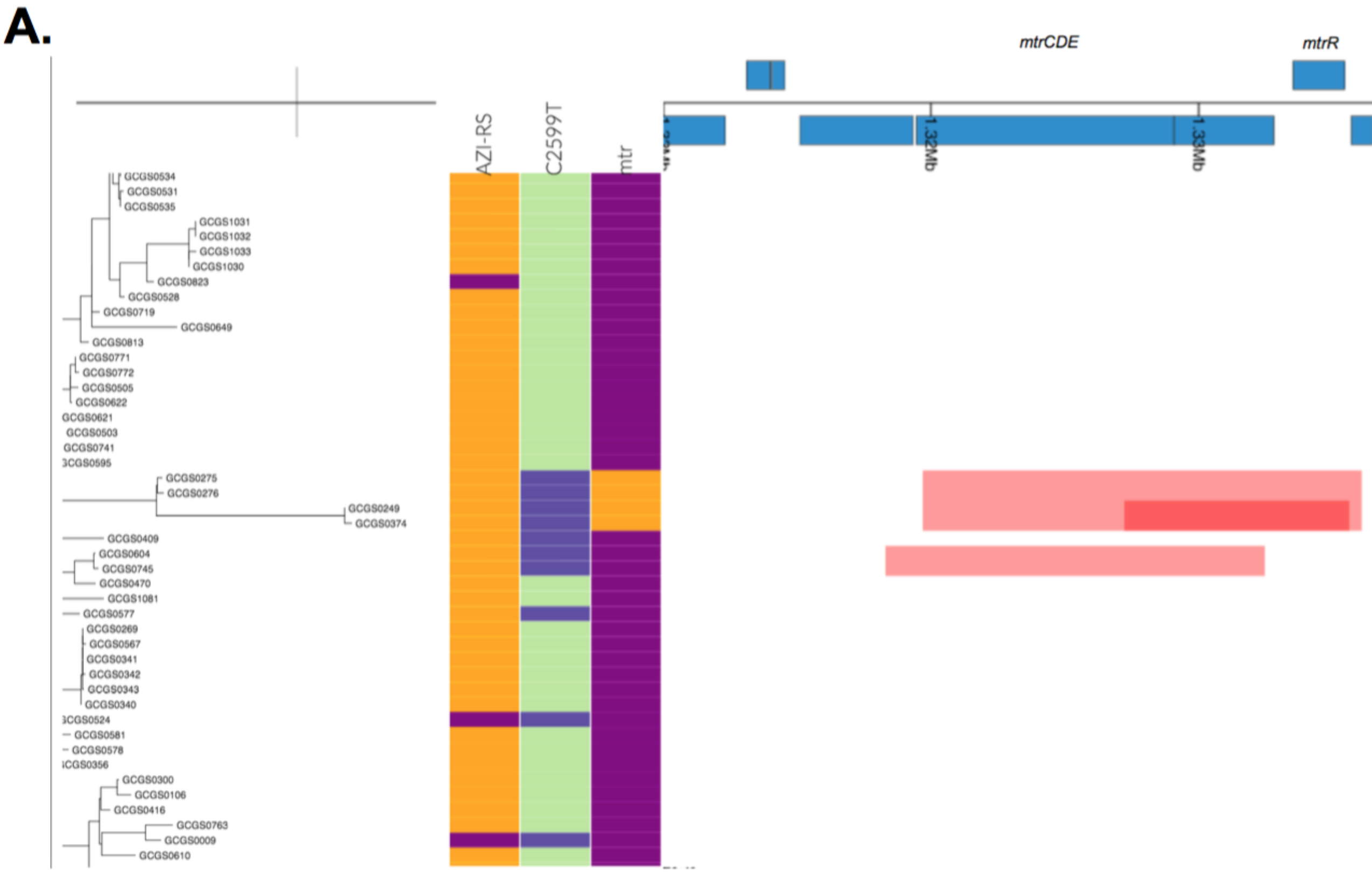
**

**
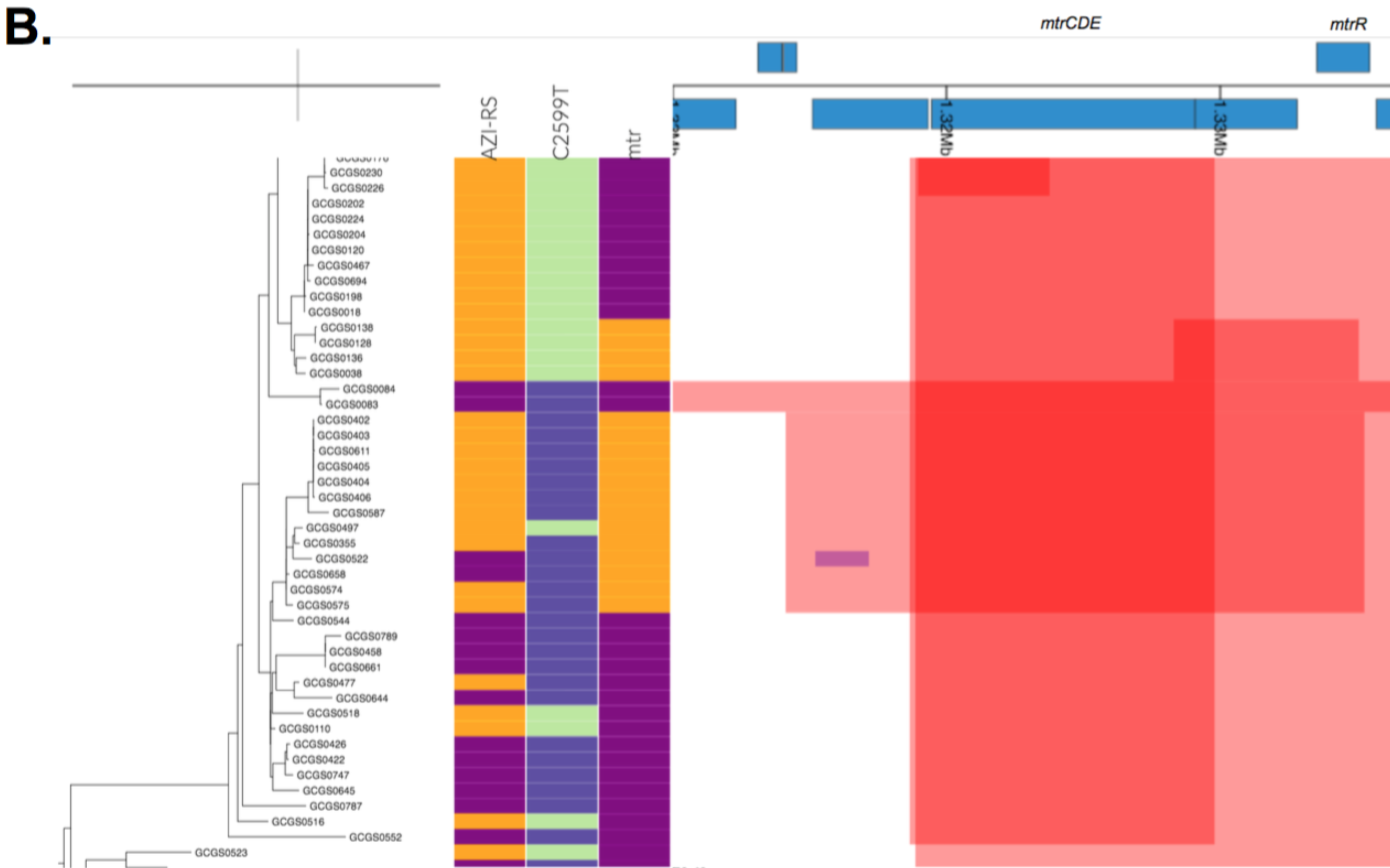
**

**
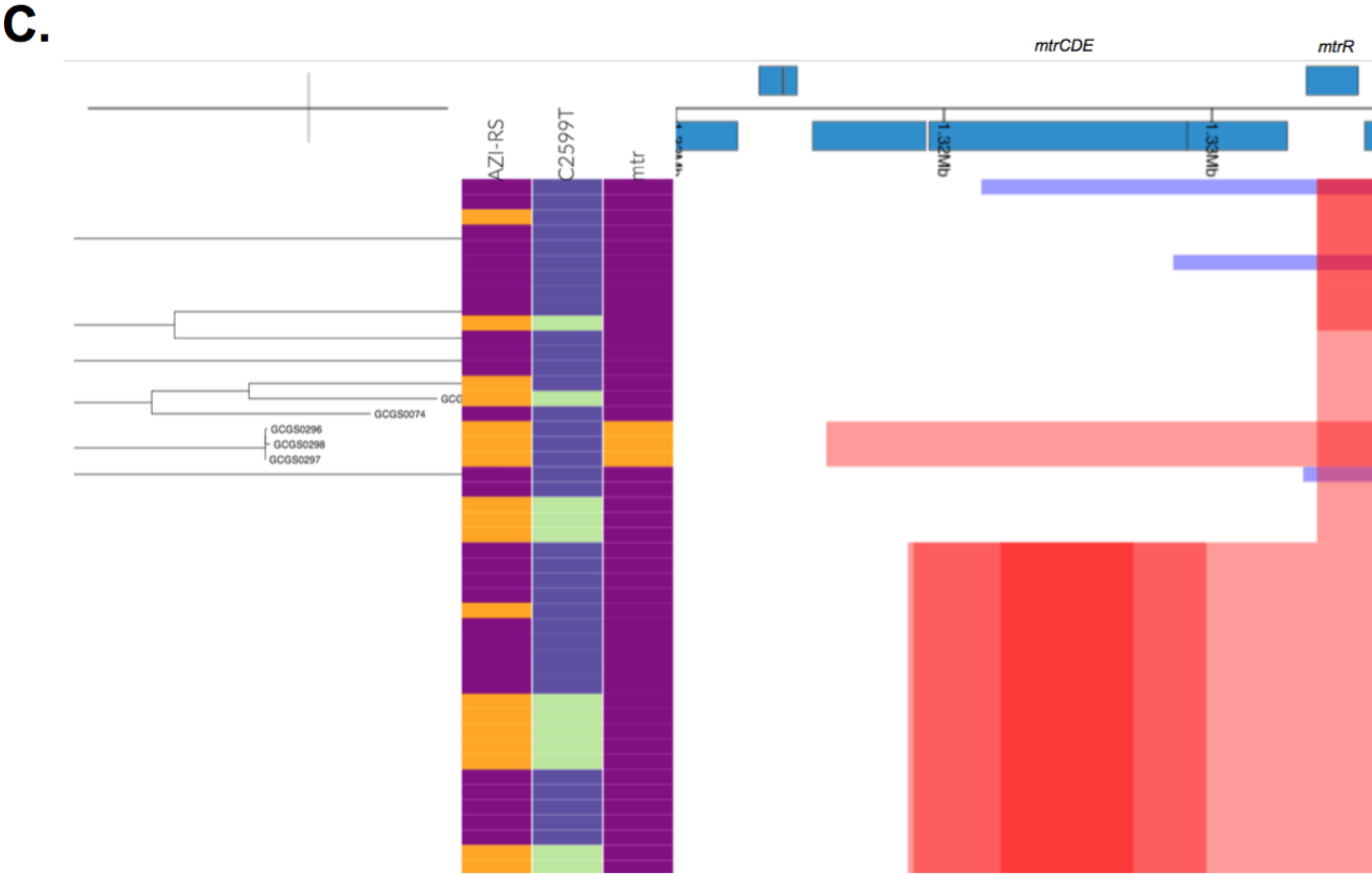
** **
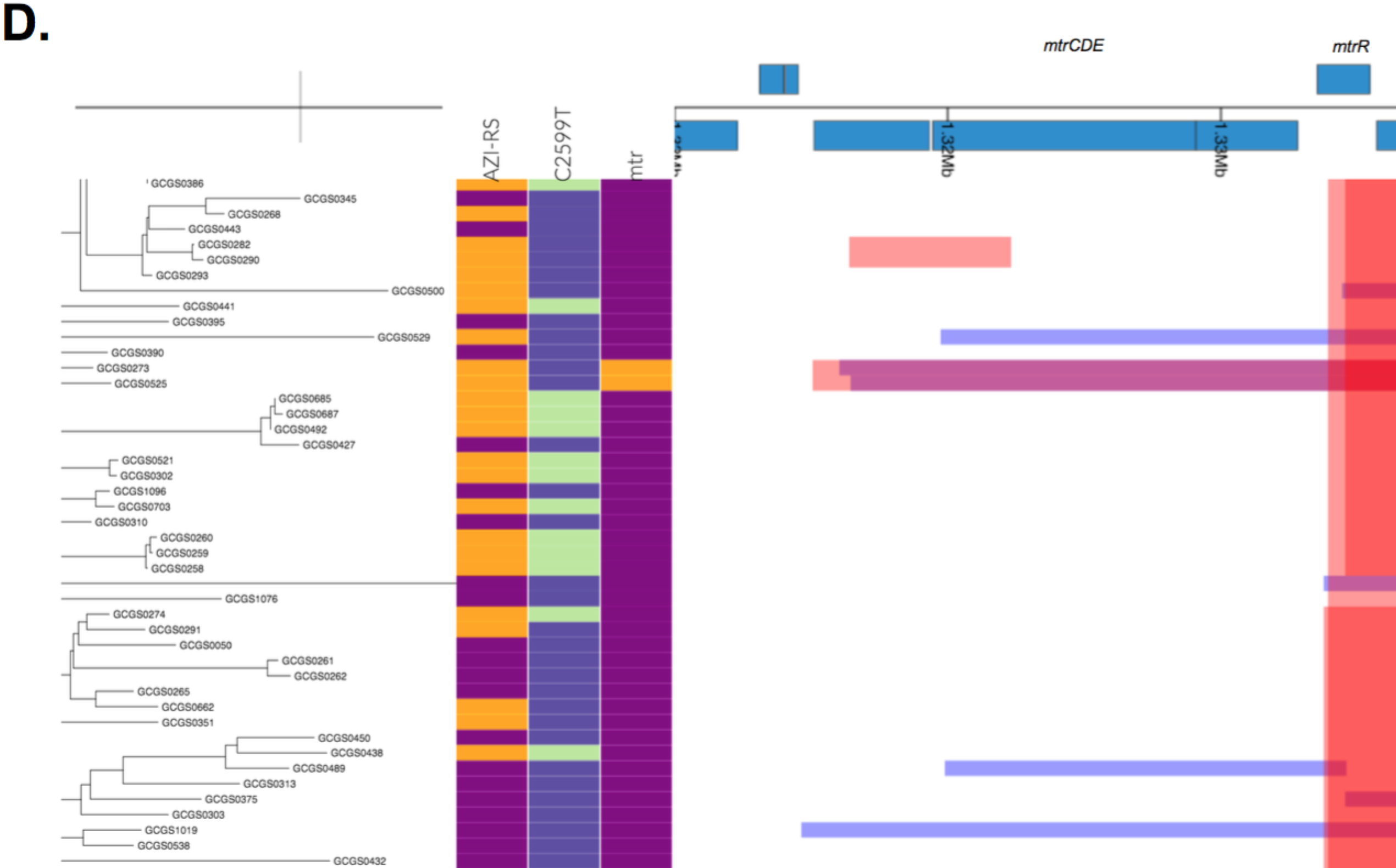
**

**
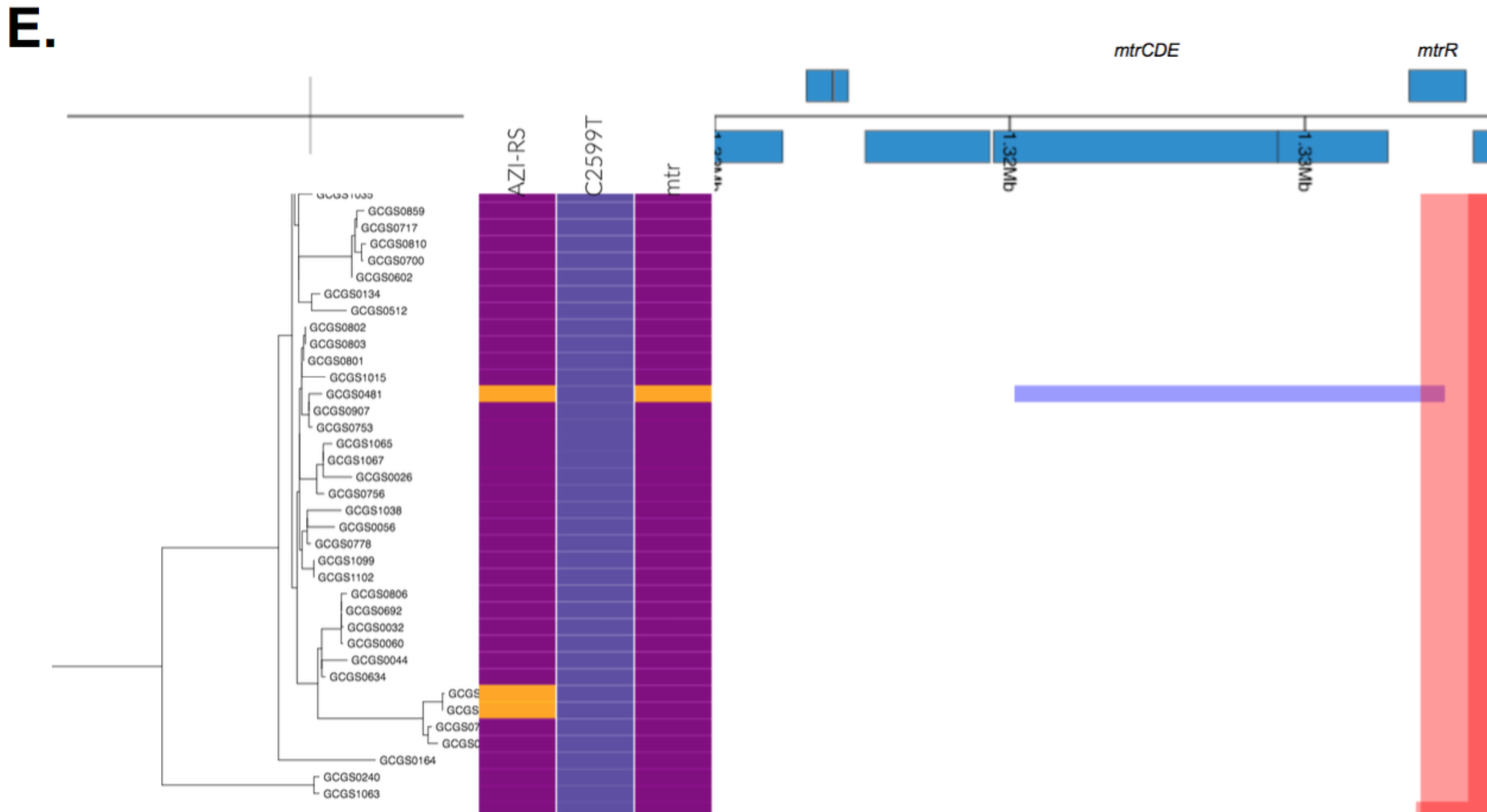
** **
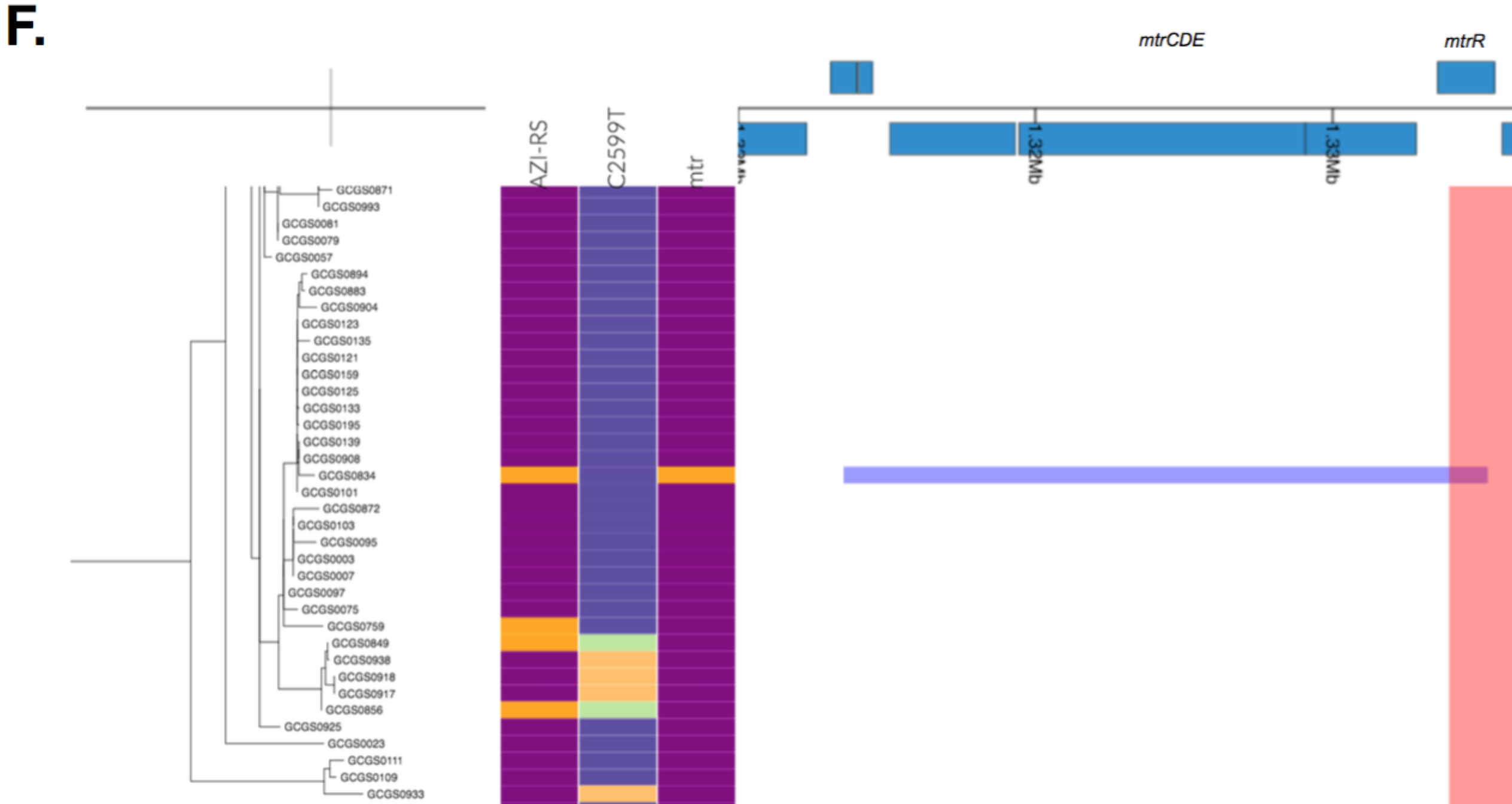
**

**Supplemental Figure 3.** Predicted recombinations at the *mtr* locus. The left side of each figure represents a zoomed-in view of the maximum likelihood whole-genome phylogeny; the three columns mid-figure represent, from left to right, resistance (in yellow) or susceptibility (in purple) to azithromycin (AZI-RS); wild-type (light green) or ≥2/4 23S rRNA alleles with mutant C2599T (blue); and presence (yellow) or absence (purple) of *mtr* locus mosaic. The right side of the figure is annotated at top by gene position in the *N. gonorrhoeae* reference genome FA1090, and the red (when predicted recombination event is shared by more than one isolate) and blue (when predicted recombination event is in a terminal branch) rectangles indicate regions of recombination as predicted by Gubbins [10]. **A.** Cluster 1 (see Supplemental Table 6). **B.** Cluster 2 and 3. **C.** Cluster 4. **D.** Cluster 5. **E.** Isolate 6. **F.** Isolate 7. Figures generated using Phandango (<http://jameshadfield.github.io/phandango/>).

**Supplemental Figure 4. A.** Structure of the RplV protein in complex with the 23S ribosomal RNA. We assessed RplV in its ribosomal context using the structure of the azithromycin-bound 50S ribosomal complex from *Thermus thermophilus* (PDB ID: 4v7y). This structure is high resolution (3.0 Å), and the *T. thermophilus* RplV sequence is highly similar to the *N. gonorrhoeae* sequence (47% identical, 64% similar over 113 amino acids with 0 insertions/deletions). We modeled the two tandem duplications (ARAK at position 87-90 and KGPSLK at position 78-83) by editing the sequence and structure in Pymol (The PyMOL Molecular Graphics System, Version 1.7.4 Schrödinger, LLC) ensuring acceptable bond angles. The sites of the ARAK and KGPSLK duplications are colored in green and cyan, respectively. **B, C.** Insertion of ARAK (green; **B**) and KGPSLK (cyan; **C**) residues yield steric clashes with azithromycin.

**Supplemental Table S1.**

| Identifier | Cefixime MIC (µg/mL) | Ceftriaxone MIC (µg/mL) | Ciprofloxacin MIC (µg/mL) | Azithromycin MIC (µg/mL) | Clinic site | Year | Sex of sex partner | mosaic penA  XXXIV derivative (1=present; 0=absent) | TEM beta-lactamase (1=present; 0=absent) |
| --- | --- | --- | --- | --- | --- | --- | --- | --- | --- |
| GCGS0920 | 0.25 | 0.125 | 8 | 0.5 | MIA | 2005 | MSW | 1 | 0 |
| GCGS0944 | 0.25 | 0.06 | 16 | 0.5 | POR | 2005 | MSW | 1 | 0 |
| GCGS0930 | 0.25 | 0.03 | 16 | 1 | ORA | 2006 | MSW | 1 | 0 |
| GCGS0921 | 0.25 | 0.06 | 16 | 0.5 | MIA | 2006 | MSMW | 1 | 0 |
| GCGS1042 | 0.25 | 0.125 | 16 | 1 | LAX | 2007 | MSW | 1 | 0 |
| GCGS1049 | 0.125 | 0.125 | 16 | 1 | LVG | 2007 | MSW | 1 | 0 |
| GCGS1051 | 0.125 | 0.063 | 16 | 1 | LVG | 2007 | MSW | 1 | 0 |
| GCGS1052 | 0.125 | 0.063 | 16 | 1 | LVG | 2007 | MSW | 1 | 0 |
| GCGS1056 | 0.125 | 0.063 | 16 | 1 | LVG | 2007 | MSW | 1 | 0 |
| GCGS1057 | 1 | 0.25 | 6 | 0.5 | LVG | 2008 | MSW | 1 | 0 |
| GCGS1078 | 0.25 | 0.125 | 16 | 0.5 | PHX | 2008 | MSW | 1 | 0 |
| GCGS1068 | 0.125 | 0.063 | 16 | 0.5 | ORA | 2008 | MSM | 1 | 0 |
| GCGS0005 | 0.5 | 0.06 | 16 | 1 | CHI | 2009 | MSM | 1 | 0 |
| GCGS0031 | 0.5 | 0.03 | 32 | 0.5 | HON | 2009 | MSM | 1 | 0 |
| GCGS0037 | 0.5 | 0.125 | 32 | 0.5 | LAX | 2009 | MSM | 1 | 0 |
| GCGS0061 | 0.5 | 0.06 | 32 | 0.5 | POR | 2009 | MSM | 1 | 0 |
| GCGS0001 | 0.25 | 0.125 | 16 | 1 | CHI | 2009 | MSM | 1 | 0 |
| GCGS0003 | 0.25 | 0.125 | 16 | 1 | CHI | 2009 | MSM | 1 | 0 |
| GCGS0017 | 0.25 | 0.06 | 32 | 1 | HON | 2009 | MSM | 1 | 0 |
| GCGS0019 | 0.25 | 0.06 | 32 | 1 | HON | 2009 | MSM | 1 | 0 |
| GCGS0021 | 0.25 | 0.06 | 32 | 1 | HON | 2009 | MSM | 1 | 0 |
| GCGS0035 | 0.25 | 0.125 | 32 | 1 | LAX | 2009 | MSW | 1 | 0 |
| GCGS0039 | 0.25 | 0.125 | 16 | 1 | LVG | 2009 | MSW | 1 | 0 |
| GCGS0041 | 0.25 | 0.125 | 16 | 1 | LVG | 2009 | MSMW | 1 | 0 |
| GCGS0043 | 0.25 | 0.06 | 16 | 1 | LVG | 2009 | MSW | 1 | 0 |
| GCGS0045 | 0.25 | 0.06 | 16 | 1 | LVG | 2009 | MSW | 1 | 0 |
| GCGS0047 | 0.25 | 0.06 | 16 | 1 | LVG | 2009 | MSW | 1 | 0 |
| GCGS0049 | 0.25 | 0.06 | 16 | 1 | LVG | 2009 | MSW | 1 | 0 |
| GCGS0053 | 0.25 | 0.125 | 16 | 1 | LVG | 2009 | MSW | 1 | 0 |
| GCGS0055 | 0.25 | 0.06 | 16 | 1 | PHI | 2009 | MSM | 1 | 0 |
| GCGS0059 | 0.25 | 0.125 | 16 | 1 | PHX | 2009 | MSM | 1 | 0 |
| GCGS0069 | 0.25 | 0.125 | 16 | 1 | SDG | 2009 | MSW | 1 | 0 |
| GCGS0077 | 0.25 | 0.06 | 16 | 1 | SDG | 2009 | MSW | 1 | 0 |
| GCGS0089 | 0.25 | 0.03 | 32 | 1 | SFO | 2009 | MSM | 1 | 0 |
| GCGS0007 | 0.25 | 0.06 | 16 | 0.5 | CHI | 2009 | MSM | 1 | 0 |
| GCGS0023 | 0.25 | 0.03 | 16 | 0.5 | HON | 2009 | MSW | 1 | 0 |
| GCGS0025 | 0.25 | 0.06 | 16 | 0.5 | HON | 2009 | MSM | 1 | 0 |
| GCGS0027 | 0.25 | 0.03 | 32 | 0.5 | HON | 2009 | MSM | 1 | 0 |
| GCGS0029 | 0.25 | 0.06 | 32 | 0.5 | HON | 2009 | MSM | 1 | 0 |
| GCGS0051 | 0.25 | 0.06 | 16 | 0.5 | LVG | 2009 | MSW | 1 | 0 |
| GCGS0057 | 0.25 | 0.06 | 16 | 0.5 | PHX | 2009 | MSM | 1 | 0 |
| GCGS0063 | 0.25 | 0.06 | 16 | 0.5 | POR | 2009 | MSMW | 1 | 0 |
| GCGS0065 | 0.25 | 0.06 | 16 | 0.5 | POR | 2009 | MSM | 1 | 0 |
| GCGS0067 | 0.25 | 0.125 | 16 | 0.5 | SDG | 2009 | MSM | 1 | 0 |
| GCGS0071 | 0.25 | 0.03 | 0.015 | 0.5 | SDG | 2009 | MSM | 1 | 0 |
| GCGS0075 | 0.25 | 0.06 | 8 | 0.5 | SDG | 2009 | MSM | 1 | 0 |
| GCGS0079 | 0.25 | 0.06 | 16 | 0.5 | SEA | 2009 | MSM | 1 | 0 |
| GCGS0085 | 0.25 | 0.03 | 32 | 0.5 | SFO | 2009 | MSM | 1 | 0 |
| GCGS0087 | 0.25 | 0.03 | 32 | 0.5 | SFO | 2009 | MSW | 1 | 0 |
| GCGS0033 | 0.25 | 0.06 | 16 | 0.25 | LAX | 2009 | MSW | 1 | 0 |
| GCGS0073 | 0.25 | 0.06 | 0.015 | 0.25 | SDG | 2009 | MSMW | 1 | 0 |
| GCGS0081 | 0.25 | 0.06 | 16 | 0.125 | SEA | 2009 | MSM | 1 | 0 |
| GCGS0844 | 0.125 | 0.06 | 16 | 4 | ORA | 2009 | MSM | 1 | 0 |
| GCGS0046 | 0.125 | 0.063 | 16 | 1 | LVG | 2009 | MSW | 1 | 0 |
| GCGS0088 | 0.125 | 0.063 | 16 | 0.5 | SFO | 2009 | MSW | 1 | 0 |
| GCGS0835 | 0.125 | 0.06 | 16 | 0.5 | LVG | 2009 | MSW | 1 | 0 |
| GCGS0847 | 0.125 | 0.03 | 8 | 0.5 | PHI | 2009 | MSW | 1 | 0 |
| GCGS0851 | 0.125 | 0.06 | 8 | 0.5 | PHX | 2009 | MSM | 1 | 0 |
| GCGS0099 | 0.5 | 0.25 | 16 | 1 | CLE | 2010 | MSW | 1 | 0 |
| GCGS0097 | 0.5 | 0.125 | 16 | 0.5 | CHI | 2010 | MSM | 1 | 0 |
| GCGS0115 | 0.5 | 0.06 | 16 | 0.5 | HON | 2010 | MSM | 1 | 0 |
| GCGS0121 | 0.5 | 0.06 | 16 | 0.5 | LAX | 2010 | MSM | 1 | 0 |
| GCGS0135 | 0.5 | 0.03 | 16 | 0.5 | LAX | 2010 | MSM | 1 | 0 |
| GCGS0213 | 0.5 | 0.063 | 0.004 | 0.5 | SDG | 2010 | MSM | 1 | 0 |
| GCGS0239 | 0.5 | 0.125 | 16 | 0.5 | SFO | 2010 | MSM | 1 | 0 |
| GCGS0241 | 0.5 | 0.125 | 16 | 0.5 | SFO | 2010 | MSM | 1 | 0 |
| GCGS0179 | 0.5 | 0.06 | 16 | 0.25 | POR | 2010 | MSM | 1 | 0 |
| GCGS0181 | 0.5 | 0.125 | 16 | 0.25 | POR | 2010 | MSM | 1 | 0 |
| GCGS0091 | 0.25 | 0.06 | 16 | 1 | CHI | 2010 | MSM | 1 | 0 |
| GCGS0093 | 0.25 | 0.125 | 16 | 1 | CHI | 2010 | MSM | 1 | 0 |
| GCGS0095 | 0.25 | 0.06 | 16 | 1 | CHI | 2010 | MSM | 1 | 0 |
| GCGS0139 | 0.25 | 0.125 | 32 | 1 | LAX | 2010 | MSM | 1 | 0 |
| GCGS0145 | 0.25 | 0.06 | 16 | 1 | LVG | 2010 | MSM | 1 | 0 |
| GCGS0147 | 0.25 | 0.125 | 16 | 1 | LVG | 2010 | MSW | 1 | 0 |
| GCGS0151 | 0.25 | 0.06 | 16 | 1 | LVG | 2010 | MSW | 1 | 0 |
| GCGS0155 | 0.25 | 0.125 | 16 | 1 | MIN | 2010 | MSM | 1 | 0 |
| GCGS0157 | 0.25 | 0.125 | 16 | 1 | NYC | 2010 | MSM | 1 | 0 |
| GCGS0165 | 0.25 | 0.06 | 16 | 1 | PHX | 2010 | MSM | 1 | 0 |
| GCGS0173 | 0.25 | 0.06 | 0.015 | 1 | PHX | 2010 | MSM | 1 | 0 |
| GCGS0177 | 0.25 | 0.03 | 4 | 1 | PHX | 2010 | MSW | 1 | 0 |
| GCGS0189 | 0.25 | 0.06 | 16 | 1 | SDG | 2010 | MSM | 1 | 0 |
| GCGS0205 | 0.25 | 0.125 | 16 | 1 | SDG | 2010 | MSM | 1 | 0 |
| GCGS0227 | 0.25 | 0.06 | 16 | 1 | SDG | 2010 | MSM | 1 | 0 |
| GCGS0103 | 0.25 | 0.03 | 16 | 0.5 | DEN | 2010 | MSM | 1 | 1 |
| GCGS0105 | 0.25 | 0.06 | 16 | 0.5 | DEN | 2010 | MSW | 1 | 0 |
| GCGS0107 | 0.25 | 0.125 | 16 | 0.5 | GRB | 2010 | MSMW | 1 | 0 |
| GCGS0109 | 0.25 | 0.06 | 32 | 0.5 | HON | 2010 | MSM | 1 | 0 |
| GCGS0111 | 0.25 | 0.25 | 32 | 0.5 | HON | 2010 | MSM | 1 | 0 |
| GCGS0113 | 0.25 | 0.03 | 32 | 0.5 | HON | 2010 | MSMW | 1 | 0 |
| GCGS0123 | 0.25 | 0.125 | 16 | 0.5 | LAX | 2010 | MSMW | 1 | 0 |
| GCGS0125 | 0.25 | 0.06 | 16 | 0.5 | LAX | 2010 | MSM | 1 | 0 |
| GCGS0129 | 0.25 | 0.03 | 32 | 0.5 | LAX | 2010 | MSM | 1 | 0 |
| GCGS0131 | 0.25 | 0.03 | 32 | 0.5 | LAX | 2010 | MSM | 1 | 0 |
| GCGS0133 | 0.25 | 0.03 | 32 | 0.5 | LAX | 2010 | MSM | 1 | 0 |
| GCGS0137 | 0.25 | 0.06 | 32 | 0.5 | LAX | 2010 | MSM | 1 | 0 |
| GCGS0141 | 0.25 | 0.06 | 16 | 0.5 | LVG | 2010 | MSW | 1 | 0 |
| GCGS0143 | 0.25 | 0.125 | 16 | 0.5 | LVG | 2010 | MSW | 1 | 0 |
| GCGS0149 | 0.25 | 0.03 | 0.015 | 0.5 | LVG | 2010 | MSW | 1 | 0 |
| GCGS0153 | 0.25 | 0.03 | 0.015 | 0.5 | LVG | 2010 | MSW | 1 | 0 |
| GCGS0159 | 0.25 | 0.06 | 8 | 0.5 | ORA | 2010 | MSM | 1 | 0 |
| GCGS0161 | 0.25 | 0.03 | 0.015 | 0.5 | ORA | 2010 | MSM | 1 | 0 |
| GCGS0163 | 0.25 | 0.25 | 16 | 0.5 | PHI | 2010 | MSM | 1 | 0 |
| GCGS0167 | 0.25 | 0.03 | 0.015 | 0.5 | PHX | 2010 | MSM | 1 | 0 |
| GCGS0169 | 0.25 | 0.03 | 0.015 | 0.5 | PHX | 2010 | MSM | 1 | 0 |
| GCGS0171 | 0.25 | 0.06 | 0.015 | 0.5 | PHX | 2010 | MSM | 1 | 0 |
| GCGS0175 | 0.25 | 0.06 | 0.015 | 0.5 | PHX | 2010 | MSMW | 1 | 0 |
| GCGS0191 | 0.25 | 0.03 | 0.015 | 0.5 | SDG | 2010 | MSM | 1 | 0 |
| GCGS0193 | 0.25 | 0.03 | 0.015 | 0.5 | SDG | 2010 | MSW | 1 | 0 |
| GCGS0195 | 0.25 | 0.06 | 16 | 0.5 | SDG | 2010 | MSMW | 1 | 0 |
| GCGS0197 | 0.25 | 0.032 | 0.004 | 0.5 | SDG | 2010 | MSM | 1 | 0 |
| GCGS0199 | 0.25 | 0.063 | 0.004 | 0.5 | SDG | 2010 | MSW | 1 | 0 |
| GCGS0201 | 0.25 | 0.063 | 0.004 | 0.5 | SDG | 2010 | MSM | 1 | 0 |
| GCGS0203 | 0.25 | 0.06 | 0.015 | 0.5 | SDG | 2010 | MSM | 1 | 0 |
| GCGS0207 | 0.25 | 0.06 | 0.015 | 0.5 | SDG | 2010 | MSM | 1 | 0 |
| GCGS0209 | 0.25 | 0.03 | 0.015 | 0.5 | SDG | 2010 | MSM | 1 | 0 |
| GCGS0217 | 0.25 | 0.03 | 0.015 | 0.5 | SDG | 2010 | MSM | 1 | 0 |
| GCGS0219 | 0.25 | 0.03 | 0.015 | 0.5 | SDG | 2010 | MSM | 1 | 0 |
| GCGS0221 | 0.25 | 0.03 | 0.015 | 0.5 | SDG | 2010 | MSW | 1 | 0 |
| GCGS0223 | 0.25 | 0.03 | 0.015 | 0.5 | SDG | 2010 | MSM | 1 | 0 |
| GCGS0225 | 0.25 | 0.03 | 0.015 | 0.5 | SDG | 2010 | MSM | 1 | 0 |
| GCGS0229 | 0.25 | 0.06 | 16 | 0.5 | SDG | 2010 | MSM | 1 | 0 |
| GCGS0233 | 0.25 | 0.06 | 32 | 0.5 | SEA | 2010 | MSM | 1 | 0 |
| GCGS0117 | 0.25 | 0.06 | 16 | 0.25 | HON | 2010 | MSW | 1 | 0 |
| GCGS0119 | 0.25 | 0.06 | 16 | 0.25 | HON | 2010 | MSMW | 1 | 0 |
| GCGS0127 | 0.25 | 0.03 | 8 | 0.25 | LAX | 2010 | MSM | 1 | 0 |
| GCGS0183 | 0.25 | 0.06 | 16 | 0.25 | POR | 2010 | MSM | 1 | 1 |
| GCGS0185 | 0.25 | 0.06 | 16 | 0.25 | POR | 2010 | MSM | 1 | 0 |
| GCGS0187 | 0.25 | 0.125 | 16 | 0.25 | POR | 2010 | MSM | 1 | 0 |
| GCGS0211 | 0.25 | 0.03 | 0.015 | 0.25 | SDG | 2010 | MSM | 1 | 0 |
| GCGS0215 | 0.25 | 0.03 | 0.015 | 0.25 | SDG | 2010 | MSM | 1 | 0 |
| GCGS0231 | 0.25 | 0.03 | 32 | 0.25 | SEA | 2010 | MSM | 1 | 0 |
| GCGS0235 | 0.25 | 0.06 | 16 | 0.25 | SEA | 2010 | MSM | 1 | 0 |
| GCGS0237 | 0.25 | 0.06 | 16 | 0.25 | SEA | 2010 | MSM | 1 | 0 |
| GCGS0101 | 0.25 | 0.06 | 16 | 0.125 | DEN | 2010 | MSM | 1 | 1 |
| GCGS0102 | 0.125 | 0.063 | 16 | 16 | ALB | 2010 | MSM | 1 | 0 |
| GCGS0104 | 0.125 | 0.063 | 16 | 4 | ALB | 2010 | MSM | 1 | 0 |
| GCGS0124 | 0.125 | 0.063 | 16 | 1 | LAX | 2010 | MSM | 1 | 0 |
| GCGS0836 | 0.125 | 0.03 | 16 | 1 | LVG | 2010 | MSW | 1 | 0 |
| GCGS0841 | 0.125 | 0.06 | 16 | 1 | NYC | 2010 | MSM | 1 | 0 |
| GCGS0848 | 0.125 | 0.06 | 16 | 1 | PHI | 2010 | MSM | 1 | 0 |
| GCGS0820 | 0.125 | 0.015 | 0.015 | 0.5 | ALB | 2010 | MSM | 1 | 0 |
| GCGS0242 | 0.063 | 0.032 | 4 | 0.25 | SFO | 2010 | MSM | 1 | 0 |
| GCGS1016 | 0.5 | 0.125 | 16 | 1 | MIN | 2011 | MSM | 1 | 0 |
| GCGS0889 | 0.25 | 0.125 | 16 | 2 | DEN | 2011 | MSW | 1 | 0 |
| GCGS0873 | 0.25 | 0.06 | 16 | 1 | CHI | 2011 | MSM | 1 | 0 |
| GCGS0874 | 0.25 | 0.125 | 16 | 1 | CHI | 2011 | MSM | 1 | 0 |
| GCGS0888 | 0.25 | 0.06 | 16 | 1 | DEN | 2011 | MSM | 1 | 0 |
| GCGS0913 | 0.25 | 0.125 | 16 | 1 | LVG | 2011 | MSM | 1 | 0 |
| GCGS0924 | 0.25 | 0.06 | 16 | 1 | MIN | 2011 | MSM | 1 | 0 |
| GCGS0925 | 0.25 | 0.06 | 16 | 1 | MIN | 2011 | MSM | 1 | 0 |
| GCGS0936 | 0.25 | 0.06 | 32 | 1 | PHI | 2011 | MSMW | 1 | 0 |
| GCGS0966 | 0.25 | 0.125 | 32 | 1 | SDG | 2011 | MSM | 1 | 0 |
| GCGS0971 | 0.25 | 0.06 | 16 | 1 | SDG | 2011 | MSM | 1 | 0 |
| GCGS0972 | 0.25 | 0.06 | 16 | 1 | SDG | 2011 | MSM | 1 | 0 |
| GCGS0974 | 0.25 | 0.06 | 16 | 1 | SDG | 2011 | MSMW | 1 | 0 |
| GCGS1012 | 0.25 | 0.06 | 16 | 1 | PHX | 2011 | MSMW | 1 | 0 |
| GCGS0767 | 0.25 | 0.125 | 16 | 0.5 | NOR | 2011 | MSM | 1 | 0 |
| GCGS0886 | 0.25 | 0.125 | 16 | 0.5 | DAL | 2011 | MSM | 1 | 0 |
| GCGS0891 | 0.25 | 0.06 | 16 | 0.5 | DEN | 2011 | MSW | 1 | 0 |
| GCGS0892 | 0.25 | 0.06 | 16 | 0.5 | DEN | 2011 | MSM | 1 | 0 |
| GCGS0893 | 0.25 | 0.06 | 16 | 0.5 | DEN | 2011 | MSM | 1 | 0 |
| GCGS0895 | 0.25 | 0.125 | 16 | 0.5 | HON | 2011 | MSM | 1 | 0 |
| GCGS0899 | 0.25 | 0.06 | 16 | 0.5 | HON | 2011 | MSM | 1 | 0 |
| GCGS0901 | 0.25 | 0.06 | 8 | 0.5 | HON | 2011 | MSMW | 1 | 0 |
| GCGS0931 | 0.25 | 0.03 | 0.015 | 0.5 | ORA | 2011 | MSMW | 1 | 0 |
| GCGS0937 | 0.25 | 0.125 | 32 | 0.5 | PHI | 2011 | MSM | 1 | 0 |
| GCGS0941 | 0.25 | 0.03 | 0.015 | 0.5 | PHX | 2011 | MSM | 1 | 0 |
| GCGS0958 | 0.25 | 0.125 | 16 | 0.5 | POR | 2011 | MSM | 1 | 0 |
| GCGS0965 | 0.25 | 0.06 | 16 | 0.5 | SDG | 2011 | MSM | 1 | 0 |
| GCGS0967 | 0.25 | 0.03 | 16 | 0.5 | SDG | 2011 | MSM | 1 | 0 |
| GCGS0969 | 0.25 | 0.06 | 16 | 0.5 | SDG | 2011 | MSM | 1 | 0 |
| GCGS0970 | 0.25 | 0.03 | 16 | 0.5 | SDG | 2011 | MSW | 1 | 0 |
| GCGS0973 | 0.25 | 0.06 | 8 | 0.5 | SDG | 2011 | MSM | 1 | 0 |
| GCGS0975 | 0.25 | 0.06 | 32 | 0.5 | SDG | 2011 | MSM | 1 | 0 |
| GCGS0992 | 0.25 | 0.06 | 16 | 0.5 | SEA | 2011 | MSM | 1 | 0 |
| GCGS0999 | 0.25 | 0.06 | 16 | 0.5 | SFO | 2011 | MSM | 1 | 0 |
| GCGS1001 | 0.25 | 0.125 | 32 | 0.5 | SFO | 2011 | MSW | 1 | 0 |
| GCGS1005 | 0.25 | 0.06 | 16 | 0.5 | SFO | 2011 | MSM | 1 | 0 |
| GCGS0871 | 0.25 | 0.06 | 16 | 0.25 | BHM | 2011 | MSW | 1 | 0 |
| GCGS0884 | 0.25 | 0.06 | 8 | 0.25 | DAL | 2011 | MSW | 1 | 0 |
| GCGS0885 | 0.25 | 0.06 | 16 | 0.25 | DAL | 2011 | MSMW | 1 | 0 |
| GCGS0890 | 0.25 | 0.06 | 0.015 | 0.25 | DEN | 2011 | MSM | 1 | 0 |
| GCGS0896 | 0.25 | 0.06 | 16 | 0.25 | HON | 2011 | MSW | 1 | 0 |
| GCGS0897 | 0.25 | 0.06 | 16 | 0.25 | HON | 2011 | MSW | 1 | 0 |
| GCGS0898 | 0.25 | 0.06 | 16 | 0.25 | HON | 2011 | MSW | 1 | 0 |
| GCGS0900 | 0.25 | 0.06 | 16 | 0.25 | HON | 2011 | MSM | 1 | 0 |
| GCGS0902 | 0.25 | 0.06 | 8 | 0.25 | HON | 2011 | MSM | 1 | 0 |
| GCGS0908 | 0.25 | 0.06 | 16 | 0.25 | LAX | 2011 | MSM | 1 | 0 |
| GCGS0909 | 0.25 | 0.06 | 16 | 0.25 | LAX | 2011 | MSM | 1 | 0 |
| GCGS0910 | 0.25 | 0.06 | 16 | 0.25 | LAX | 2011 | MSW | 1 | 0 |
| GCGS0911 | 0.25 | 0.06 | 16 | 0.25 | LAX | 2011 | MSM | 1 | 0 |
| GCGS0945 | 0.25 | 0.06 | 16 | 0.25 | POR | 2011 | MSM | 1 | 0 |
| GCGS0946 | 0.25 | 0.125 | 16 | 0.25 | POR | 2011 | MSMW | 1 | 0 |
| GCGS0947 | 0.25 | 0.06 | 16 | 0.25 | POR | 2011 | MSM | 1 | 0 |
| GCGS0948 | 0.25 | 0.06 | 16 | 0.25 | POR | 2011 | MSM | 1 | 0 |
| GCGS0949 | 0.25 | 0.06 | 8 | 0.25 | POR | 2011 | MSM | 1 | 0 |
| GCGS0950 | 0.25 | 0.125 | 16 | 0.25 | POR | 2011 | MSM | 1 | 0 |
| GCGS0951 | 0.25 | 0.06 | 16 | 0.25 | POR | 2011 | MSM | 1 | 0 |
| GCGS0952 | 0.25 | 0.06 | 16 | 0.25 | POR | 2011 | MSM | 1 | 0 |
| GCGS0953 | 0.25 | 0.06 | 16 | 0.25 | POR | 2011 | MSM | 1 | 0 |
| GCGS0954 | 0.25 | 0.06 | 16 | 0.25 | POR | 2011 | MSMW | 1 | 0 |
| GCGS0955 | 0.25 | 0.06 | 8 | 0.25 | POR | 2011 | MSM | 1 | 0 |
| GCGS0956 | 0.25 | 0.06 | 16 | 0.25 | POR | 2011 | MSM | 1 | 0 |
| GCGS0957 | 0.25 | 0.06 | 16 | 0.25 | POR | 2011 | MSM | 1 | 0 |
| GCGS0964 | 0.25 | 0.03 | 0.015 | 0.25 | SDG | 2011 | MSM | 1 | 0 |
| GCGS0976 | 0.25 | 0.06 | 32 | 0.25 | SDG | 2011 | MSW | 1 | 0 |
| GCGS0993 | 0.25 | 0.06 | 16 | 0.25 | SEA | 2011 | MSM | 1 | 0 |
| GCGS0994 | 0.25 | 0.06 | 16 | 0.25 | SEA | 2011 | MSW | 1 | 0 |
| GCGS0995 | 0.25 | 0.125 | 16 | 0.25 | SEA | 2011 | MSM | 1 | 0 |
| GCGS1000 | 0.25 | 0.06 | 16 | 0.25 | SFO | 2011 | MSM | 1 | 0 |
| GCGS1002 | 0.25 | 0.06 | 8 | 0.25 | SFO | 2011 | MSM | 1 | 0 |
| GCGS1003 | 0.25 | 0.06 | 16 | 0.25 | SFO | 2011 | MSW | 1 | 0 |
| GCGS1004 | 0.25 | 0.125 | 16 | 0.25 | SFO | 2011 | MSM | 1 | 0 |
| GCGS0856 | 0.125 | 0.06 | 8 | 8 | POR | 2011 | MSM | 1 | 0 |
| GCGS0842 | 0.125 | 0.125 | 16 | 2 | NYC | 2011 | MSM | 1 | 0 |
| GCGS0821 | 0.125 | 0.03 | 0.015 | 0.5 | ALB | 2011 | MSM | 1 | 0 |
| GCGS0853 | 0.125 | 0.06 | 0.015 | 0.5 | PHX | 2011 | MSM | 1 | 0 |
| GCGS0968 | 0.125 | 0.032 | 4 | 0.5 | SDG | 2011 | MSW | 1 | 0 |
| GCGS0757 | 0.063 | 0.016 | 4 | 1 | MIA | 2011 | MSW | 1 | 0 |
| GCGS0938 | 1 | 0.25 | 16 | 2 | PHI | 2012 | MSM | 1 | 0 |
| GCGS0862 | 0.25 | 0.06 | 8 | 1 | ALB | 2012 | MSM | 1 | 0 |
| GCGS0863 | 0.25 | 0.125 | 8 | 1 | ALB | 2012 | MSM | 1 | 0 |
| GCGS0864 | 0.25 | 0.125 | 8 | 1 | ALB | 2012 | MSM | 1 | 0 |
| GCGS0875 | 0.25 | 0.06 | 16 | 1 | CHI | 2012 | MSW | 1 | 0 |
| GCGS0876 | 0.25 | 0.06 | 16 | 1 | CHI | 2012 | MSM | 1 | 0 |
| GCGS0877 | 0.25 | 0.06 | 16 | 1 | CHI | 2012 | MSW | 1 | 0 |
| GCGS0881 | 0.25 | 0.125 | 16 | 1 | CHI | 2012 | MSM | 1 | 0 |
| GCGS0887 | 0.25 | 0.06 | 16 | 1 | DAL | 2012 | MSM | 1 | 0 |
| GCGS0914 | 0.25 | 0.06 | 16 | 1 | LVG | 2012 | MSW | 1 | 0 |
| GCGS0927 | 0.25 | 0.06 | 16 | 1 | NOR | 2012 | MSW | 1 | 0 |
| GCGS0929 | 0.25 | 0.06 | 8 | 1 | NYC | 2012 | MSM | 1 | 0 |
| GCGS0942 | 0.25 | 0.06 | 16 | 1 | PHX | 2012 | MSM | 1 | 0 |
| GCGS0943 | 0.25 | 0.06 | 16 | 1 | PON | 2012 | MSMW | 1 | 0 |
| GCGS0982 | 0.25 | 0.06 | 16 | 1 | SDG | 2012 | MSW | 1 | 0 |
| GCGS0983 | 0.25 | 0.06 | 16 | 1 | SDG | 2012 | MSM | 1 | 0 |
| GCGS0985 | 0.25 | 0.06 | 16 | 1 | SDG | 2012 | MSM | 1 | 0 |
| GCGS1006 | 0.25 | 0.06 | 16 | 1 | SFO | 2012 | MSM | 1 | 0 |
| GCGS1007 | 0.25 | 0.125 | 16 | 1 | SFO | 2012 | MSM | 1 | 0 |
| GCGS0872 | 0.25 | 0.06 | 16 | 0.5 | BHM | 2012 | MSW | 1 | 0 |
| GCGS0878 | 0.25 | 0.06 | 16 | 0.5 | CHI | 2012 | MSM | 1 | 0 |
| GCGS0879 | 0.25 | 0.06 | 16 | 0.5 | CHI | 2012 | MSM | 1 | 0 |
| GCGS0880 | 0.25 | 0.125 | 16 | 0.5 | CHI | 2012 | MSW | 1 | 0 |
| GCGS0883 | 0.25 | 0.03 | 16 | 0.5 | COL | 2012 | MSM | 1 | 0 |
| GCGS0894 | 0.25 | 0.06 | 16 | 0.5 | DEN | 2012 | MSM | 1 | 0 |
| GCGS0903 | 0.25 | 0.06 | 16 | 0.5 | HON | 2012 | MSM | 1 | 0 |
| GCGS0905 | 0.25 | 0.06 | 16 | 0.5 | LA2 | 2012 | MSMW | 1 | 0 |
| GCGS0906 | 0.25 | 0.06 | 16 | 0.5 | LA2 | 2012 | MSM | 1 | 0 |
| GCGS0912 | 0.25 | 0.125 | 16 | 0.5 | LAX | 2012 | MSMW | 1 | 0 |
| GCGS0915 | 0.25 | 0.125 | 16 | 0.5 | LVG | 2012 | MSM | 1 | 0 |
| GCGS0922 | 0.25 | 0.06 | 8 | 0.5 | MIA | 2012 | MSMW | 1 | 0 |
| GCGS0932 | 0.25 | 0.06 | 32 | 0.5 | ORA | 2012 | MSM | 1 | 0 |
| GCGS0959 | 0.25 | 0.125 | 16 | 0.5 | POR | 2012 | MSM | 1 | 0 |
| GCGS0960 | 0.25 | 0.125 | 8 | 0.5 | POR | 2012 | MSMW | 1 | 0 |
| GCGS0961 | 0.25 | 0.125 | 16 | 0.5 | POR | 2012 | MSM | 1 | 0 |
| GCGS0962 | 0.25 | 0.06 | 16 | 0.5 | POR | 2012 | MSM | 1 | 0 |
| GCGS0977 | 0.25 | 0.06 | 16 | 0.5 | SDG | 2012 | MSM | 1 | 0 |
| GCGS0978 | 0.25 | 0.06 | 16 | 0.5 | SDG | 2012 | MSM | 1 | 0 |
| GCGS0979 | 0.25 | 0.06 | 16 | 0.5 | SDG | 2012 | MSM | 1 | 0 |
| GCGS0980 | 0.25 | 0.06 | 16 | 0.5 | SDG | 2012 | MSM | 1 | 0 |
| GCGS0981 | 0.25 | 0.06 | 16 | 0.5 | SDG | 2012 | MSW | 1 | 0 |
| GCGS0996 | 0.25 | 0.06 | 16 | 0.5 | SEA | 2012 | MSW | 1 | 0 |
| GCGS0997 | 0.25 | 0.06 | 16 | 0.5 | SEA | 2012 | MSM | 1 | 0 |
| GCGS0998 | 0.25 | 0.125 | 16 | 0.5 | SEA | 2012 | MSM | 1 | 0 |
| GCGS0984 | 0.25 | 0.06 | 16 | 0.25 | SDG | 2012 | MSM | 1 | 0 |
| GCGS0986 | 0.25 | 0.06 | 16 | 0.25 | SDG | 2012 | MSM | 1 | 0 |
| GCGS0926 | 0.25 | 0.03 | 16 | 0.125 | MIN | 2012 | MSW | 1 | 0 |
| GCGS0849 | 0.125 | 0.03 | 16 | 4 | PHI | 2012 | MSM | 1 | 0 |
| GCGS0834 | 0.125 | 0.03 | 4 | 2 | LAX | 2012 | MSM | 1 | 0 |
| GCGS0811 | 0.125 | 0.063 | 16 | 1 | SDG | 2012 | MSM | 1 | 0 |
| GCGS0837 | 0.125 | 0.06 | 16 | 1 | LVG | 2012 | MSW | 1 | 0 |
| GCGS0722 | 0.125 | 0.063 | 16 | 0.5 | ATL | 2012 | MSM | 1 | 0 |
| GCGS0740 | 0.125 | 0.032 | 8 | 0.5 | DAL | 2012 | MSW | 1 | 0 |
| GCGS0822 | 0.125 | 0.06 | 8 | 0.5 | ALB | 2012 | MSMW | 1 | 0 |
| GCGS0833 | 0.125 | 0.06 | 16 | 0.5 | LAX | 2012 | MSM | 1 | 0 |
| GCGS0869 | 0.25 | 0.03 | 16 | 1 | BAL | 2013 | MSW | 1 | 0 |
| GCGS0882 | 0.25 | 0.06 | 16 | 1 | CHI | 2013 | MSM | 1 | 0 |
| GCGS0916 | 0.25 | 0.06 | 16 | 1 | LVG | 2013 | MSW | 1 | 0 |
| GCGS0917 | 0.25 | 0.06 | 16 | 1 | LVG | 2013 | MSM | 1 | 0 |
| GCGS0918 | 0.25 | 0.06 | 16 | 1 | LVG | 2013 | MSM | 1 | 0 |
| GCGS0868 | 0.25 | 0.03 | 8 | 0.5 | BAL | 2013 | MSM | 1 | 0 |
| GCGS0904 | 0.25 | 0.06 | 16 | 0.5 | IND | 2013 | MSM | 1 | 0 |
| GCGS0919 | 0.25 | 0.06 | 16 | 0.5 | LVG | 2013 | MSW | 1 | 0 |
| GCGS0923 | 0.25 | 0.125 | 16 | 0.5 | MIA | 2013 | MSMW | 1 | 0 |
| GCGS0935 | 0.25 | 0.03 | 8 | 0.5 | ORA | 2013 | MSM | 1 | 0 |
| GCGS0939 | 0.25 | 0.06 | 16 | 0.5 | PHI | 2013 | MSMW | 1 | 0 |
| GCGS0940 | 0.25 | 0.03 | 8 | 0.5 | PHI | 2013 | MSM | 1 | 0 |
| GCGS0963 | 0.25 | 0.06 | 16 | 0.5 | POR | 2013 | MSM | 1 | 0 |
| GCGS0987 | 0.25 | 0.06 | 16 | 0.5 | SDG | 2013 | MSM | 1 | 0 |
| GCGS0988 | 0.25 | 0.03 | 8 | 0.5 | SDG | 2013 | MSW | 1 | 0 |
| GCGS0989 | 0.25 | 0.03 | 16 | 0.5 | SDG | 2013 | MSW | 1 | 0 |
| GCGS1008 | 0.25 | 0.06 | 16 | 0.5 | SFO | 2013 | MSM | 1 | 0 |
| GCGS1009 | 0.25 | 0.125 | 16 | 0.5 | SFO | 2013 | MSM | 1 | 0 |
| GCGS1010 | 0.25 | 0.06 | 16 | 0.5 | SFO | 2013 | MSM | 1 | 0 |
| GCGS1011 | 0.25 | 0.06 | 16 | 0.5 | SFO | 2013 | MSW | 1 | 0 |
| GCGS0838 | 0.125 | 0.03 | 16 | 16 | LVG | 2013 | MSW | 1 | 0 |
| GCGS0759 | 0.125 | 0.063 | 16 | 8 | MIA | 2013 | MSW | 1 | 0 |
| GCGS0854 | 0.125 | 0.03 | 16 | 1 | PHX | 2013 | MSW | 1 | 0 |
| GCGS0780 | 0.125 | 0.032 | 16 | 0.5 | ORA | 2013 | MSM | 1 | 0 |
| GCGS0990 | 0.125 | 0.032 | 16 | 0.5 | SDG | 2013 | MSM | 1 | 0 |
| GCGS0991 | 0.125 | 0.032 | 16 | 0.5 | SDG | 2013 | MSW | 1 | 0 |
| GCGS0934 | 0.063 | 0.032 | 2 | 0.063 | ORA | 2013 | MSW | 1 | 0 |
| GCGS0781 | 0.032 | 0.016 | 4 | 0.063 | ORA | 2013 | MSMW | 1 | 0 |
| GCGS0870 | 1 | 0.125 | 0.25 | 2 | BHM | 2000 | MSW | 0 | 0 |
| GCGS0795 | 0.06 | 0.015 | 0.008 | 2 | SDG | 2000 | MSM | 0 | 0 |
| GCGS0718 | 0.06 | 0.06 | 0.03 | 1 | ATL | 2000 | MSM | 0 | 0 |
| GCGS0735 | 0.06 | 0.03 | 0.015 | 0.5 | CIN | 2000 | MSW | 0 | 0 |
| GCGS0734 | 0.06 | 0.03 | 0.008 | 0.25 | CIN | 2000 | MSW | 0 | 0 |
| GCGS0812 | 0.06 | 0.03 | 0.015 | 0.25 | SFO | 2000 | MSW | 0 | 0 |
| GCGS1097 | 0.06 | 0.03 | 0.06 | 0.25 | DEN | 2000 | MSMW | 0 | 0 |
| GCGS0525 | 0.03 | 0.004 | 0.004 | 2 | ATL | 2000 | MSW | 0 | 0 |
| GCGS0595 | 0.03 | 0.015 | 0.004 | 2 | LBC | 2000 | - | 0 | 0 |
| GCGS0621 | 0.03 | 0.015 | 0.004 | 2 | ORA | 2000 | MSMW | 0 | 0 |
| GCGS0703 | 0.03 | 0.008 | 0.008 | 2 | SEA | 2000 | MSW | 0 | 0 |
| GCGS0569 | 0.03 | 0.03 | 0.015 | 1 | CLE | 2000 | MSW | 0 | 0 |
| GCGS0524 | 0.03 | 0.008 | 0.004 | 0.25 | ALB | 2000 | MSW | 0 | 0 |
| GCGS0536 | 0.03 | 0.015 | 0.008 | 0.25 | BAL | 2000 | MSW | 0 | 0 |
| GCGS0546 | 0.03 | 0.008 | 0.004 | 0.25 | BHM | 2000 | MSW | 0 | 0 |
| GCGS0702 | 0.03 | 0.015 | 0.015 | 0.25 | SEA | 2000 | MSM | 0 | 0 |
| GCGS0709 | 0.03 | 0.015 | 0.06 | 0.25 | SFO | 2000 | MSMW | 0 | 0 |
| GCGS0503 | 0.015 | 0.008 | 0.008 | 2 | SFO | 2000 | MSM | 0 | 0 |
| GCGS0322 | 0.015 | 0.008 | 0.004 | 1 | CHI | 2000 | MSW | 0 | 0 |
| GCGS0351 | 0.015 | 0.004 | 0.015 | 1 | DAL | 2000 | MSW | 0 | 0 |
| GCGS0374 | 0.015 | 0.004 | 0.004 | 1 | KCY | 2000 | MSW | 0 | 0 |
| GCGS0500 | 0.015 | 0.008 | 0.008 | 1 | SFO | 2000 | MSM | 0 | 0 |
| GCGS0502 | 0.015 | 0.015 | 0.03 | 1 | SFO | 2000 | MSM | 0 | 0 |
| GCGS0309 | 0.015 | 0.015 | 0.015 | 0.5 | BAL | 2000 | MSM | 0 | 0 |
| GCGS0366 | 0.015 | 0.015 | 0.015 | 0.5 | FBG | 2000 | - | 0 | 0 |
| GCGS0364 | 0.015 | 0.015 | 0.008 | 0.25 | FBG | 2000 | - | 0 | 0 |
| GCGS0365 | 0.015 | 0.015 | 0.008 | 0.25 | FBG | 2000 | - | 0 | 0 |
| GCGS0407 | 0.015 | 0.015 | 0.008 | 0.25 | MIN | 2000 | - | 0 | 0 |
| GCGS0501 | 0.015 | 0.008 | 0.008 | 0.25 | SFO | 2000 | MSW | 0 | 0 |
| GCGS0273 | 0.008 | 0.004 | 0.008 | 2 | FBG | 2000 | - | 0 | 0 |
| GCGS0275 | 0.008 | 0.004 | 0.004 | 2 | KCY | 2000 | MSW | 0 | 0 |
| GCGS0276 | 0.008 | 0.004 | 0.004 | 1 | KCY | 2000 | MSW | 0 | 0 |
| GCGS0293 | 0.008 | 0.004 | 0.004 | 1 | ORA | 2000 | MSM | 0 | 0 |
| GCGS0270 | 0.008 | 0.008 | 0.015 | 0.25 | CLE | 2000 | MSW | 0 | 0 |
| GCGS0286 | 0.008 | 0.008 | 0.008 | 0.25 | MIN | 2000 | - | 0 | 0 |
| GCGS0249 | 0.004 | 0.004 | 0.008 | 4 | KCY | 2000 | MSW | 0 | 0 |
| GCGS0251 | 0.004 | 0.004 | 0.008 | 0.25 | MIN | 2000 | - | 0 | 0 |
| GCGS0829 | 0.125 | 0.03 | 0.008 | 0.25 | CIN | 2001 | MSW | 0 | 0 |
| GCGS0830 | 0.125 | 0.06 | 0.015 | 0.25 | CIN | 2001 | MSW | 0 | 0 |
| GCGS0831 | 0.125 | 0.06 | 0.015 | 0.25 | CIN | 2001 | MSW | 0 | 0 |
| GCGS0845 | 0.125 | 0.015 | 0.015 | 0.25 | PHI | 2001 | MSW | 0 | 0 |
| GCGS0855 | 0.125 | 0.03 | 0.015 | 0.25 | POR | 2001 | MSM | 0 | 0 |
| GCGS0741 | 0.06 | 0.03 | 0.004 | 2 | DEN | 2001 | MSMW | 0 | 0 |
| GCGS0813 | 0.06 | 0.03 | 0.008 | 2 | SFO | 2001 | MSM | 0 | 0 |
| GCGS0723 | 0.06 | 0.03 | 0.015 | 1 | BAL | 2001 | MSW | 0 | 0 |
| GCGS0726 | 0.06 | 0.03 | 0.004 | 0.5 | BHM | 2001 | MSW | 0 | 0 |
| GCGS0736 | 0.06 | 0.03 | 0.015 | 0.25 | CIN | 2001 | MSW | 0 | 0 |
| GCGS0865 | 0.032 | 0.032 | 0.016 | 1 | BAL | 2001 | MSW | 0 | 0 |
| GCGS0526 | 0.03 | 0.008 | 0.004 | 1 | ATL | 2001 | - | 0 | 0 |
| GCGS0576 | 0.03 | 0.015 | 0.008 | 1 | DEN | 2001 | MSMW | 0 | 0 |
| GCGS0622 | 0.03 | 0.015 | 0.004 | 1 | ORA | 2001 | MSW | 0 | 0 |
| GCGS0565 | 0.03 | 0.03 | 0.008 | 0.5 | CIN | 2001 | - | 0 | 0 |
| GCGS1096 | 0.03 | 0.008 | 0.008 | 0.25 | LBC | 2001 | MSMW | 0 | 0 |
| GCGS1103 | 0.03 | 0.015 | 0.004 | 0.25 | SDG | 2001 | MSW | 0 | 1 |
| GCGS0356 | 0.015 | 0.008 | 0.004 | 4 | DEN | 2001 | MSMW | 0 | 0 |
| GCGS0367 | 0.015 | 0.008 | 0.015 | 4 | HON | 2001 | MSW | 0 | 0 |
| GCGS0304 | 0.015 | 0.002 | 0.004 | 2 | ATL | 2001 | MSW | 0 | 1 |
| GCGS0305 | 0.015 | 0.004 | 0.004 | 2 | ATL | 2001 | MSW | 0 | 0 |
| GCGS0357 | 0.015 | 0.004 | 0.004 | 1 | DEN | 2001 | MSW | 0 | 0 |
| GCGS0439 | 0.015 | 0.008 | 0.004 | 0.5 | PHI | 2001 | MSW | 0 | 0 |
| GCGS0306 | 0.015 | 0.015 | 0.008 | 0.25 | ATL | 2001 | MSMW | 0 | 1 |
| GCGS0317 | 0.015 | 0.008 | 0.008 | 0.25 | BHM | 2001 | MSW | 0 | 0 |
| GCGS0255 | 0.008 | 0.004 | 0.004 | 2 | ATL | 2001 | MSW | 0 | 0 |
| GCGS0291 | 0.008 | 0.004 | 0.004 | 2 | NOR | 2001 | MSW | 0 | 0 |
| GCGS0245 | 0.002 | 0.004 | 0.004 | 0.25 | ORA | 2001 | MSMW | 0 | 0 |
| GCGS0824 | 0.125 | 0.06 | 0.015 | 1 | BAL | 2002 | MSW | 0 | 0 |
| GCGS0846 | 0.125 | 0.03 | 0.015 | 1 | PHI | 2002 | MSW | 0 | 0 |
| GCGS0839 | 0.125 | 0.015 | 0.002 | 0.25 | MIA | 2002 | MSM | 0 | 0 |
| GCGS0719 | 0.06 | 0.03 | 0.004 | 4 | ATL | 2002 | MSM | 0 | 0 |
| GCGS0724 | 0.06 | 0.06 | 0.015 | 1 | BAL | 2002 | MSW | 0 | 0 |
| GCGS0771 | 0.06 | 0.015 | 0.004 | 1 | ORA | 2002 | MSW | 0 | 0 |
| GCGS0727 | 0.06 | 0.015 | 0.004 | 0.5 | BHM | 2002 | MSW | 0 | 0 |
| GCGS0737 | 0.06 | 0.03 | 0.015 | 0.5 | CIN | 2002 | MSW | 0 | 0 |
| GCGS0766 | 0.06 | 0.008 | 0.004 | 0.25 | NOR | 2002 | MSW | 0 | 0 |
| GCGS0782 | 0.06 | 0.008 | 0.004 | 0.25 | PHI | 2002 | MSW | 0 | 1 |
| GCGS0796 | 0.06 | 0.06 | 8 | 0.25 | SDG | 2002 | MSM | 0 | 0 |
| GCGS0797 | 0.06 | 0.03 | 8 | 0.25 | SDG | 2002 | MSM | 0 | 0 |
| GCGS0798 | 0.06 | 0.06 | 16 | 0.25 | SDG | 2002 | MSM | 0 | 0 |
| GCGS0866 | 0.032 | 0.032 | 0.0169 | 1 | BAL | 2002 | MSW | 0 | 0 |
| GCGS0867 | 0.032 | 0.032 | 0.016 | 1 | BAL | 2002 | MSW | 0 | 0 |
| GCGS0704 | 0.03 | 0.015 | 0.008 | 4 | SEA | 2002 | MSW | 0 | 0 |
| GCGS0577 | 0.03 | 0.008 | 0.008 | 2 | DEN | 2002 | MSW | 0 | 0 |
| GCGS0578 | 0.03 | 0.008 | 0.008 | 2 | DEN | 2002 | MSMW | 0 | 0 |
| GCGS0579 | 0.03 | 0.008 | 0.008 | 2 | DEN | 2002 | MSW | 0 | 0 |
| GCGS0581 | 0.03 | 0.008 | 0.008 | 2 | DEN | 2002 | MSMW | 0 | 0 |
| GCGS0623 | 0.03 | 0.004 | 0.004 | 2 | ORA | 2002 | MSW | 0 | 0 |
| GCGS0580 | 0.03 | 0.015 | 0.008 | 1 | DEN | 2002 | MSMW | 0 | 0 |
| GCGS0612 | 0.03 | 0.015 | 0.015 | 1 | MIN | 2002 | MSW | 0 | 0 |
| GCGS0646 | 0.03 | 0.015 | 0.015 | 1 | PHI | 2002 | MSMW | 0 | 0 |
| GCGS0662 | 0.03 | 0.004 | 0.008 | 1 | PHX | 2002 | MSW | 0 | 0 |
| GCGS0663 | 0.03 | 0.015 | 0.008 | 1 | PHX | 2002 | MSM | 0 | 0 |
| GCGS0613 | 0.03 | 0.03 | 0.004 | 0.5 | MIN | 2002 | MSW | 0 | 0 |
| GCGS0682 | 0.03 | 0.03 | 0.015 | 0.5 | SDG | 2002 | MSM | 0 | 0 |
| GCGS0537 | 0.03 | 0.015 | 0.015 | 0.25 | BAL | 2002 | MSW | 0 | 0 |
| GCGS0566 | 0.03 | 0.03 | 0.008 | 0.25 | CIN | 2002 | MSW | 0 | 0 |
| GCGS0605 | 0.03 | 0.03 | 0.015 | 0.25 | MIA | 2002 | MSW | 0 | 0 |
| GCGS0624 | 0.03 | 0.015 | 2 | 0.25 | ORA | 2002 | MSW | 0 | 1 |
| GCGS0625 | 0.03 | 0.015 | 2 | 0.25 | ORA | 2002 | MSM | 0 | 0 |
| GCGS0647 | 0.03 | 0.015 | 0.008 | 0.25 | PHI | 2002 | MSW | 0 | 1 |
| GCGS0673 | 0.03 | 0.015 | 4 | 0.25 | POR | 2002 | MSM | 0 | 0 |
| GCGS0683 | 0.03 | 0.06 | 8 | 0.25 | SDG | 2002 | MSM | 0 | 0 |
| GCGS0684 | 0.03 | 0.03 | 8 | 0.25 | SDG | 2002 | MSM | 0 | 0 |
| GCGS0307 | 0.015 | 0.008 | 0.004 | 2 | ATL | 2002 | MSW | 0 | 0 |
| GCGS0408 | 0.015 | 0.015 | 0.008 | 1 | MIN | 2002 | MSW | 0 | 0 |
| GCGS0409 | 0.015 | 0.015 | 0.004 | 1 | MIN | 2002 | MSW | 0 | 0 |
| GCGS0441 | 0.015 | 0.004 | 0.004 | 1 | PHI | 2002 | MSW | 0 | 0 |
| GCGS0521 | 0.015 | 0.004 | 0.004 | 1 | STL | 2002 | MSM | 0 | 0 |
| GCGS0358 | 0.015 | 0.008 | 0.008 | 0.25 | DEN | 2002 | MSMW | 0 | 1 |
| GCGS0385 | 0.015 | 0.015 | 8 | 0.25 | LBC | 2002 | MSW | 0 | 0 |
| GCGS0440 | 0.015 | 0.004 | 0.002 | 0.25 | PHI | 2002 | MSW | 0 | 0 |
| GCGS0462 | 0.015 | 0.008 | 0.004 | 0.25 | PHX | 2002 | MSW | 0 | 0 |
| GCGS0498 | 0.015 | 0.008 | 8 | 0.25 | SEA | 2002 | MSM | 0 | 0 |
| GCGS0258 | 0.008 | 0.002 | 0.004 | 4 | BHM | 2002 | MSW | 0 | 0 |
| GCGS0295 | 0.008 | 0.004 | 0.008 | 4 | SDG | 2002 | MSM | 0 | 0 |
| GCGS0302 | 0.008 | 0.004 | 0.004 | 4 | STL | 2002 | MSW | 0 | 0 |
| GCGS0277 | 0.008 | 0.008 | 0.015 | 0.25 | LBC | 2002 | MSW | 0 | 0 |
| GCGS0287 | 0.008 | 0.008 | 0.008 | 0.25 | MIN | 2002 | MSW | 0 | 0 |
| GCGS0294 | 0.008 | 0.002 | 0.004 | 0.25 | POR | 2002 | MSW | 0 | 0 |
| GCGS0819 | 0.125 | 0.008 | 0.06 | 0.5 | ALB | 2003 | MSW | 0 | 0 |
| GCGS0627 | 0.063 | 0.25 | 32 | 1 | ORA | 2003 | MSM | 0 | 0 |
| GCGS0720 | 0.06 | 0.015 | 0.004 | 4 | ATL | 2003 | MSW | 0 | 0 |
| GCGS0721 | 0.06 | 0.015 | 0.004 | 4 | ATL | 2003 | MSW | 0 | 0 |
| GCGS0772 | 0.06 | 0.03 | 0.004 | 2 | ORA | 2003 | MSW | 0 | 0 |
| GCGS0773 | 0.06 | 0.06 | 4 | 0.25 | ORA | 2003 | MSM | 0 | 0 |
| GCGS0774 | 0.06 | 0.015 | 4 | 0.25 | ORA | 2003 | MSM | 0 | 0 |
| GCGS0799 | 0.06 | 0.015 | 8 | 0.25 | SDG | 2003 | MSM | 0 | 0 |
| GCGS0527 | 0.03 | 0.015 | 0.004 | 4 | ATL | 2003 | MSW | 0 | 0 |
| GCGS0529 | 0.03 | 0.015 | 0.004 | 4 | ATL | 2003 | MSW | 0 | 0 |
| GCGS0649 | 0.03 | 0.015 | 0.004 | 4 | PHI | 2003 | MSW | 0 | 0 |
| GCGS0528 | 0.03 | 0.015 | 0.004 | 2 | ATL | 2003 | MSW | 0 | 0 |
| GCGS0705 | 0.03 | 0.004 | 0.004 | 2 | SEA | 2003 | MSM | 0 | 0 |
| GCGS0573 | 0.03 | 0.015 | 0.03 | 1 | DAL | 2003 | MSW | 0 | 0 |
| GCGS0588 | 0.03 | 0.03 | 2 | 0.5 | LAX | 2003 | MSM | 0 | 0 |
| GCGS0648 | 0.03 | 0.015 | 0.015 | 0.5 | PHI | 2003 | MSMW | 0 | 0 |
| GCGS0626 | 0.03 | 0.015 | 4 | 0.25 | ORA | 2003 | MSM | 0 | 0 |
| GCGS0628 | 0.03 | 0.03 | 4 | 0.25 | ORA | 2003 | MSW | 0 | 0 |
| GCGS0629 | 0.03 | 0.03 | 4 | 0.25 | ORA | 2003 | MSM | 0 | 0 |
| GCGS0710 | 0.03 | 0.015 | 16 | 0.25 | SFO | 2003 | MSM | 0 | 0 |
| GCGS0711 | 0.03 | 0.015 | 4 | 0.25 | SFO | 2003 | MSM | 0 | 0 |
| GCGS0519 | 0.015 | 0.008 | 0.002 | 4 | SLC | 2003 | MSW | 0 | 0 |
| GCGS0520 | 0.015 | 0.002 | 0.002 | 4 | SLC | 2003 | MSM | 0 | 0 |
| GCGS0386 | 0.015 | 0.008 | 0.004 | 2 | LVG | 2003 | MSM | 0 | 0 |
| GCGS0505 | 0.015 | 0.008 | 0.008 | 2 | SFO | 2003 | MSMW | 0 | 0 |
| GCGS0410 | 0.015 | 0.008 | 2 | 0.5 | MIN | 2003 | MSM | 0 | 0 |
| GCGS0442 | 0.015 | 0.015 | 0.008 | 0.25 | PHI | 2003 | MSW | 0 | 1 |
| GCGS0504 | 0.015 | 0.008 | 16 | 0.25 | SFO | 2003 | MSM | 0 | 0 |
| GCGS0259 | 0.008 | 0.004 | 0.004 | 2 | BHM | 2003 | MSW | 0 | 0 |
| GCGS0260 | 0.008 | 0.004 | 0.004 | 2 | BHM | 2003 | MSW | 0 | 0 |
| GCGS0274 | 0.008 | 0.004 | 0.004 | 2 | GRB | 2003 | MSW | 0 | 0 |
| GCGS0301 | 0.008 | 0.008 | 0.002 | 2 | SLC | 2003 | - | 0 | 0 |
| GCGS0268 | 0.008 | 0.004 | 0.004 | 1 | CIN | 2003 | MSW | 0 | 0 |
| GCGS0256 | 0.008 | 0.004 | 0.004 | 0.5 | ATL | 2003 | MSW | 0 | 0 |
| GCGS0278 | 0.008 | 0.004 | 2 | 0.25 | LVG | 2003 | MSM | 0 | 0 |
| GCGS0279 | 0.008 | 0.008 | 0.125 | 0.25 | LVG | 2003 | MSW | 0 | 1 |
| GCGS0825 | 0.125 | 0.06 | 0.03 | 1 | BAL | 2004 | MSW | 0 | 0 |
| GCGS0826 | 0.125 | 0.06 | 0.03 | 1 | BAL | 2004 | MSW | 0 | 0 |
| GCGS0840 | 0.125 | 0.06 | 4 | 0.25 | MIA | 2004 | MSMW | 0 | 0 |
| GCGS0843 | 0.125 | 0.015 | 0.004 | 0.25 | OKC | 2004 | MSW | 0 | 0 |
| GCGS0729 | 0.06 | 0.03 | 0.004 | 1 | CHI | 2004 | MSW | 0 | 0 |
| GCGS0738 | 0.06 | 0.03 | 0.015 | 1 | CIN | 2004 | MSW | 0 | 0 |
| GCGS0760 | 0.06 | 0.03 | 0.008 | 1 | MIN | 2004 | MSW | 0 | 0 |
| GCGS0728 | 0.06 | 0.015 | 0.004 | 0.5 | BHM | 2004 | MSW | 0 | 0 |
| GCGS0761 | 0.06 | 0.03 | 0.008 | 0.5 | MIN | 2004 | MSW | 0 | 0 |
| GCGS1102 | 0.06 | 0.03 | 16 | 0.5 | MIA | 2004 | - | 0 | 0 |
| GCGS0775 | 0.06 | 0.015 | 4 | 0.25 | ORA | 2004 | MSM | 0 | 0 |
| GCGS0783 | 0.06 | 0.015 | 2 | 0.25 | PHI | 2004 | MSM | 0 | 0 |
| GCGS0784 | 0.06 | 0.03 | 0.008 | 0.25 | PHI | 2004 | MSW | 0 | 1 |
| GCGS1099 | 0.06 | 0.03 | 16 | 0.25 | MIA | 2004 | MSW | 0 | 0 |
| GCGS0589 | 0.03 | 0.015 | 8 | 8 | LAX | 2004 | MSW | 0 | 0 |
| GCGS0531 | 0.03 | 0.015 | 0.004 | 4 | ATL | 2004 | MSW | 0 | 0 |
| GCGS0532 | 0.03 | 0.015 | 0.004 | 4 | ATL | 2004 | MSM | 0 | 0 |
| GCGS0533 | 0.03 | 0.015 | 0.004 | 4 | ATL | 2004 | MSW | 0 | 0 |
| GCGS0534 | 0.03 | 0.015 | 0.004 | 4 | ATL | 2004 | MSW | 0 | 0 |
| GCGS0535 | 0.03 | 0.008 | 0.004 | 4 | ATL | 2004 | MSMW | 0 | 0 |
| GCGS0685 | 0.03 | 0.015 | 0.008 | 4 | SDG | 2004 | MSMW | 0 | 0 |
| GCGS0555 | 0.03 | 0.015 | 0.015 | 2 | CHI | 2004 | MSM | 0 | 0 |
| GCGS0687 | 0.03 | 0.015 | 0.008 | 2 | SDG | 2004 | MSM | 0 | 0 |
| GCGS0530 | 0.03 | 0.015 | 0.008 | 1 | ATL | 2004 | MSW | 0 | 0 |
| GCGS0539 | 0.03 | 0.03 | 0.015 | 1 | BAL | 2004 | MSW | 0 | 0 |
| GCGS0570 | 0.03 | 0.03 | 0.015 | 1 | CLE | 2004 | MSM | 0 | 0 |
| GCGS0597 | 0.03 | 0.03 | 16 | 1 | LVG | 2004 | MSM | 0 | 0 |
| GCGS0598 | 0.03 | 0.03 | 0.03 | 1 | LVG | 2004 | MSMW | 0 | 0 |
| GCGS0600 | 0.03 | 0.015 | 0.008 | 1 | LVG | 2004 | MSMW | 0 | 0 |
| GCGS0607 | 0.03 | 0.03 | 0.015 | 1 | MIA | 2004 | MSW | 0 | 0 |
| GCGS0652 | 0.03 | 0.015 | 8 | 1 | PHI | 2004 | MSM | 0 | 0 |
| GCGS0538 | 0.03 | 0.008 | 0.004 | 0.5 | BAL | 2004 | MSW | 0 | 0 |
| GCGS0540 | 0.03 | 0.008 | 0.008 | 0.5 | BAL | 2004 | MSM | 0 | 0 |
| GCGS0541 | 0.03 | 0.008 | 0.004 | 0.5 | BAL | 2004 | MSW | 0 | 0 |
| GCGS0547 | 0.03 | 0.015 | 0.008 | 0.5 | BHM | 2004 | MSW | 0 | 0 |
| GCGS0630 | 0.03 | 0.015 | 0.008 | 0.5 | ORA | 2004 | MSM | 0 | 0 |
| GCGS0686 | 0.03 | 0.03 | 8 | 0.5 | SDG | 2004 | MSM | 0 | 0 |
| GCGS0599 | 0.03 | 0.015 | 0.004 | 0.25 | LVG | 2004 | MSW | 0 | 0 |
| GCGS0606 | 0.03 | 0.03 | 4 | 0.25 | MIA | 2004 | MSM | 0 | 0 |
| GCGS0631 | 0.03 | 0.03 | 8 | 0.25 | ORA | 2004 | MSW | 0 | 0 |
| GCGS0650 | 0.03 | 0.015 | 4 | 0.25 | PHI | 2004 | MSM | 0 | 0 |
| GCGS0651 | 0.03 | 0.015 | 0.008 | 0.25 | PHI | 2004 | MSW | 0 | 0 |
| GCGS0664 | 0.03 | 0.03 | 8 | 0.25 | PHX | 2004 | MSW | 0 | 0 |
| GCGS0688 | 0.03 | 0.015 | 8 | 0.25 | SDG | 2004 | MSM | 0 | 0 |
| GCGS0689 | 0.03 | 0.03 | 8 | 0.25 | SDG | 2004 | MSM | 0 | 0 |
| GCGS1098 | 0.03 | 0.015 | 2 | 0.25 | LAX | 2004 | MSW | 0 | 0 |
| GCGS1104 | 0.03 | 0.03 | 8 | 0.25 | CIN | 2004 | MSM | 0 | 0 |
| GCGS0308 | 0.015 | 0.015 | 0.004 | 4 | ATL | 2004 | MSW | 0 | 0 |
| GCGS0490 | 0.015 | 0.008 | 4 | 4 | SDG | 2004 | MSM | 0 | 0 |
| GCGS0492 | 0.015 | 0.008 | 0.008 | 4 | SDG | 2004 | MSW | 0 | 0 |
| GCGS0323 | 0.015 | 0.008 | 0.004 | 2 | CHI | 2004 | MSM | 0 | 0 |
| GCGS0342 | 0.015 | 0.015 | 0.002 | 2 | CIN | 2004 | MSW | 0 | 0 |
| GCGS0387 | 0.015 | 0.008 | 0.004 | 2 | LVG | 2004 | MSM | 0 | 0 |
| GCGS0325 | 0.015 | 0.008 | 0.004 | 1 | CHI | 2004 | MSW | 0 | 0 |
| GCGS0340 | 0.015 | 0.008 | 0.004 | 1 | CIN | 2004 | - | 0 | 0 |
| GCGS0341 | 0.015 | 0.015 | 0.004 | 1 | CIN | 2004 | MSW | 0 | 0 |
| GCGS0343 | 0.015 | 0.015 | 0.004 | 1 | CIN | 2004 | MSW | 0 | 0 |
| GCGS0388 | 0.015 | 0.015 | 16 | 1 | LVG | 2004 | MSW | 0 | 0 |
| GCGS0411 | 0.015 | 0.015 | 4 | 1 | MIN | 2004 | MSMW | 0 | 0 |
| GCGS0412 | 0.015 | 0.015 | 0.015 | 1 | MIN | 2004 | MSM | 0 | 0 |
| GCGS0413 | 0.015 | 0.008 | 4 | 1 | MIN | 2004 | MSM | 0 | 0 |
| GCGS0414 | 0.015 | 0.015 | 4 | 1 | MIN | 2004 | MSM | 0 | 0 |
| GCGS0415 | 0.015 | 0.015 | 4 | 1 | MIN | 2004 | MSMW | 0 | 0 |
| GCGS0434 | 0.015 | 0.008 | 0.004 | 1 | ORA | 2004 | MSM | 0 | 0 |
| GCGS0444 | 0.015 | 0.03 | 16 | 1 | PHI | 2004 | MSW | 0 | 0 |
| GCGS0310 | 0.015 | 0.004 | 0.004 | 0.5 | BAL | 2004 | MSW | 0 | 0 |
| GCGS0324 | 0.015 | 0.015 | 0.004 | 0.5 | CHI | 2004 | MSMW | 0 | 0 |
| GCGS0359 | 0.015 | 0.015 | 0.004 | 0.5 | DEN | 2004 | MSW | 0 | 0 |
| GCGS0362 | 0.015 | 0.015 | 0.015 | 0.5 | DTR | 2004 | MSW | 0 | 0 |
| GCGS0389 | 0.015 | 0.008 | 0.008 | 0.5 | LVG | 2004 | MSM | 0 | 0 |
| GCGS0390 | 0.015 | 0.008 | 0.004 | 0.5 | LVG | 2004 | MSW | 0 | 0 |
| GCGS0443 | 0.015 | 0.004 | 0.004 | 0.5 | PHI | 2004 | MSW | 0 | 0 |
| GCGS0318 | 0.015 | 0.004 | 0.004 | 0.25 | BHM | 2004 | MSW | 0 | 0 |
| GCGS0400 | 0.015 | 0.015 | 0.004 | 0.25 | MIA | 2004 | MSM | 0 | 0 |
| GCGS0480 | 0.015 | 0.015 | 8 | 0.25 | POR | 2004 | MSM | 0 | 0 |
| GCGS0491 | 0.015 | 0.015 | 8 | 0.25 | SDG | 2004 | MSM | 0 | 0 |
| GCGS0499 | 0.015 | 0.015 | 8 | 0.25 | SEA | 2004 | MSM | 0 | 0 |
| GCGS0506 | 0.015 | 0.015 | 8 | 0.25 | SFO | 2004 | MSMW | 0 | 0 |
| GCGS0507 | 0.015 | 0.015 | 8 | 0.25 | SFO | 2004 | MSMW | 0 | 0 |
| GCGS0508 | 0.015 | 0.015 | 4 | 0.25 | SFO | 2004 | MSMW | 0 | 0 |
| GCGS0509 | 0.015 | 0.015 | 8 | 0.25 | SFO | 2004 | MSW | 0 | 1 |
| GCGS1100 | 0.015 | 0.015 | 4 | 0.25 | MIA | 2004 | MSW | 0 | 0 |
| GCGS0264 | 0.008 | 0.008 | 0.004 | 8 | CHI | 2004 | MSW | 0 | 0 |
| GCGS0283 | 0.008 | 0.004 | 0.004 | 8 | LVG | 2004 | MSW | 0 | 0 |
| GCGS0288 | 0.008 | 0.008 | 0.004 | 4 | MIN | 2004 | MSM | 0 | 0 |
| GCGS0280 | 0.008 | 0.004 | 0.004 | 2 | LVG | 2004 | MSW | 0 | 0 |
| GCGS0282 | 0.008 | 0.004 | 0.008 | 2 | LVG | 2004 | MSM | 0 | 0 |
| GCGS0266 | 0.008 | 0.004 | 0.004 | 1 | CHI | 2004 | MSM | 0 | 0 |
| GCGS0269 | 0.008 | 0.004 | 0.002 | 1 | CIN | 2004 | MSW | 0 | 0 |
| GCGS1094 | 0.008 | 0.004 | 2 | 1 | CHI | 2004 | MSM | 0 | 0 |
| GCGS0261 | 0.008 | 0.004 | 0.004 | 0.5 | BHM | 2004 | MSW | 0 | 0 |
| GCGS0265 | 0.008 | 0.004 | 0.008 | 0.5 | CHI | 2004 | MSW | 0 | 0 |
| GCGS0281 | 0.008 | 0.008 | 0.015 | 0.5 | LVG | 2004 | MSW | 0 | 0 |
| GCGS0303 | 0.008 | 0.002 | 0.004 | 0.25 | STL | 2004 | - | 0 | 0 |
| GCGS1101 | 0.008 | 0.008 | 8 | 0.25 | MIN | 2004 | MSM | 0 | 0 |
| GCGS0252 | 0.004 | 0.002 | 0.008 | 2 | MIN | 2004 | MSW | 0 | 0 |
| GCGS0253 | 0.004 | 0.001 | 0.004 | 1 | NOR | 2004 | MSW | 0 | 0 |
| GCGS0248 | 0.004 | 0.002 | 0.004 | 0.5 | CIN | 2004 | MSM | 0 | 0 |
| GCGS0828 | 0.125 | 0.06 | 0.008 | 0.5 | BHM | 2005 | MSW | 0 | 0 |
| GCGS0823 | 0.063 | 0.063 | 0.004 | 2 | ATL | 2005 | MSW | 0 | 0 |
| GCGS0751 | 0.06 | 0.015 | 0.008 | 8 | LAX | 2005 | MSM | 0 | 0 |
| GCGS0786 | 0.06 | 0.015 | 0.015 | 1 | PHI | 2005 | MSW | 0 | 0 |
| GCGS0752 | 0.06 | 0.03 | 16 | 0.5 | LAX | 2005 | MSM | 0 | 0 |
| GCGS0776 | 0.06 | 0.06 | 16 | 0.5 | ORA | 2005 | MSW | 0 | 0 |
| GCGS0785 | 0.06 | 0.015 | 4 | 0.5 | PHI | 2005 | MSM | 0 | 0 |
| GCGS0800 | 0.06 | 0.03 | 16 | 0.5 | SDG | 2005 | MSM | 0 | 0 |
| GCGS0801 | 0.06 | 0.03 | 8 | 0.5 | SDG | 2005 | MSM | 0 | 0 |
| GCGS0802 | 0.06 | 0.03 | 8 | 0.5 | SDG | 2005 | MSM | 0 | 0 |
| GCGS0803 | 0.06 | 0.03 | 8 | 0.5 | SDG | 2005 | MSW | 0 | 0 |
| GCGS0326 | 0.032 | 0.016 | 0.016 | 1 | CHI | 2005 | MSW | 0 | 0 |
| GCGS0907 | 0.032 | 0.063 | 16 | 0.5 | LAX | 2005 | MSM | 0 | 0 |
| GCGS0713 | 0.03 | 0.03 | 0.015 | 16 | SFO | 2005 | MSM | 0 | 0 |
| GCGS0653 | 0.03 | 0.03 | 4 | 4 | PHI | 2005 | MSM | 0 | 0 |
| GCGS0691 | 0.03 | 0.015 | 0.008 | 4 | SDG | 2005 | MSM | 0 | 0 |
| GCGS0556 | 0.03 | 0.015 | 0.008 | 2 | CHI | 2005 | MSM | 0 | 0 |
| GCGS0567 | 0.03 | 0.008 | 0.004 | 2 | CIN | 2005 | MSW | 0 | 0 |
| GCGS0542 | 0.03 | 0.015 | 0.008 | 1 | BAL | 2005 | MSM | 0 | 0 |
| GCGS0568 | 0.03 | 0.06 | 16 | 1 | CIN | 2005 | MSM | 0 | 0 |
| GCGS0601 | 0.03 | 0.015 | 0.004 | 1 | LVG | 2005 | MSW | 0 | 0 |
| GCGS0585 | 0.03 | 0.015 | 16 | 0.5 | HON | 2005 | MSW | 0 | 1 |
| GCGS0590 | 0.03 | 0.03 | 16 | 0.5 | LAX | 2005 | MSMW | 0 | 0 |
| GCGS0608 | 0.03 | 0.015 | 8 | 0.5 | MIA | 2005 | MSM | 0 | 0 |
| GCGS0617 | 0.03 | 0.06 | 4 | 0.5 | NOR | 2005 | MSW | 0 | 0 |
| GCGS0632 | 0.03 | 0.015 | 2 | 0.5 | ORA | 2005 | MSW | 0 | 0 |
| GCGS0633 | 0.03 | 0.03 | 8 | 0.5 | ORA | 2005 | MSMW | 0 | 0 |
| GCGS0690 | 0.03 | 0.03 | 8 | 0.5 | SDG | 2005 | MSM | 0 | 0 |
| GCGS0712 | 0.03 | 0.03 | 8 | 0.5 | SFO | 2005 | MSM | 0 | 0 |
| GCGS0329 | 0.015 | 0.015 | 0.008 | 8 | CHI | 2005 | MSM | 0 | 0 |
| GCGS0380 | 0.015 | 0.008 | 0.008 | 8 | LAX | 2005 | MSW | 0 | 0 |
| GCGS0391 | 0.015 | 0.015 | 0.008 | 8 | LVG | 2005 | MSM | 0 | 0 |
| GCGS0392 | 0.015 | 0.008 | 0.004 | 8 | LVG | 2005 | MSM | 0 | 0 |
| GCGS0416 | 0.015 | 0.008 | 0.008 | 8 | MIN | 2005 | MSM | 0 | 0 |
| GCGS0311 | 0.015 | 0.008 | 0.004 | 4 | BAL | 2005 | MSM | 0 | 0 |
| GCGS0417 | 0.015 | 0.015 | 0.004 | 4 | MIN | 2005 | MSW | 0 | 0 |
| GCGS0327 | 0.015 | 0.015 | 16 | 2 | CHI | 2005 | - | 0 | 0 |
| GCGS0328 | 0.015 | 0.015 | 0.008 | 2 | CHI | 2005 | MSM | 0 | 0 |
| GCGS0420 | 0.015 | 0.008 | 0.008 | 1 | MIN | 2005 | MSM | 0 | 0 |
| GCGS0421 | 0.015 | 0.015 | 16 | 1 | MIN | 2005 | MSM | 0 | 0 |
| GCGS0445 | 0.015 | 0.008 | 0.004 | 1 | PHI | 2005 | MSW | 0 | 0 |
| GCGS0446 | 0.015 | 0.03 | 16 | 1 | PHI | 2005 | MSW | 0 | 0 |
| GCGS0381 | 0.015 | 0.008 | 4 | 0.5 | LAX | 2005 | MSM | 0 | 0 |
| GCGS0418 | 0.015 | 0.008 | 0.004 | 0.5 | MIN | 2005 | MSMW | 0 | 0 |
| GCGS0419 | 0.015 | 0.015 | 16 | 0.5 | MIN | 2005 | MSM | 0 | 0 |
| GCGS0463 | 0.015 | 0.015 | 4 | 0.5 | PHX | 2005 | MSM | 0 | 0 |
| GCGS0493 | 0.015 | 0.008 | 8 | 0.5 | SDG | 2005 | MSM | 0 | 0 |
| GCGS0510 | 0.015 | 0.03 | 16 | 0.5 | SFO | 2005 | MSM | 0 | 0 |
| GCGS0271 | 0.008 | 0.004 | 0.004 | 16 | DAL | 2005 | MSW | 0 | 0 |
| GCGS0272 | 0.008 | 0.004 | 0.004 | 16 | DAL | 2005 | MSM | 0 | 0 |
| GCGS0289 | 0.008 | 0.004 | 0.004 | 8 | MIN | 2005 | MSM | 0 | 0 |
| GCGS0257 | 0.008 | 0.004 | 0.004 | 4 | BAL | 2005 | MSW | 0 | 0 |
| GCGS0267 | 0.008 | 0.008 | 0.004 | 4 | CHI | 2005 | MSM | 0 | 0 |
| GCGS0284 | 0.008 | 0.004 | 0.008 | 4 | LVG | 2005 | MSW | 0 | 0 |
| GCGS0299 | 0.008 | 0.015 | 0.004 | 4 | SFO | 2005 | MSM | 0 | 0 |
| GCGS0296 | 0.008 | 0.008 | 0.008 | 2 | SDG | 2005 | MSW | 0 | 0 |
| GCGS0297 | 0.008 | 0.008 | 0.008 | 2 | SDG | 2005 | MSW | 0 | 0 |
| GCGS0298 | 0.008 | 0.008 | 0.008 | 2 | SDG | 2005 | MSM | 0 | 0 |
| GCGS0262 | 0.008 | 0.004 | 0.004 | 0.5 | BHM | 2005 | MSW | 0 | 0 |
| GCGS0263 | 0.008 | 0.008 | 0.004 | 0.5 | BHM | 2005 | MSW | 0 | 0 |
| GCGS0254 | 0.004 | 0.015 | 0.004 | 4 | SFO | 2005 | MSM | 0 | 0 |
| GCGS0247 | 0.004 | 0.002 | 0.008 | 2 | BHM | 2005 | MSW | 0 | 0 |
| GCGS0250 | 0.004 | 0.002 | 0.008 | 1 | LAX | 2005 | MSW | 0 | 0 |
| GCGS0861 | 0.125 | 0.063 | 16 | 1 | ALB | 2006 | MSMW | 0 | 0 |
| GCGS0777 | 0.06 | 0.03 | 0.015 | 2 | ORA | 2006 | MSMW | 0 | 0 |
| GCGS0753 | 0.06 | 0.03 | 8 | 0.5 | LAX | 2006 | MSM | 0 | 0 |
| GCGS0756 | 0.06 | 0.03 | 16 | 0.5 | LBC | 2006 | MSM | 0 | 0 |
| GCGS0778 | 0.06 | 0.03 | 16 | 0.5 | ORA | 2006 | MSM | 0 | 0 |
| GCGS1015 | 0.032 | 0.016 | 4 | 0.063 | LAX | 2006 | MSW | 0 | 0 |
| GCGS0586 | 0.03 | 0.008 | 0.125 | 8 | HON | 2006 | MSW | 0 | 0 |
| GCGS0635 | 0.03 | 0.015 | 0.008 | 8 | ORA | 2006 | MSM | 0 | 0 |
| GCGS0636 | 0.03 | 0.015 | 4 | 1 | ORA | 2006 | MSW | 0 | 0 |
| GCGS0596 | 0.03 | 0.015 | 4 | 0.5 | LBC | 2006 | - | 0 | 0 |
| GCGS0618 | 0.03 | 0.015 | 0.008 | 0.5 | NYC | 2006 | MSM | 0 | 0 |
| GCGS0634 | 0.03 | 0.03 | 16 | 0.5 | ORA | 2006 | MSW | 0 | 0 |
| GCGS0674 | 0.03 | 0.03 | 32 | 0.5 | POR | 2006 | MSM | 0 | 0 |
| GCGS0435 | 0.015 | 0.008 | 0.004 | 4 | ORA | 2006 | MSM | 0 | 0 |
| GCGS0447 | 0.015 | 0.008 | 0.002 | 4 | PHI | 2006 | MSM | 0 | 0 |
| GCGS0352 | 0.015 | 0.03 | 32 | 2 | DAL | 2006 | MSM | 0 | 0 |
| GCGS0481 | 0.015 | 0.015 | 32 | 2 | POR | 2006 | MSW | 0 | 0 |
| GCGS0368 | 0.015 | 0.008 | 8 | 0.5 | HON | 2006 | MSM | 0 | 0 |
| GCGS0393 | 0.015 | 0.008 | 0.004 | 0.5 | LVG | 2006 | MSM | 0 | 0 |
| GCGS0464 | 0.015 | 0.008 | 8 | 0.5 | PHX | 2006 | MSM | 0 | 0 |
| GCGS0511 | 0.015 | 0.015 | 8 | 0.5 | SFO | 2006 | MSW | 0 | 0 |
| GCGS0285 | 0.008 | 0.008 | 0.004 | 16 | LVG | 2006 | MSM | 0 | 0 |
| GCGS0300 | 0.008 | 0.008 | 0.004 | 8 | SFO | 2006 | MSM | 0 | 0 |
| GCGS0290 | 0.008 | 0.004 | 0.008 | 2 | MIN | 2006 | MSM | 0 | 0 |
| GCGS0292 | 0.008 | 0.004 | 0.004 | 0.125 | OKC | 2006 | MSW | 0 | 0 |
| GCGS0246 | 0.002 | 0.001 | 0.004 | 8 | SEA | 2006 | MSM | 0 | 0 |
| GCGS0243 | 0.002 | 0.001 | 0.004 | 1 | MIN | 2006 | MSM | 0 | 0 |
| GCGS0244 | 0.002 | 0.001 | 0.004 | 1 | MIN | 2006 | MSM | 0 | 0 |
| GCGS1022 | - | 0.015 | 0.008 | 16 | CHI | 2007 | MSM | 0 | 0 |
| GCGS1048 | - | 0.008 | 0.008 | 16 | LVG | 2007 | MSW | 0 | 0 |
| GCGS1082 | - | 0.008 | 0.008 | 16 | POR | 2007 | MSM | 0 | 0 |
| GCGS1083 | - | 0.008 | 0.008 | 16 | POR | 2007 | MSM | 0 | 0 |
| GCGS1025 | - | 0.008 | 0.008 | 8 | CHI | 2007 | MSW | 0 | 0 |
| GCGS1034 | - | 0.008 | 0.008 | 8 | DEN | 2007 | MSW | 0 | 0 |
| GCGS1062 | - | 0.015 | 0.008 | 8 | NYC | 2007 | MSW | 0 | 0 |
| GCGS1018 | - | 0.008 | 16 | 2 | ALB | 2007 | MSM | 0 | 0 |
| GCGS1030 | - | 0.015 | 0.008 | 2 | DEN | 2007 | MSW | 0 | 0 |
| GCGS1031 | - | 0.015 | 0.008 | 2 | DEN | 2007 | MSW | 0 | 0 |
| GCGS1032 | - | 0.008 | 0.008 | 2 | DEN | 2007 | MSW | 0 | 0 |
| GCGS1033 | - | 0.015 | 0.008 | 2 | DEN | 2007 | MSW | 0 | 0 |
| GCGS1043 | - | 0.008 | 16 | 2 | LAX | 2007 | MSM | 0 | 0 |
| GCGS1044 | - | 0.015 | 0.015 | 2 | LAX | 2007 | MSM | 0 | 0 |
| GCGS1045 | - | 0.015 | 0.015 | 2 | LBC | 2007 | MSMW | 0 | 0 |
| GCGS1047 | - | 0.03 | 0.015 | 2 | LVG | 2007 | MSW | 0 | 0 |
| GCGS1054 | - | 0.06 | 16 | 2 | LVG | 2007 | MSM | 0 | 0 |
| GCGS1055 | - | 0.03 | 0.015 | 2 | LVG | 2007 | MSW | 0 | 0 |
| GCGS1087 | - | 0.06 | 4 | 2 | SDG | 2007 | MSM | 0 | 0 |
| GCGS1050 | - | 0.06 | 16 | 1 | LVG | 2007 | MSMW | 0 | 0 |
| GCGS1060 | - | 0.008 | 4 | 1 | MIN | 2007 | MSM | 0 | 0 |
| GCGS1072 | - | 0.03 | 16 | 1 | PHI | 2007 | MSW | 0 | 0 |
| GCGS1076 | - | 0.008 | 0.008 | 1 | PHX | 2007 | MSW | 0 | 0 |
| GCGS1084 | - | 0.03 | 32 | 1 | POR | 2007 | MSMW | 0 | 0 |
| GCGS1086 | - | 0.03 | 8 | 1 | SDG | 2007 | MSM | 0 | 0 |
| GCGS1088 | - | 0.03 | 4 | 1 | SDG | 2007 | MSM | 0 | 0 |
| GCGS1089 | - | 0.06 | 4 | 1 | SDG | 2007 | MSM | 0 | 0 |
| GCGS1090 | - | 0.015 | 16 | 1 | SDG | 2007 | MSM | 0 | 0 |
| GCGS1019 | - | 0.008 | 16 | 0.5 | ALB | 2007 | MSW | 0 | 0 |
| GCGS1020 | - | 0.03 | 16 | 0.5 | ALB | 2007 | MSW | 0 | 0 |
| GCGS1021 | - | 0.03 | 16 | 0.5 | ATL | 2007 | MSM | 0 | 0 |
| GCGS1023 | - | 0.015 | 8 | 0.5 | CHI | 2007 | MSMW | 0 | 0 |
| GCGS1028 | - | 0.008 | 0.008 | 0.5 | CIN | 2007 | MSW | 0 | 0 |
| GCGS1037 | - | 0.03 | 8 | 0.5 | GRB | 2007 | MSMW | 0 | 0 |
| GCGS1038 | - | 0.06 | 4 | 0.5 | KCY | 2007 | MSW | 0 | 0 |
| GCGS1041 | - | 0.03 | 16 | 0.5 | LAX | 2007 | MSMW | 0 | 0 |
| GCGS1046 | - | 0.008 | 0.015 | 0.5 | LBC | 2007 | MSW | 0 | 0 |
| GCGS1053 | - | 0.008 | 0.008 | 0.5 | LVG | 2007 | MSM | 0 | 0 |
| GCGS1059 | - | 0.03 | 16 | 0.5 | MIA | 2007 | MSMW | 0 | 0 |
| GCGS1065 | - | 0.03 | 8 | 0.5 | ORA | 2007 | MSW | 0 | 0 |
| GCGS1066 | - | 0.015 | 4 | 0.5 | ORA | 2007 | MSM | 0 | 0 |
| GCGS1067 | - | 0.03 | 16 | 0.5 | ORA | 2007 | MSM | 0 | 0 |
| GCGS1070 | - | 0.03 | 16 | 0.5 | PHI | 2007 | MSW | 0 | 0 |
| GCGS1071 | - | 0.03 | 8 | 0.5 | PHI | 2007 | MSW | 0 | 0 |
| GCGS1074 | - | 0.03 | 16 | 0.5 | PHX | 2007 | MSM | 0 | 0 |
| GCGS1075 | - | 0.008 | 0.008 | 0.5 | PHX | 2007 | MSW | 0 | 0 |
| GCGS1085 | - | 0.008 | 4 | 0.5 | RIC | 2007 | - | 0 | 0 |
| GCGS1091 | - | 0.008 | 16 | 0.5 | SFO | 2007 | MSM | 0 | 0 |
| GCGS1058 | - | 0.015 | 8 | 0.25 | MIA | 2007 | MSW | 0 | 0 |
| GCGS1092 | - | 0.008 | 2 | 0.125 | SFO | 2007 | MSW | 0 | 0 |
| GCGS1029 | 1 | 1 | 0.125 | 0.25 | CLE | 2007 | MSW | 0 | 0 |
| GCGS1024 | 0.032 | 0.063 | 16 | 0.5 | CHI | 2007 | MSM | 0 | 0 |
| GCGS1035 | 0.032 | 0.125 | 32 | 0.5 | DTR | 2007 | MSW | 0 | 0 |
| GCGS1036 | 0.032 | 0.063 | 16 | 0.5 | DTR | 2007 | MSW | 0 | 0 |
| GCGS1061 | 0.032 | 0.016 | 8 | 0.063 | MIN | 2007 | MSM | 0 | 0 |
| GCGS1026 | - | 0.06 | 16 | 16 | CHI | 2008 | MSM | 0 | 0 |
| GCGS1081 | - | 0.008 | 0.008 | 8 | PHX | 2008 | MSW | 0 | 0 |
| GCGS1039 | - | 0.06 | 0.03 | 4 | KCY | 2008 | MSW | 0 | 0 |
| GCGS1040 | - | 0.03 | 0.015 | 4 | KCY | 2008 | MSW | 0 | 0 |
| GCGS1077 | - | 0.008 | 16 | 2 | PHX | 2008 | MSM | 0 | 0 |
| GCGS1027 | - | 0.03 | 16 | 1 | CHI | 2008 | MSM | 0 | 0 |
| GCGS1069 | - | 0.06 | 16 | 1 | ORA | 2008 | MSM | 0 | 0 |
| GCGS1079 | - | 0.015 | 8 | 1 | PHX | 2008 | MSM | 0 | 0 |
| GCGS1080 | - | 0.03 | 16 | 1 | PHX | 2008 | MSMW | 0 | 0 |
| GCGS1063 | - | 0.008 | 16 | 0.5 | OKC | 2008 | MSMW | 0 | 0 |
| GCGS1064 | - | 0.03 | 16 | 0.5 | OKC | 2008 | MSM | 0 | 0 |
| GCGS1073 | - | 0.03 | 16 | 0.5 | PHI | 2008 | MSW | 0 | 0 |
| GCGS0009 | 0.063 | 0.032 | 0.004 | 0.25 | DEN | 2009 | MSM | 0 | 0 |
| GCGS0013 | 0.063 | 0.032 | 0.004 | 0.25 | DTR | 2009 | MSW | 0 | 0 |
| GCGS0015 | 0.063 | 0.032 | 0.004 | 0.25 | DTR | 2009 | MSW | 0 | 0 |
| GCGS0034 | 0.06 | 0.06 | 32 | 1 | ORA | 2009 | MSW | 0 | 0 |
| GCGS0042 | 0.06 | 0.06 | 8 | 1 | LVG | 2009 | MSM | 0 | 0 |
| GCGS0054 | 0.06 | 0.06 | 16 | 1 | LVG | 2009 | MSW | 0 | 0 |
| GCGS0064 | 0.06 | 0.03 | 0.015 | 1 | POR | 2009 | MSM | 0 | 0 |
| GCGS0014 | 0.06 | 0.06 | 0.015 | 0.5 | DTR | 2009 | MSW | 0 | 0 |
| GCGS0022 | 0.06 | 0.06 | 16 | 0.5 | SDG | 2009 | MSM | 0 | 0 |
| GCGS0024 | 0.06 | 0.06 | 4 | 0.5 | HON | 2009 | MSW | 0 | 1 |
| GCGS0026 | 0.06 | 0.06 | 16 | 0.5 | SDG | 2009 | MSM | 0 | 0 |
| GCGS0044 | 0.06 | 0.06 | 16 | 0.5 | LVG | 2009 | MSW | 0 | 0 |
| GCGS0056 | 0.06 | 0.06 | 8 | 0.5 | PHI | 2009 | MSM | 0 | 0 |
| GCGS0060 | 0.06 | 0.06 | 8 | 0.5 | PHX | 2009 | MSM | 0 | 0 |
| GCGS0132 | 0.06 | 0.03 | 16 | 0.5 | SDG | 2009 | MSM | 0 | 0 |
| GCGS0144 | 0.06 | 0.03 | 0.015 | 0.5 | LVG | 2009 | MSW | 0 | 0 |
| GCGS0238 | 0.06 | 0.03 | 32 | 0.5 | SEA | 2009 | MSM | 0 | 0 |
| GCGS0762 | 0.06 | 0.03 | 16 | 0.5 | MIN | 2009 | MSM | 0 | 0 |
| GCGS0012 | 0.06 | 0.06 | 0.015 | 0.25 | DTR | 2009 | MSW | 0 | 0 |
| GCGS0016 | 0.06 | 0.06 | 0.015 | 0.25 | DTR | 2009 | MSW | 0 | 0 |
| GCGS0082 | 0.06 | 0.06 | 16 | 0.25 | SEA | 2009 | MSM | 0 | 0 |
| GCGS0083 | 0.032 | 0.032 | 0.008 | 0.5 | SFO | 2009 | MSM | 0 | 0 |
| GCGS0202 | 0.03 | 0.008 | 0.015 | 8 | SDG | 2009 | MSM | 0 | 0 |
| GCGS0665 | 0.03 | 0.015 | 0.015 | 4 | PHX | 2009 | MSW | 0 | 0 |
| GCGS0086 | 0.03 | 0.015 | 4 | 1 | SFO | 2009 | MSM | 0 | 0 |
| GCGS0094 | 0.03 | 0.03 | 8 | 1 | CHI | 2009 | MSM | 0 | 0 |
| GCGS0032 | 0.03 | 0.03 | 16 | 0.5 | SDG | 2009 | MSM | 0 | 0 |
| GCGS0036 | 0.03 | 0.015 | 16 | 0.5 | ORA | 2009 | MSW | 0 | 0 |
| GCGS0052 | 0.03 | 0.06 | 8 | 0.5 | LVG | 2009 | MSW | 0 | 0 |
| GCGS0058 | 0.03 | 0.03 | 16 | 0.5 | PHX | 2009 | MSM | 0 | 0 |
| GCGS0070 | 0.03 | 0.015 | 4 | 0.5 | SDG | 2009 | MSW | 0 | 0 |
| GCGS0130 | 0.03 | 0.03 | 16 | 0.5 | SDG | 2009 | MSM | 0 | 0 |
| GCGS0134 | 0.03 | 0.03 | 16 | 0.5 | SDG | 2009 | MSM | 0 | 0 |
| GCGS0148 | 0.03 | 0.015 | 4 | 0.5 | SFO | 2009 | MSW | 0 | 0 |
| GCGS0654 | 0.03 | 0.03 | 16 | 0.5 | PHI | 2009 | MSW | 0 | 0 |
| GCGS0108 | 0.03 | 0.06 | 16 | 0.25 | BAL | 2009 | MSM | 0 | 0 |
| GCGS0146 | 0.03 | 0.008 | 1 | 0.25 | LVG | 2009 | MSMW | 0 | 0 |
| GCGS0158 | 0.03 | 0.03 | 16 | 0.25 | PHI | 2009 | MSM | 0 | 0 |
| GCGS0164 | 0.03 | 0.03 | 16 | 0.25 | PHI | 2009 | MSMW | 0 | 0 |
| GCGS0186 | 0.03 | 0.03 | 2 | 0.25 | LVG | 2009 | MSM | 0 | 0 |
| GCGS0188 | 0.03 | 0.015 | 1 | 0.25 | LVG | 2009 | MSM | 0 | 0 |
| GCGS0210 | 0.03 | 0.015 | 1 | 0.25 | PHX | 2009 | MSM | 0 | 0 |
| GCGS0212 | 0.03 | 0.015 | 1 | 0.25 | SDG | 2009 | MSM | 0 | 0 |
| GCGS0040 | 0.03 | 0.03 | 16 | 0.125 | LVG | 2009 | MSW | 0 | 1 |
| GCGS0198 | 0.015 | 0.008 | 0.015 | 16 | SDG | 2009 | MSM | 0 | 0 |
| GCGS0218 | 0.015 | 0.008 | 0.015 | 16 | SDG | 2009 | MSM | 0 | 0 |
| GCGS0018 | 0.015 | 0.008 | 0.015 | 8 | HON | 2009 | MSM | 0 | 0 |
| GCGS0038 | 0.015 | 0.008 | 0.015 | 8 | LAX | 2009 | MSM | 0 | 0 |
| GCGS0120 | 0.015 | 0.008 | 0.015 | 8 | SDG | 2009 | MSM | 0 | 0 |
| GCGS0224 | 0.015 | 0.008 | 0.015 | 8 | SDG | 2009 | MSM | 0 | 0 |
| GCGS0142 | 0.015 | 0.008 | 0.015 | 2 | LVG | 2009 | MSW | 0 | 0 |
| GCGS0228 | 0.015 | 0.015 | 0.015 | 2 | PHX | 2009 | MSM | 0 | 0 |
| GCGS0330 | 0.015 | 0.015 | 16 | 2 | CHI | 2009 | MSW | 0 | 0 |
| GCGS0002 | 0.015 | 0.03 | 16 | 1 | CHI | 2009 | MSM | 0 | 0 |
| GCGS0004 | 0.015 | 0.03 | 16 | 1 | CHI | 2009 | MSM | 0 | 0 |
| GCGS0006 | 0.015 | 0.03 | 8 | 1 | CHI | 2009 | MSM | 0 | 0 |
| GCGS0028 | 0.015 | 0.008 | 8 | 1 | SDG | 2009 | MSM | 0 | 0 |
| GCGS0030 | 0.015 | 0.008 | 8 | 1 | SDG | 2009 | MSM | 0 | 0 |
| GCGS0066 | 0.015 | 0.008 | 8 | 1 | LVG | 2009 | MSM | 0 | 0 |
| GCGS0080 | 0.015 | 0.06 | 16 | 1 | SEA | 2009 | MSM | 0 | 0 |
| GCGS0084 | 0.015 | 0.015 | 0.015 | 1 | SFO | 2009 | MSM | 0 | 0 |
| GCGS0160 | 0.015 | 0.008 | 8 | 1 | SDG | 2009 | MSM | 0 | 0 |
| GCGS0166 | 0.015 | 0.008 | 8 | 1 | PHX | 2009 | MSM | 0 | 0 |
| GCGS0208 | 0.015 | 0.015 | 8 | 1 | SDG | 2009 | MSM | 0 | 0 |
| GCGS0010 | 0.015 | 0.015 | 0.015 | 0.5 | LVG | 2009 | MSMW | 0 | 0 |
| GCGS0020 | 0.015 | 0.008 | 2 | 0.5 | SDG | 2009 | MSM | 0 | 0 |
| GCGS0068 | 0.015 | 0.008 | 8 | 0.5 | SDG | 2009 | MSMW | 0 | 0 |
| GCGS0072 | 0.015 | 0.008 | 0.015 | 0.5 | SDG | 2009 | MSM | 0 | 0 |
| GCGS0150 | 0.015 | 0.015 | 8 | 0.5 | SDG | 2009 | MSW | 0 | 1 |
| GCGS0154 | 0.015 | 0.008 | 4 | 0.5 | SDG | 2009 | MSW | 0 | 1 |
| GCGS0162 | 0.015 | 0.008 | 0.015 | 0.5 | SDG | 2009 | MSM | 0 | 0 |
| GCGS0182 | 0.015 | 0.008 | 4 | 0.5 | LVG | 2009 | MSM | 0 | 0 |
| GCGS0194 | 0.015 | 0.03 | 4 | 0.5 | ORA | 2009 | MSW | 0 | 0 |
| GCGS0214 | 0.015 | 0.008 | 2 | 0.5 | SDG | 2009 | MSM | 0 | 0 |
| GCGS0222 | 0.015 | 0.03 | 4 | 0.5 | ORA | 2009 | MSW | 0 | 0 |
| GCGS1093 | 0.015 | 0.008 | 0.015 | 0.5 | MIN | 2009 | MSW | 0 | 0 |
| GCGS0008 | 0.015 | 0.008 | 1 | 0.25 | CHI | 2009 | MSMW | 0 | 0 |
| GCGS0062 | 0.015 | 0.015 | 4 | 0.25 | POR | 2009 | MSM | 0 | 0 |
| GCGS0074 | 0.015 | 0.008 | 0.015 | 0.25 | SDG | 2009 | MSM | 0 | 0 |
| GCGS0076 | 0.015 | 0.008 | 8 | 0.25 | SDG | 2009 | MSMW | 0 | 0 |
| GCGS0090 | 0.015 | 0.008 | 1 | 0.25 | SFO | 2009 | MSM | 0 | 0 |
| GCGS0112 | 0.015 | 0.015 | 4 | 0.25 | SDG | 2009 | MSM | 0 | 0 |
| GCGS0180 | 0.015 | 0.015 | 2 | 0.25 | LVG | 2009 | MSM | 0 | 0 |
| GCGS0216 | 0.015 | 0.015 | 2 | 0.25 | SDG | 2009 | MSM | 0 | 1 |
| GCGS0114 | 0.015 | 0.008 | 2 | 0.125 | SDG | 2009 | MSMW | 0 | 0 |
| GCGS0200 | 0.015 | 0.015 | 0.015 | 0.125 | ORA | 2009 | MSW | 0 | 0 |
| GCGS0078 | 0.015 | 0.008 | 4 | 0.06 | SDG | 2009 | MSW | 0 | 0 |
| GCGS0116 | 0.015 | 0.008 | 4 | 0.06 | SDG | 2009 | MSM | 0 | 0 |
| GCGS0206 | 0.015 | 0.008 | 4 | 0.06 | SDG | 2009 | MSM | 0 | 0 |
| GCGS0220 | 0.015 | 0.008 | 2 | 0.06 | SDG | 2009 | MSM | 0 | 0 |
| GCGS0050 | 0.015 | 0.008 | 0.015 | 0.03 | LVG | 2009 | MSW | 0 | 0 |
| GCGS0184 | 0.015 | 0.008 | 2 | 0.03 | LVG | 2009 | MSM | 0 | 1 |
| GCGS0763 | 0.06 | 0.015 | 0.015 | 8 | MIN | 2010 | MSM | 0 | 0 |
| GCGS0140 | 0.06 | 0.06 | 32 | 1 | LAX | 2010 | MSM | 0 | 0 |
| GCGS0168 | 0.06 | 0.06 | 16 | 1 | PHX | 2010 | MSM | 0 | 0 |
| GCGS0725 | 0.06 | 0.03 | 16 | 1 | BAL | 2010 | MSW | 0 | 0 |
| GCGS0152 | 0.06 | 0.06 | 16 | 0.5 | LVG | 2010 | MSW | 0 | 0 |
| GCGS0170 | 0.06 | 0.06 | 16 | 0.5 | PHX | 2010 | MSM | 0 | 0 |
| GCGS0232 | 0.06 | 0.03 | 32 | 0.5 | SEA | 2010 | MSMW | 0 | 0 |
| GCGS0234 | 0.06 | 0.03 | 32 | 0.5 | SEA | 2010 | MSM | 0 | 0 |
| GCGS0236 | 0.06 | 0.03 | 32 | 0.5 | SEA | 2010 | MSM | 0 | 0 |
| GCGS0240 | 0.06 | 0.06 | 32 | 0.5 | SFO | 2010 | MSM | 0 | 0 |
| GCGS0742 | 0.06 | 0.06 | 0.015 | 0.5 | DTR | 2010 | MSW | 0 | 0 |
| GCGS0743 | 0.06 | 0.03 | 0.015 | 0.5 | DTR | 2010 | MSW | 0 | 0 |
| GCGS0744 | 0.06 | 0.06 | 0.015 | 0.5 | DTR | 2010 | - | 0 | 0 |
| GCGS0092 | 0.06 | 0.06 | 0.015 | 0.25 | CHI | 2010 | MSM | 0 | 0 |
| GCGS0100 | 0.06 | 0.06 | 0.015 | 0.25 | DTR | 2010 | MSW | 0 | 0 |
| GCGS0122 | 0.06 | 0.03 | 16 | 0.25 | LAX | 2010 | MSM | 0 | 0 |
| GCGS0126 | 0.06 | 0.03 | 0.015 | 0.25 | LAX | 2010 | MSM | 0 | 0 |
| GCGS0096 | 0.03 | 0.008 | 0.015 | 16 | CHI | 2010 | MSM | 0 | 0 |
| GCGS0098 | 0.03 | 0.008 | 0.015 | 16 | CHI | 2010 | MSM | 0 | 0 |
| GCGS0110 | 0.03 | 0.008 | 0.015 | 16 | SFO | 2010 | MSM | 0 | 0 |
| GCGS0156 | 0.03 | 0.008 | 0.015 | 16 | MIN | 2010 | MSM | 0 | 0 |
| GCGS0174 | 0.03 | 0.008 | 0.015 | 16 | PHX | 2010 | MSM | 0 | 0 |
| GCGS0118 | 0.03 | 0.03 | 0.015 | 8 | HON | 2010 | MSW | 0 | 0 |
| GCGS0128 | 0.03 | 0.008 | 0.015 | 8 | LAX | 2010 | MSM | 0 | 0 |
| GCGS0136 | 0.03 | 0.008 | 0.015 | 8 | LAX | 2010 | MSM | 0 | 0 |
| GCGS0694 | 0.03 | 0.008 | 0.015 | 8 | SDG | 2010 | MSM | 0 | 0 |
| GCGS0638 | 0.03 | 0.015 | 0.015 | 4 | ORA | 2010 | MSW | 0 | 0 |
| GCGS0192 | 0.03 | 0.008 | 0.015 | 2 | SDG | 2010 | MSM | 0 | 0 |
| GCGS0609 | 0.03 | 0.03 | 16 | 2 | MIA | 2010 | MSW | 0 | 0 |
| GCGS0610 | 0.03 | 0.008 | 0.015 | 2 | MIA | 2010 | MSW | 0 | 0 |
| GCGS0178 | 0.03 | 0.015 | 16 | 1 | PHX | 2010 | MSW | 0 | 0 |
| GCGS0190 | 0.03 | 0.03 | 16 | 1 | SDG | 2010 | MSM | 0 | 0 |
| GCGS0196 | 0.03 | 0.03 | 16 | 1 | SDG | 2010 | MSM | 0 | 0 |
| GCGS0557 | 0.03 | 0.03 | 16 | 1 | CHI | 2010 | MSW | 0 | 0 |
| GCGS0637 | 0.03 | 0.03 | 16 | 1 | ORA | 2010 | MSW | 0 | 0 |
| GCGS0591 | 0.03 | 0.03 | 32 | 0.5 | LAX | 2010 | MSM | 0 | 0 |
| GCGS0655 | 0.03 | 0.06 | 0.015 | 0.5 | PHI | 2010 | MSW | 0 | 0 |
| GCGS0692 | 0.03 | 0.06 | 16 | 0.5 | SDG | 2010 | MSM | 0 | 0 |
| GCGS0693 | 0.03 | 0.03 | 8 | 0.5 | SDG | 2010 | MSM | 0 | 0 |
| GCGS0706 | 0.03 | 0.03 | 32 | 0.5 | SEA | 2010 | MSW | 0 | 0 |
| GCGS0707 | 0.03 | 0.03 | 32 | 0.5 | SEA | 2010 | MSW | 0 | 0 |
| GCGS0138 | 0.015 | 0.008 | 0.015 | 16 | LAX | 2010 | MSM | 0 | 0 |
| GCGS0176 | 0.015 | 0.008 | 0.015 | 16 | PHX | 2010 | MSMW | 0 | 0 |
| GCGS0226 | 0.015 | 0.008 | 0.015 | 16 | SDG | 2010 | MSM | 0 | 0 |
| GCGS0106 | 0.015 | 0.008 | 0.015 | 8 | PHX | 2010 | MSW | 0 | 0 |
| GCGS0204 | 0.015 | 0.008 | 0.015 | 8 | SDG | 2010 | MSM | 0 | 0 |
| GCGS0230 | 0.015 | 0.008 | 0.015 | 8 | SDG | 2010 | MSM | 0 | 0 |
| GCGS0172 | 0.015 | 0.008 | 0.015 | 2 | PHX | 2010 | MSM | 0 | 1 |
| GCGS0401 | 0.015 | 0.008 | 0.015 | 2 | MIA | 2010 | - | 0 | 0 |
| GCGS0436 | 0.015 | 0.008 | 0.015 | 2 | ORA | 2010 | MSW | 0 | 0 |
| GCGS0345 | 0.015 | 0.008 | 0.015 | 1 | CLE | 2010 | MSW | 0 | 0 |
| GCGS0382 | 0.015 | 0.008 | 16 | 1 | LAX | 2010 | MSM | 0 | 0 |
| GCGS0344 | 0.015 | 0.008 | 8 | 0.5 | CIN | 2010 | MSM | 0 | 0 |
| GCGS0448 | 0.015 | 0.03 | 16 | 0.5 | PHI | 2010 | MSW | 0 | 0 |
| GCGS0486 | 0.015 | 0.015 | 0.015 | 0.5 | RIC | 2010 | MSW | 0 | 0 |
| GCGS1013 | 0.5 | 0.125 | 0.004 | 0.5 | BAL | 2011 | MSW | 0 | 0 |
| GCGS0857 | 0.125 | 0.015 | 0.015 | 0.5 | SDG | 2011 | MSM | 0 | 0 |
| GCGS0858 | 0.125 | 0.06 | 16 | 0.5 | SDG | 2011 | MSM | 0 | 0 |
| GCGS0832 | 0.125 | 0.063 | 0.002 | 0.25 | DTR | 2011 | MSW | 0 | 0 |
| GCGS0794 | 0.06 | 0.03 | 0.015 | 1 | RIC | 2011 | MSW | 0 | 0 |
| GCGS0730 | 0.06 | 0.03 | 4 | 0.5 | CHI | 2011 | - | 0 | 1 |
| GCGS0754 | 0.06 | 0.03 | 16 | 0.5 | LAX | 2011 | MSM | 0 | 0 |
| GCGS0779 | 0.06 | 0.06 | 16 | 0.5 | ORA | 2011 | MSM | 0 | 0 |
| GCGS0804 | 0.06 | 0.03 | 16 | 0.5 | SDG | 2011 | MSM | 0 | 0 |
| GCGS0805 | 0.06 | 0.03 | 8 | 0.5 | SDG | 2011 | MSM | 0 | 0 |
| GCGS0806 | 0.06 | 0.03 | 16 | 0.5 | SDG | 2011 | MSM | 0 | 0 |
| GCGS0807 | 0.06 | 0.06 | 16 | 0.5 | SDG | 2011 | MSM | 0 | 0 |
| GCGS0809 | 0.06 | 0.03 | 16 | 0.5 | SDG | 2011 | MSM | 0 | 0 |
| GCGS0810 | 0.06 | 0.06 | 32 | 0.5 | SDG | 2011 | MSM | 0 | 0 |
| GCGS0731 | 0.06 | 0.06 | 16 | 0.25 | CHI | 2011 | MSW | 0 | 0 |
| GCGS0764 | 0.06 | 0.03 | 1 | 0.25 | MIN | 2011 | MSM | 0 | 0 |
| GCGS0808 | 0.06 | 0.015 | 8 | 0.25 | SDG | 2011 | MSM | 0 | 0 |
| GCGS0816 | 0.06 | 0.03 | 8 | 0.25 | SFO | 2011 | MSM | 0 | 0 |
| GCGS0817 | 0.06 | 0.03 | 8 | 0.25 | SFO | 2011 | MSM | 0 | 0 |
| GCGS0818 | 0.06 | 0.03 | 8 | 0.25 | SFO | 2011 | MSM | 0 | 0 |
| GCGS0755 | 0.06 | 0.03 | 8 | 0.125 | LAX | 2011 | MSM | 0 | 0 |
| GCGS0768 | 0.06 | 0.03 | 0.015 | 0.125 | NOR | 2011 | MSW | 0 | 0 |
| GCGS0814 | 0.06 | 0.03 | 8 | 0.125 | SFO | 2011 | MSM | 0 | 0 |
| GCGS0815 | 0.06 | 0.03 | 8 | 0.125 | SFO | 2011 | MSM | 0 | 0 |
| GCGS0739 | 0.06 | 0.015 | 2 | 0.06 | DAL | 2011 | MSM | 0 | 0 |
| GCGS0852 | 0.032 | 0.063 | 32 | 2 | PHX | 2011 | MSM | 0 | 0 |
| GCGS0928 | 0.032 | 0.032 | 8 | 0.25 | NYC | 2011 | MSM | 0 | 0 |
| GCGS0708 | 0.03 | 0.015 | 16 | 8 | SEA | 2011 | MSW | 0 | 0 |
| GCGS0696 | 0.03 | 0.015 | 0.015 | 2 | SDG | 2011 | MSM | 0 | 0 |
| GCGS0698 | 0.03 | 0.008 | 0.015 | 2 | SDG | 2011 | MSM | 0 | 0 |
| GCGS0558 | 0.03 | 0.03 | 16 | 1 | CHI | 2011 | MSM | 0 | 0 |
| GCGS0559 | 0.03 | 0.03 | 16 | 1 | CHI | 2011 | MSM | 0 | 0 |
| GCGS0602 | 0.03 | 0.03 | 16 | 0.5 | LVG | 2011 | MSM | 0 | 0 |
| GCGS0656 | 0.03 | 0.03 | 16 | 0.5 | PHI | 2011 | MSW | 0 | 0 |
| GCGS0666 | 0.03 | 0.015 | 4 | 0.5 | PHX | 2011 | MSM | 0 | 0 |
| GCGS0667 | 0.03 | 0.015 | 4 | 0.5 | PHX | 2011 | MSM | 0 | 0 |
| GCGS0676 | 0.03 | 0.03 | 16 | 0.5 | POR | 2011 | MSM | 0 | 0 |
| GCGS0677 | 0.03 | 0.03 | 16 | 0.5 | RIC | 2011 | MSW | 0 | 0 |
| GCGS0695 | 0.03 | 0.015 | 4 | 0.5 | SDG | 2011 | MSM | 0 | 0 |
| GCGS0699 | 0.03 | 0.03 | 16 | 0.5 | SDG | 2011 | MSM | 0 | 0 |
| GCGS0700 | 0.03 | 0.015 | 16 | 0.5 | SDG | 2011 | MSW | 0 | 0 |
| GCGS0543 | 0.03 | 0.008 | 0.015 | 0.25 | BAL | 2011 | MSW | 0 | 0 |
| GCGS0548 | 0.03 | 0.015 | 4 | 0.25 | BHM | 2011 | MSW | 0 | 0 |
| GCGS0549 | 0.03 | 0.015 | 2 | 0.25 | BHM | 2011 | MSW | 0 | 0 |
| GCGS0592 | 0.03 | 0.03 | 16 | 0.25 | LAX | 2011 | MSM | 0 | 0 |
| GCGS0593 | 0.03 | 0.008 | 0.015 | 0.25 | LAX | 2011 | MSM | 0 | 0 |
| GCGS0641 | 0.03 | 0.03 | 4 | 0.25 | ORA | 2011 | MSM | 0 | 0 |
| GCGS0675 | 0.03 | 0.015 | 16 | 0.25 | POR | 2011 | MSM | 0 | 0 |
| GCGS0715 | 0.03 | 0.015 | 8 | 0.25 | SFO | 2011 | MSM | 0 | 0 |
| GCGS0716 | 0.03 | 0.015 | 16 | 0.25 | SFO | 2011 | MSMW | 0 | 0 |
| GCGS0717 | 0.03 | 0.015 | 16 | 0.25 | SFO | 2011 | MSM | 0 | 0 |
| GCGS0594 | 0.03 | 0.015 | 8 | 0.125 | LAX | 2011 | MSM | 0 | 0 |
| GCGS0603 | 0.03 | 0.008 | 4 | 0.125 | LVG | 2011 | MSM | 0 | 1 |
| GCGS0640 | 0.03 | 0.015 | 8 | 0.125 | ORA | 2011 | MSM | 0 | 0 |
| GCGS0697 | 0.03 | 0.015 | 4 | 0.125 | SDG | 2011 | MSW | 0 | 1 |
| GCGS0714 | 0.03 | 0.015 | 4 | 0.125 | SFO | 2011 | MSM | 0 | 0 |
| GCGS0639 | 0.03 | 0.008 | 2 | 0.06 | ORA | 2011 | MSW | 0 | 0 |
| GCGS1017 | 0.016 | 0.016 | 0.004 | 0.25 | PHX | 2011 | MSW | 0 | 0 |
| GCGS0467 | 0.015 | 0.008 | 0.015 | 16 | PHX | 2011 | MSM | 0 | 0 |
| GCGS0332 | 0.015 | 0.008 | 0.015 | 8 | CHI | 2011 | MSM | 0 | 0 |
| GCGS0482 | 0.015 | 0.008 | 0.015 | 8 | POR | 2011 | MSM | 0 | 0 |
| GCGS0484 | 0.015 | 0.008 | 0.015 | 8 | POR | 2011 | MSM | 0 | 0 |
| GCGS0363 | 0.015 | 0.008 | 0.015 | 4 | DTR | 2011 | MSW | 0 | 0 |
| GCGS0470 | 0.015 | 0.008 | 0.015 | 4 | PHX | 2011 | MSM | 0 | 0 |
| GCGS0516 | 0.015 | 0.008 | 0.015 | 4 | SFO | 2011 | MSW | 0 | 0 |
| GCGS0394 | 0.015 | 0.008 | 0.015 | 2 | LVG | 2011 | MSW | 0 | 0 |
| GCGS0468 | 0.015 | 0.008 | 0.015 | 2 | PHX | 2011 | MSW | 0 | 0 |
| GCGS0312 | 0.015 | 0.008 | 0.015 | 0.5 | BAL | 2011 | MSW | 0 | 1 |
| GCGS0360 | 0.015 | 0.015 | 8 | 0.5 | DEN | 2011 | MSW | 0 | 0 |
| GCGS0451 | 0.015 | 0.015 | 16 | 0.5 | PHI | 2011 | MSW | 0 | 0 |
| GCGS0320 | 0.015 | 0.008 | 4 | 0.25 | BHM | 2011 | MSW | 0 | 0 |
| GCGS0321 | 0.015 | 0.008 | 1 | 0.25 | BHM | 2011 | MSW | 0 | 0 |
| GCGS0331 | 0.015 | 0.008 | 4 | 0.25 | CHI | 2011 | MSW | 0 | 1 |
| GCGS0334 | 0.015 | 0.008 | 1 | 0.25 | CHI | 2011 | MSM | 0 | 0 |
| GCGS0375 | 0.015 | 0.008 | 0.015 | 0.25 | KCY | 2011 | MSW | 0 | 0 |
| GCGS0384 | 0.015 | 0.008 | 4 | 0.25 | LAX | 2011 | MSW | 0 | 0 |
| GCGS0428 | 0.015 | 0.008 | 0.015 | 0.25 | OKC | 2011 | MSW | 0 | 0 |
| GCGS0437 | 0.015 | 0.008 | 4 | 0.25 | ORA | 2011 | MSW | 0 | 0 |
| GCGS0450 | 0.015 | 0.008 | 0.015 | 0.25 | PHI | 2011 | MSW | 0 | 0 |
| GCGS0466 | 0.015 | 0.008 | 4 | 0.25 | PHX | 2011 | MSW | 0 | 0 |
| GCGS0469 | 0.015 | 0.008 | 4 | 0.25 | PHX | 2011 | MSM | 0 | 0 |
| GCGS0487 | 0.015 | 0.008 | 2 | 0.25 | RIC | 2011 | MSW | 0 | 0 |
| GCGS0513 | 0.015 | 0.008 | 4 | 0.25 | SFO | 2011 | MSM | 0 | 0 |
| GCGS0319 | 0.015 | 0.008 | 4 | 0.125 | BHM | 2011 | MSW | 0 | 0 |
| GCGS0383 | 0.015 | 0.008 | 2 | 0.125 | LAX | 2011 | MSM | 0 | 1 |
| GCGS0449 | 0.015 | 0.008 | 0.015 | 0.125 | PHI | 2011 | MSW | 0 | 0 |
| GCGS0452 | 0.015 | 0.008 | 8 | 0.125 | PHI | 2011 | MSW | 0 | 0 |
| GCGS0494 | 0.015 | 0.008 | 4 | 0.125 | SDG | 2011 | MSW | 0 | 0 |
| GCGS0333 | 0.015 | 0.008 | 2 | 0.06 | CHI | 2011 | MSW | 0 | 0 |
| GCGS0369 | 0.015 | 0.008 | 2 | 0.06 | HON | 2011 | MSW | 0 | 1 |
| GCGS0370 | 0.015 | 0.008 | 4 | 0.06 | HON | 2011 | MSW | 0 | 1 |
| GCGS0465 | 0.015 | 0.008 | 0.015 | 0.06 | PHX | 2011 | MSW | 0 | 0 |
| GCGS0483 | 0.015 | 0.008 | 4 | 0.06 | POR | 2011 | MSMW | 0 | 0 |
| GCGS0515 | 0.015 | 0.015 | 8 | 0.06 | SFO | 2011 | MSM | 0 | 1 |
| GCGS0517 | 0.015 | 0.008 | 4 | 0.06 | SFO | 2011 | MSM | 0 | 0 |
| GCGS0346 | 0.015 | 0.008 | 2 | 0.03 | CLE | 2011 | MSM | 0 | 0 |
| GCGS0353 | 0.015 | 0.008 | 0.015 | 0.03 | DAL | 2011 | MSW | 0 | 0 |
| GCGS0512 | 0.015 | 0.008 | 8 | 0.03 | SFO | 2011 | MSM | 0 | 0 |
| GCGS0514 | 0.015 | 0.015 | 2 | 0.03 | SFO | 2011 | MSW | 0 | 1 |
| GCGS1095 | 1 | 0.5 | 0.015 | 0.5 | OKC | 2012 | MSW | 0 | 0 |
| GCGS1014 | 0.5 | 0.125 | 0.004 | 0.25 | CHI | 2012 | MSW | 0 | 0 |
| GCGS0933 | 0.125 | 0.063 | 16 | 1 | ORA | 2012 | MSM | 0 | 0 |
| GCGS0604 | 0.063 | 0.032 | 0.016 | 16 | LVG | 2012 | MSW | 0 | 0 |
| GCGS0745 | 0.06 | 0.03 | 0.015 | 256 | HON | 2012 | MSW | 0 | 0 |
| GCGS0732 | 0.06 | 0.008 | 0.015 | 1 | CHI | 2012 | MSM | 0 | 0 |
| GCGS0750 | 0.06 | 0.015 | 0.015 | 0.5 | KCY | 2012 | MSW | 0 | 0 |
| GCGS0787 | 0.06 | 0.008 | 0.015 | 0.5 | PHI | 2012 | MSM | 0 | 0 |
| GCGS0758 | 0.06 | 0.06 | 16 | 0.25 | MIA | 2012 | MSMW | 0 | 0 |
| GCGS0769 | 0.06 | 0.06 | 0.015 | 0.25 | OKC | 2012 | MSW | 0 | 0 |
| GCGS0560 | 0.03 | 0.008 | 0.015 | 16 | CHI | 2012 | MSM | 0 | 0 |
| GCGS0550 | 0.03 | 0.008 | 0.015 | 8 | BHM | 2012 | MSM | 0 | 0 |
| GCGS0574 | 0.03 | 0.015 | 0.03 | 2 | DAL | 2012 | MSM | 0 | 0 |
| GCGS0615 | 0.03 | 0.015 | 0.015 | 2 | MIN | 2012 | MSW | 0 | 0 |
| GCGS0619 | 0.03 | 0.03 | 0.015 | 2 | NYC | 2012 | MSM | 0 | 0 |
| GCGS0671 | 0.03 | 0.03 | 0.015 | 2 | PON | 2012 | MSMW | 0 | 0 |
| GCGS0572 | 0.03 | 0.008 | 0.015 | 1 | COL | 2012 | MSW | 0 | 0 |
| GCGS0658 | 0.03 | 0.015 | 0.015 | 1 | PHI | 2012 | MSM | 0 | 0 |
| GCGS0668 | 0.03 | 0.015 | 0.015 | 1 | PHX | 2012 | MSW | 0 | 0 |
| GCGS0701 | 0.03 | 0.008 | 0.015 | 1 | SDG | 2012 | MSM | 0 | 0 |
| GCGS0544 | 0.03 | 0.015 | 0.015 | 0.5 | BAL | 2012 | MSM | 0 | 0 |
| GCGS0545 | 0.03 | 0.008 | 0.015 | 0.5 | BAL | 2012 | MSW | 0 | 0 |
| GCGS0552 | 0.03 | 0.008 | 0.015 | 0.5 | BHM | 2012 | MSMW | 0 | 0 |
| GCGS0571 | 0.03 | 0.015 | 0.015 | 0.5 | CLE | 2012 | - | 0 | 0 |
| GCGS0614 | 0.03 | 0.03 | 0.015 | 0.5 | MIN | 2012 | MSM | 0 | 0 |
| GCGS0642 | 0.03 | 0.008 | 0.015 | 0.5 | ORA | 2012 | MSW | 0 | 0 |
| GCGS0643 | 0.03 | 0.008 | 0.015 | 0.5 | ORA | 2012 | MSM | 0 | 0 |
| GCGS0657 | 0.03 | 0.015 | 0.015 | 0.5 | PHI | 2012 | - | 0 | 0 |
| GCGS0659 | 0.03 | 0.015 | 16 | 0.5 | PHI | 2012 | MSW | 0 | 0 |
| GCGS0551 | 0.03 | 0.015 | 4 | 0.25 | BHM | 2012 | MSW | 0 | 0 |
| GCGS0679 | 0.03 | 0.008 | 0.015 | 0.25 | RIC | 2012 | MSW | 0 | 0 |
| GCGS0553 | 0.03 | 0.015 | 4 | 0.125 | BHM | 2012 | MSM | 0 | 0 |
| GCGS0678 | 0.03 | 0.015 | 0.015 | 0.125 | RIC | 2012 | - | 0 | 0 |
| GCGS0338 | 0.015 | 0.008 | 0.015 | 16 | CHI | 2012 | MSM | 0 | 0 |
| GCGS0485 | 0.015 | 0.008 | 0.015 | 16 | POR | 2012 | MSM | 0 | 0 |
| GCGS0354 | 0.015 | 0.008 | 0.015 | 8 | DAL | 2012 | MSW | 0 | 0 |
| GCGS0371 | 0.015 | 0.008 | 0.015 | 2 | HON | 2012 | MSMW | 0 | 0 |
| GCGS0473 | 0.015 | 0.008 | 0.015 | 2 | PHX | 2012 | MSMW | 0 | 0 |
| GCGS0523 | 0.015 | 0.008 | 0.015 | 2 | PHX | 2012 | MSM | 0 | 0 |
| GCGS0378 | 0.015 | 0.008 | 0.015 | 1 | KCY | 2012 | MSW | 0 | 0 |
| GCGS0422 | 0.015 | 0.008 | 0.015 | 1 | MIN | 2012 | MSW | 0 | 0 |
| GCGS0472 | 0.015 | 0.008 | 0.015 | 1 | PHX | 2012 | MSW | 0 | 0 |
| GCGS0335 | 0.015 | 0.008 | 0.015 | 0.5 | CHI | 2012 | MSW | 0 | 0 |
| GCGS0337 | 0.015 | 0.008 | 0.015 | 0.5 | CHI | 2012 | MSW | 0 | 0 |
| GCGS0347 | 0.015 | 0.008 | 0.015 | 0.5 | CLE | 2012 | MSW | 0 | 0 |
| GCGS0349 | 0.015 | 0.008 | 0.015 | 0.5 | CLE | 2012 | MSW | 0 | 0 |
| GCGS0376 | 0.015 | 0.008 | 0.5 | 0.5 | KCY | 2012 | MSW | 0 | 0 |
| GCGS0379 | 0.015 | 0.008 | 0.015 | 0.5 | KCY | 2012 | MSW | 0 | 0 |
| GCGS0429 | 0.015 | 0.008 | 0.5 | 0.5 | OKC | 2012 | MSW | 0 | 0 |
| GCGS0455 | 0.015 | 0.008 | 0.015 | 0.5 | PHI | 2012 | MSW | 0 | 0 |
| GCGS0456 | 0.015 | 0.008 | 0.015 | 0.5 | PHI | 2012 | MSW | 0 | 0 |
| GCGS0479 | 0.015 | 0.008 | 0.015 | 0.5 | PON | 2012 | MSW | 0 | 0 |
| GCGS0488 | 0.015 | 0.008 | 0.015 | 0.5 | RIC | 2012 | MSW | 0 | 0 |
| GCGS0496 | 0.015 | 0.008 | 0.015 | 0.5 | SDG | 2012 | MSM | 0 | 0 |
| GCGS0336 | 0.015 | 0.008 | 0.015 | 0.25 | CHI | 2012 | MSW | 0 | 0 |
| GCGS0350 | 0.015 | 0.015 | 0.015 | 0.25 | COL | 2012 | MSW | 0 | 0 |
| GCGS0377 | 0.015 | 0.008 | 0.015 | 0.25 | KCY | 2012 | MSW | 0 | 0 |
| GCGS0395 | 0.015 | 0.008 | 0.015 | 0.25 | LVG | 2012 | MSW | 0 | 0 |
| GCGS0397 | 0.015 | 0.008 | 16 | 0.25 | LVG | 2012 | MSM | 0 | 0 |
| GCGS0399 | 0.015 | 0.008 | 8 | 0.25 | LVG | 2012 | MSMW | 0 | 0 |
| GCGS0430 | 0.015 | 0.008 | 0.015 | 0.25 | OKC | 2012 | MSW | 0 | 0 |
| GCGS0453 | 0.015 | 0.008 | 0.015 | 0.25 | PHI | 2012 | MSW | 0 | 0 |
| GCGS0495 | 0.015 | 0.008 | 16 | 0.25 | SDG | 2012 | MSM | 0 | 0 |
| GCGS0396 | 0.015 | 0.008 | 4 | 0.125 | LVG | 2012 | MSM | 0 | 1 |
| GCGS0454 | 0.015 | 0.008 | 0.015 | 0.125 | PHI | 2012 | - | 0 | 0 |
| GCGS0457 | 0.015 | 0.008 | 0.015 | 0.125 | PHI | 2012 | MSW | 0 | 0 |
| GCGS0471 | 0.015 | 0.008 | 0.015 | 0.125 | PHX | 2012 | MSW | 0 | 0 |
| GCGS0489 | 0.015 | 0.008 | 0.015 | 0.125 | RIC | 2012 | MSW | 0 | 0 |
| GCGS0313 | 0.015 | 0.008 | 0.015 | 0.06 | BAL | 2012 | MSW | 0 | 0 |
| GCGS0348 | 0.015 | 0.008 | 0.015 | 0.06 | CLE | 2012 | MSW | 0 | 0 |
| GCGS0423 | 0.015 | 0.008 | 0.015 | 0.06 | MIN | 2012 | MSW | 0 | 0 |
| GCGS0431 | 0.015 | 0.008 | 0.03 | 0.06 | OKC | 2012 | MSW | 0 | 0 |
| GCGS0398 | 0.015 | 0.008 | 0.5 | 0.03 | LVG | 2012 | MSW | 0 | 0 |
| GCGS0860 | 0.125 | 0.06 | 32 | 8 | PHI | 2013 | MSW | 0 | 0 |
| GCGS0827 | 0.125 | 0.008 | 0.015 | 0.5 | BAL | 2013 | MSW | 0 | 0 |
| GCGS0850 | 0.125 | 0.03 | 1 | 0.5 | PHI | 2013 | MSW | 0 | 0 |
| GCGS0859 | 0.125 | 0.03 | 16 | 0.5 | SDG | 2013 | MSM | 0 | 0 |
| GCGS0792 | 0.06 | 0.03 | 16 | 8 | POR | 2013 | MSM | 0 | 0 |
| GCGS0793 | 0.06 | 0.03 | 16 | 8 | POR | 2013 | MSM | 0 | 0 |
| GCGS0733 | 0.06 | 0.015 | 16 | 2 | CHI | 2013 | MSMW | 0 | 0 |
| GCGS0765 | 0.06 | 0.03 | 0.015 | 2 | MIN | 2013 | MSM | 0 | 0 |
| GCGS0790 | 0.06 | 0.03 | 0.03 | 2 | PON | 2013 | MSMW | 0 | 0 |
| GCGS0791 | 0.06 | 0.06 | 0.03 | 2 | PON | 2013 | MSM | 0 | 0 |
| GCGS0747 | 0.06 | 0.015 | 0.015 | 1 | IND | 2013 | MSM | 0 | 0 |
| GCGS0749 | 0.06 | 0.03 | 0.015 | 1 | IND | 2013 | MSW | 0 | 0 |
| GCGS0770 | 0.06 | 0.03 | 16 | 1 | OKC | 2013 | MSM | 0 | 0 |
| GCGS0746 | 0.06 | 0.06 | 32 | 0.5 | IND | 2013 | MSM | 0 | 0 |
| GCGS0748 | 0.06 | 0.06 | 16 | 0.5 | IND | 2013 | MSM | 0 | 0 |
| GCGS0789 | 0.06 | 0.008 | 0.015 | 0.5 | PHI | 2013 | MSW | 0 | 0 |
| GCGS0788 | 0.06 | 0.03 | 2 | 0.25 | PHI | 2013 | MSM | 0 | 0 |
| GCGS0561 | 0.03 | 0.008 | 0.004 | 2 | CHI | 2013 | MSW | 0 | 0 |
| GCGS0562 | 0.03 | 0.004 | 4 | 2 | CHI | 2013 | MSW | 0 | 0 |
| GCGS0563 | 0.03 | 0.015 | 16 | 2 | CHI | 2013 | MSM | 0 | 0 |
| GCGS0564 | 0.03 | 0.008 | 0.004 | 2 | CHI | 2013 | MSMW | 0 | 0 |
| GCGS0575 | 0.03 | 0.015 | 0.03 | 2 | DAL | 2013 | MSM | 0 | 0 |
| GCGS0587 | 0.03 | 0.015 | 0.015 | 2 | LA2 | 2013 | MSM | 0 | 0 |
| GCGS0611 | 0.03 | 0.015 | 0.015 | 2 | MIA | 2013 | MSM | 0 | 0 |
| GCGS0672 | 0.03 | 0.03 | 0.015 | 2 | PON | 2013 | MSMW | 0 | 0 |
| GCGS0584 | 0.03 | 0.008 | 0.015 | 1 | GRB | 2013 | MSM | 0 | 0 |
| GCGS0616 | 0.03 | 0.015 | 0.015 | 1 | MIN | 2013 | MSW | 0 | 0 |
| GCGS0620 | 0.03 | 0.008 | 0.5 | 1 | OKC | 2013 | MSW | 0 | 0 |
| GCGS0661 | 0.03 | 0.008 | 0.015 | 1 | PHI | 2013 | MSW | 0 | 0 |
| GCGS0669 | 0.03 | 0.008 | 0.015 | 1 | PHX | 2013 | MSW | 0 | 0 |
| GCGS0582 | 0.03 | 0.008 | 0.015 | 0.5 | GRB | 2013 | MSW | 0 | 0 |
| GCGS0583 | 0.03 | 0.008 | 0.015 | 0.5 | GRB | 2013 | MSW | 0 | 0 |
| GCGS0644 | 0.03 | 0.015 | 0.015 | 0.5 | ORA | 2013 | MSW | 0 | 0 |
| GCGS0645 | 0.03 | 0.008 | 0.015 | 0.5 | ORA | 2013 | MSW | 0 | 0 |
| GCGS0660 | 0.03 | 0.008 | 0.015 | 0.5 | PHI | 2013 | MSW | 0 | 0 |
| GCGS0670 | 0.03 | 0.015 | 0.015 | 0.5 | PHX | 2013 | MSM | 0 | 0 |
| GCGS0681 | 0.03 | 0.008 | 0.015 | 0.5 | RIC | 2013 | - | 0 | 0 |
| GCGS0554 | 0.03 | 0.015 | 0.015 | 0.25 | BHM | 2013 | MSW | 0 | 0 |
| GCGS0680 | 0.03 | 0.015 | 0.015 | 0.125 | RIC | 2013 | MSW | 0 | 0 |
| GCGS0438 | 0.015 | 0.008 | 0.015 | 16 | ORA | 2013 | MSW | 0 | 0 |
| GCGS0460 | 0.015 | 0.008 | 0.015 | 16 | PHI | 2013 | MSW | 0 | 1 |
| GCGS0361 | 0.015 | 0.008 | 0.015 | 8 | DEN | 2013 | MSM | 0 | 0 |
| GCGS0372 | 0.015 | 0.008 | 0.015 | 8 | HON | 2013 | MSW | 0 | 0 |
| GCGS0497 | 0.015 | 0.015 | 0.015 | 8 | SDG | 2013 | MSW | 0 | 0 |
| GCGS0518 | 0.015 | 0.008 | 0.015 | 8 | SFO | 2013 | MSW | 0 | 0 |
| GCGS0402 | 0.015 | 0.015 | 0.015 | 4 | MIA | 2013 | MSM | 0 | 0 |
| GCGS0339 | 0.015 | 0.004 | 0.004 | 2 | CHI | 2013 | MSM | 0 | 0 |
| GCGS0355 | 0.015 | 0.015 | 0.03 | 2 | DAL | 2013 | MSW | 0 | 0 |
| GCGS0403 | 0.015 | 0.03 | 0.015 | 2 | MIA | 2013 | MSW | 0 | 0 |
| GCGS0404 | 0.015 | 0.015 | 0.015 | 2 | MIA | 2013 | MSM | 0 | 0 |
| GCGS0405 | 0.015 | 0.015 | 0.015 | 2 | MIA | 2013 | MSM | 0 | 0 |
| GCGS0406 | 0.015 | 0.015 | 0.015 | 2 | MIA | 2013 | MSW | 0 | 0 |
| GCGS0476 | 0.015 | 0.004 | 0.004 | 2 | PHX | 2013 | MSW | 0 | 0 |
| GCGS0477 | 0.015 | 0.008 | 0.004 | 2 | PHX | 2013 | MSM | 0 | 0 |
| GCGS0478 | 0.015 | 0.008 | 0.004 | 2 | PHX | 2013 | MSW | 0 | 0 |
| GCGS0426 | 0.015 | 0.015 | 0.015 | 1 | MIN | 2013 | MSW | 0 | 0 |
| GCGS0433 | 0.015 | 0.008 | 0.015 | 1 | OKC | 2013 | MSW | 0 | 0 |
| GCGS0458 | 0.015 | 0.008 | 0.015 | 1 | PHI | 2013 | MSW | 0 | 0 |
| GCGS0475 | 0.015 | 0.015 | 16 | 1 | PHX | 2013 | MSM | 0 | 0 |
| GCGS0522 | 0.015 | 0.015 | 0.015 | 1 | PHI | 2013 | MSM | 0 | 0 |
| GCGS0316 | 0.015 | 0.008 | 0.015 | 0.5 | BAL | 2013 | MSW | 0 | 0 |
| GCGS0424 | 0.015 | 0.008 | 0.015 | 0.5 | MIN | 2013 | MSW | 0 | 0 |
| GCGS0425 | 0.015 | 0.008 | 0.015 | 0.5 | MIN | 2013 | MSW | 0 | 0 |
| GCGS0427 | 0.015 | 0.008 | 0.015 | 0.5 | MIN | 2013 | MSM | 0 | 0 |
| GCGS0461 | 0.015 | 0.008 | 0.015 | 0.5 | PHI | 2013 | MSW | 0 | 1 |
| GCGS0474 | 0.015 | 0.008 | 0.015 | 0.5 | PHX | 2013 | MSW | 0 | 0 |
| GCGS0459 | 0.015 | 0.008 | 0.015 | 0.25 | PHI | 2013 | - | 0 | 0 |
| GCGS0314 | 0.015 | 0.008 | 2 | 0.125 | BAL | 2013 | MSW | 0 | 0 |
| GCGS0315 | 0.015 | 0.008 | 0.015 | 0.125 | BAL | 2013 | MSW | 0 | 0 |
| GCGS0373 | 0.015 | 0.008 | 0.015 | 0.125 | IND | 2013 | MSW | 0 | 0 |
| GCGS0432 | 0.015 | 0.008 | 0.03 | 0.125 | OKC | 2013 | MSW | 0 | 0 |

**Supplemental Table 2.**

| Identifier | Cefixime MIC (µg/mL) | Ceftriaxone MIC (µg/mL) | Ciprofloxacin MIC (µg/mL) | Azithromycin MIC (µg/mL) | Clinic site | Year | Sex of sex partner | Clade |
| --- | --- | --- | --- | --- | --- | --- | --- | --- |
| GCGS0001 | 0.25 | 0.125 | 16 | 1 | CHI | 2009 | MSM | 1 |
| GCGS0003 | 0.25 | 0.125 | 16 | 1 | CHI | 2009 | MSM | 1 |
| GCGS0005 | 0.5 | 0.06 | 16 | 1 | CHI | 2009 | MSM | 1 |
| GCGS0007 | 0.25 | 0.06 | 16 | 0.5 | CHI | 2009 | MSM | 1 |
| GCGS0017 | 0.25 | 0.06 | 32 | 1 | HON | 2009 | MSM | 1 |
| GCGS0019 | 0.25 | 0.06 | 32 | 1 | HON | 2009 | MSM | 1 |
| GCGS0021 | 0.25 | 0.06 | 32 | 1 | HON | 2009 | MSM | 1 |
| GCGS0023 | 0.25 | 0.03 | 16 | 0.5 | HON | 2009 | MSW | 1 |
| GCGS0025 | 0.25 | 0.06 | 16 | 0.5 | HON | 2009 | MSM | 1 |
| GCGS0027 | 0.25 | 0.03 | 32 | 0.5 | HON | 2009 | MSM | 1 |
| GCGS0029 | 0.25 | 0.06 | 32 | 0.5 | HON | 2009 | MSM | 1 |
| GCGS0031 | 0.5 | 0.03 | 32 | 0.5 | HON | 2009 | MSM | 1 |
| GCGS0033 | 0.25 | 0.06 | 16 | 0.25 | LAX | 2009 | MSW | 1 |
| GCGS0035 | 0.25 | 0.125 | 32 | 1 | LAX | 2009 | MSW | 1 |
| GCGS0037 | 0.5 | 0.125 | 32 | 0.5 | LAX | 2009 | MSM | 1 |
| GCGS0039 | 0.25 | 0.125 | 16 | 1 | LVG | 2009 | MSW | 1 |
| GCGS0041 | 0.25 | 0.125 | 16 | 1 | LVG | 2009 | MSMW | 1 |
| GCGS0043 | 0.25 | 0.06 | 16 | 1 | LVG | 2009 | MSW | 1 |
| GCGS0045 | 0.25 | 0.06 | 16 | 1 | LVG | 2009 | MSW | 1 |
| GCGS0046 | 0.125 | 0.063 | 16 | 1 | LVG | 2009 | MSW | 1 |
| GCGS0047 | 0.25 | 0.06 | 16 | 1 | LVG | 2009 | MSW | 1 |
| GCGS0049 | 0.25 | 0.06 | 16 | 1 | LVG | 2009 | MSW | 1 |
| GCGS0051 | 0.25 | 0.06 | 16 | 0.5 | LVG | 2009 | MSW | 1 |
| GCGS0053 | 0.25 | 0.125 | 16 | 1 | LVG | 2009 | MSW | 1 |
| GCGS0055 | 0.25 | 0.06 | 16 | 1 | PHI | 2009 | MSM | 1 |
| GCGS0057 | 0.25 | 0.06 | 16 | 0.5 | PHX | 2009 | MSM | 1 |
| GCGS0059 | 0.25 | 0.125 | 16 | 1 | PHX | 2009 | MSM | 1 |
| GCGS0061 | 0.5 | 0.06 | 32 | 0.5 | POR | 2009 | MSM | 1 |
| GCGS0063 | 0.25 | 0.06 | 16 | 0.5 | POR | 2009 | MSMW | 1 |
| GCGS0065 | 0.25 | 0.06 | 16 | 0.5 | POR | 2009 | MSM | 1 |
| GCGS0067 | 0.25 | 0.125 | 16 | 0.5 | SDG | 2009 | MSM | 1 |
| GCGS0069 | 0.25 | 0.125 | 16 | 1 | SDG | 2009 | MSW | 1 |
| GCGS0075 | 0.25 | 0.06 | 8 | 0.5 | SDG | 2009 | MSM | 1 |
| GCGS0077 | 0.25 | 0.06 | 16 | 1 | SDG | 2009 | MSW | 1 |
| GCGS0079 | 0.25 | 0.06 | 16 | 0.5 | SEA | 2009 | MSM | 1 |
| GCGS0081 | 0.25 | 0.06 | 16 | 0.125 | SEA | 2009 | MSM | 1 |
| GCGS0085 | 0.25 | 0.03 | 32 | 0.5 | SFO | 2009 | MSM | 1 |
| GCGS0087 | 0.25 | 0.03 | 32 | 0.5 | SFO | 2009 | MSW | 1 |
| GCGS0088 | 0.125 | 0.063 | 16 | 0.5 | SFO | 2009 | MSW | 1 |
| GCGS0089 | 0.25 | 0.03 | 32 | 1 | SFO | 2009 | MSM | 1 |
| GCGS0091 | 0.25 | 0.06 | 16 | 1 | CHI | 2010 | MSM | 1 |
| GCGS0093 | 0.25 | 0.125 | 16 | 1 | CHI | 2010 | MSM | 1 |
| GCGS0095 | 0.25 | 0.06 | 16 | 1 | CHI | 2010 | MSM | 1 |
| GCGS0097 | 0.5 | 0.125 | 16 | 0.5 | CHI | 2010 | MSM | 1 |
| GCGS0101 | 0.25 | 0.06 | 16 | 0.125 | DEN | 2010 | MSM | 1 |
| GCGS0102 | 0.125 | 0.063 | 16 | 16 | ALB | 2010 | MSM | 1 |
| GCGS0103 | 0.25 | 0.03 | 16 | 0.5 | DEN | 2010 | MSM | 1 |
| GCGS0104 | 0.125 | 0.063 | 16 | 4 | ALB | 2010 | MSM | 1 |
| GCGS0105 | 0.25 | 0.06 | 16 | 0.5 | DEN | 2010 | MSW | 1 |
| GCGS0107 | 0.25 | 0.125 | 16 | 0.5 | GRB | 2010 | MSMW | 1 |
| GCGS0109 | 0.25 | 0.06 | 32 | 0.5 | HON | 2010 | MSM | 1 |
| GCGS0111 | 0.25 | 0.25 | 32 | 0.5 | HON | 2010 | MSM | 1 |
| GCGS0113 | 0.25 | 0.03 | 32 | 0.5 | HON | 2010 | MSMW | 1 |
| GCGS0115 | 0.5 | 0.06 | 16 | 0.5 | HON | 2010 | MSM | 1 |
| GCGS0117 | 0.25 | 0.06 | 16 | 0.25 | HON | 2010 | MSW | 1 |
| GCGS0119 | 0.25 | 0.06 | 16 | 0.25 | HON | 2010 | MSMW | 1 |
| GCGS0121 | 0.5 | 0.06 | 16 | 0.5 | LAX | 2010 | MSM | 1 |
| GCGS0123 | 0.25 | 0.125 | 16 | 0.5 | LAX | 2010 | MSMW | 1 |
| GCGS0124 | 0.125 | 0.063 | 16 | 1 | LAX | 2010 | MSM | 1 |
| GCGS0125 | 0.25 | 0.06 | 16 | 0.5 | LAX | 2010 | MSM | 1 |
| GCGS0127 | 0.25 | 0.03 | 8 | 0.25 | LAX | 2010 | MSM | 1 |
| GCGS0129 | 0.25 | 0.03 | 32 | 0.5 | LAX | 2010 | MSM | 1 |
| GCGS0131 | 0.25 | 0.03 | 32 | 0.5 | LAX | 2010 | MSM | 1 |
| GCGS0133 | 0.25 | 0.03 | 32 | 0.5 | LAX | 2010 | MSM | 1 |
| GCGS0135 | 0.5 | 0.03 | 16 | 0.5 | LAX | 2010 | MSM | 1 |
| GCGS0137 | 0.25 | 0.06 | 32 | 0.5 | LAX | 2010 | MSM | 1 |
| GCGS0139 | 0.25 | 0.125 | 32 | 1 | LAX | 2010 | MSM | 1 |
| GCGS0141 | 0.25 | 0.06 | 16 | 0.5 | LVG | 2010 | MSW | 1 |
| GCGS0143 | 0.25 | 0.125 | 16 | 0.5 | LVG | 2010 | MSW | 1 |
| GCGS0145 | 0.25 | 0.06 | 16 | 1 | LVG | 2010 | MSM | 1 |
| GCGS0147 | 0.25 | 0.125 | 16 | 1 | LVG | 2010 | MSW | 1 |
| GCGS0151 | 0.25 | 0.06 | 16 | 1 | LVG | 2010 | MSW | 1 |
| GCGS0155 | 0.25 | 0.125 | 16 | 1 | MIN | 2010 | MSM | 1 |
| GCGS0157 | 0.25 | 0.125 | 16 | 1 | NYC | 2010 | MSM | 1 |
| GCGS0159 | 0.25 | 0.06 | 8 | 0.5 | ORA | 2010 | MSM | 1 |
| GCGS0163 | 0.25 | 0.25 | 16 | 0.5 | PHI | 2010 | MSM | 1 |
| GCGS0165 | 0.25 | 0.06 | 16 | 1 | PHX | 2010 | MSM | 1 |
| GCGS0177 | 0.25 | 0.03 | 4 | 1 | PHX | 2010 | MSW | 1 |
| GCGS0179 | 0.5 | 0.06 | 16 | 0.25 | POR | 2010 | MSM | 1 |
| GCGS0181 | 0.5 | 0.125 | 16 | 0.25 | POR | 2010 | MSM | 1 |
| GCGS0183 | 0.25 | 0.06 | 16 | 0.25 | POR | 2010 | MSM | 1 |
| GCGS0185 | 0.25 | 0.06 | 16 | 0.25 | POR | 2010 | MSM | 1 |
| GCGS0187 | 0.25 | 0.125 | 16 | 0.25 | POR | 2010 | MSM | 1 |
| GCGS0189 | 0.25 | 0.06 | 16 | 1 | SDG | 2010 | MSM | 1 |
| GCGS0195 | 0.25 | 0.06 | 16 | 0.5 | SDG | 2010 | MSMW | 1 |
| GCGS0205 | 0.25 | 0.125 | 16 | 1 | SDG | 2010 | MSM | 1 |
| GCGS0227 | 0.25 | 0.06 | 16 | 1 | SDG | 2010 | MSM | 1 |
| GCGS0229 | 0.25 | 0.06 | 16 | 0.5 | SDG | 2010 | MSM | 1 |
| GCGS0231 | 0.25 | 0.03 | 32 | 0.25 | SEA | 2010 | MSM | 1 |
| GCGS0233 | 0.25 | 0.06 | 32 | 0.5 | SEA | 2010 | MSM | 1 |
| GCGS0235 | 0.25 | 0.06 | 16 | 0.25 | SEA | 2010 | MSM | 1 |
| GCGS0237 | 0.25 | 0.06 | 16 | 0.25 | SEA | 2010 | MSM | 1 |
| GCGS0239 | 0.5 | 0.125 | 16 | 0.5 | SFO | 2010 | MSM | 1 |
| GCGS0241 | 0.5 | 0.125 | 16 | 0.5 | SFO | 2010 | MSM | 1 |
| GCGS0242 | 0.063 | 0.032 | 4 | 0.25 | SFO | 2010 | MSM | 1 |
| GCGS0722 | 0.125 | 0.063 | 16 | 0.5 | ATL | 2012 | MSM | 1 |
| GCGS0740 | 0.125 | 0.032 | 8 | 0.5 | DAL | 2012 | MSW | 1 |
| GCGS0757 | 0.063 | 0.016 | 4 | 1 | MIA | 2011 | MSW | 1 |
| GCGS0759 | 0.125 | 0.063 | 16 | 8 | MIA | 2013 | MSW | 1 |
| GCGS0767 | 0.25 | 0.125 | 16 | 0.5 | NOR | 2011 | MSM | 1 |
| GCGS0780 | 0.125 | 0.032 | 16 | 0.5 | ORA | 2013 | MSM | 1 |
| GCGS0781 | 0.032 | 0.016 | 4 | 0.063 | ORA | 2013 | MSMW | 1 |
| GCGS0811 | 0.125 | 0.063 | 16 | 1 | SDG | 2012 | MSM | 1 |
| GCGS0822 | 0.125 | 0.06 | 8 | 0.5 | ALB | 2012 | MSMW | 1 |
| GCGS0833 | 0.125 | 0.06 | 16 | 0.5 | LAX | 2012 | MSM | 1 |
| GCGS0834 | 0.125 | 0.03 | 4 | 2 | LAX | 2012 | MSM | 1 |
| GCGS0835 | 0.125 | 0.06 | 16 | 0.5 | LVG | 2009 | MSW | 1 |
| GCGS0836 | 0.125 | 0.03 | 16 | 1 | LVG | 2010 | MSW | 1 |
| GCGS0837 | 0.125 | 0.06 | 16 | 1 | LVG | 2012 | MSW | 1 |
| GCGS0838 | 0.125 | 0.03 | 16 | 16 | LVG | 2013 | MSW | 1 |
| GCGS0841 | 0.125 | 0.06 | 16 | 1 | NYC | 2010 | MSM | 1 |
| GCGS0842 | 0.125 | 0.125 | 16 | 2 | NYC | 2011 | MSM | 1 |
| GCGS0844 | 0.125 | 0.06 | 16 | 4 | ORA | 2009 | MSM | 1 |
| GCGS0847 | 0.125 | 0.03 | 8 | 0.5 | PHI | 2009 | MSW | 1 |
| GCGS0848 | 0.125 | 0.06 | 16 | 1 | PHI | 2010 | MSM | 1 |
| GCGS0849 | 0.125 | 0.03 | 16 | 4 | PHI | 2012 | MSM | 1 |
| GCGS0851 | 0.125 | 0.06 | 8 | 0.5 | PHX | 2009 | MSM | 1 |
| GCGS0854 | 0.125 | 0.03 | 16 | 1 | PHX | 2013 | MSW | 1 |
| GCGS0856 | 0.125 | 0.06 | 8 | 8 | POR | 2011 | MSM | 1 |
| GCGS0862 | 0.25 | 0.06 | 8 | 1 | ALB | 2012 | MSM | 1 |
| GCGS0863 | 0.25 | 0.125 | 8 | 1 | ALB | 2012 | MSM | 1 |
| GCGS0864 | 0.25 | 0.125 | 8 | 1 | ALB | 2012 | MSM | 1 |
| GCGS0868 | 0.25 | 0.03 | 8 | 0.5 | BAL | 2013 | MSM | 1 |
| GCGS0869 | 0.25 | 0.03 | 16 | 1 | BAL | 2013 | MSW | 1 |
| GCGS0871 | 0.25 | 0.06 | 16 | 0.25 | BHM | 2011 | MSW | 1 |
| GCGS0872 | 0.25 | 0.06 | 16 | 0.5 | BHM | 2012 | MSW | 1 |
| GCGS0873 | 0.25 | 0.06 | 16 | 1 | CHI | 2011 | MSM | 1 |
| GCGS0874 | 0.25 | 0.125 | 16 | 1 | CHI | 2011 | MSM | 1 |
| GCGS0875 | 0.25 | 0.06 | 16 | 1 | CHI | 2012 | MSW | 1 |
| GCGS0876 | 0.25 | 0.06 | 16 | 1 | CHI | 2012 | MSM | 1 |
| GCGS0877 | 0.25 | 0.06 | 16 | 1 | CHI | 2012 | MSW | 1 |
| GCGS0878 | 0.25 | 0.06 | 16 | 0.5 | CHI | 2012 | MSM | 1 |
| GCGS0879 | 0.25 | 0.06 | 16 | 0.5 | CHI | 2012 | MSM | 1 |
| GCGS0880 | 0.25 | 0.125 | 16 | 0.5 | CHI | 2012 | MSW | 1 |
| GCGS0881 | 0.25 | 0.125 | 16 | 1 | CHI | 2012 | MSM | 1 |
| GCGS0882 | 0.25 | 0.06 | 16 | 1 | CHI | 2013 | MSM | 1 |
| GCGS0883 | 0.25 | 0.03 | 16 | 0.5 | COL | 2012 | MSM | 1 |
| GCGS0884 | 0.25 | 0.06 | 8 | 0.25 | DAL | 2011 | MSW | 1 |
| GCGS0885 | 0.25 | 0.06 | 16 | 0.25 | DAL | 2011 | MSMW | 1 |
| GCGS0886 | 0.25 | 0.125 | 16 | 0.5 | DAL | 2011 | MSM | 1 |
| GCGS0887 | 0.25 | 0.06 | 16 | 1 | DAL | 2012 | MSM | 1 |
| GCGS0888 | 0.25 | 0.06 | 16 | 1 | DEN | 2011 | MSM | 1 |
| GCGS0889 | 0.25 | 0.125 | 16 | 2 | DEN | 2011 | MSW | 1 |
| GCGS0891 | 0.25 | 0.06 | 16 | 0.5 | DEN | 2011 | MSW | 1 |
| GCGS0892 | 0.25 | 0.06 | 16 | 0.5 | DEN | 2011 | MSM | 1 |
| GCGS0893 | 0.25 | 0.06 | 16 | 0.5 | DEN | 2011 | MSM | 1 |
| GCGS0894 | 0.25 | 0.06 | 16 | 0.5 | DEN | 2012 | MSM | 1 |
| GCGS0895 | 0.25 | 0.125 | 16 | 0.5 | HON | 2011 | MSM | 1 |
| GCGS0896 | 0.25 | 0.06 | 16 | 0.25 | HON | 2011 | MSW | 1 |
| GCGS0897 | 0.25 | 0.06 | 16 | 0.25 | HON | 2011 | MSW | 1 |
| GCGS0898 | 0.25 | 0.06 | 16 | 0.25 | HON | 2011 | MSW | 1 |
| GCGS0899 | 0.25 | 0.06 | 16 | 0.5 | HON | 2011 | MSM | 1 |
| GCGS0900 | 0.25 | 0.06 | 16 | 0.25 | HON | 2011 | MSM | 1 |
| GCGS0901 | 0.25 | 0.06 | 8 | 0.5 | HON | 2011 | MSMW | 1 |
| GCGS0902 | 0.25 | 0.06 | 8 | 0.25 | HON | 2011 | MSM | 1 |
| GCGS0903 | 0.25 | 0.06 | 16 | 0.5 | HON | 2012 | MSM | 1 |
| GCGS0904 | 0.25 | 0.06 | 16 | 0.5 | IND | 2013 | MSM | 1 |
| GCGS0905 | 0.25 | 0.06 | 16 | 0.5 | LA2 | 2012 | MSMW | 1 |
| GCGS0906 | 0.25 | 0.06 | 16 | 0.5 | LA2 | 2012 | MSM | 1 |
| GCGS0908 | 0.25 | 0.06 | 16 | 0.25 | LAX | 2011 | MSM | 1 |
| GCGS0909 | 0.25 | 0.06 | 16 | 0.25 | LAX | 2011 | MSM | 1 |
| GCGS0910 | 0.25 | 0.06 | 16 | 0.25 | LAX | 2011 | MSW | 1 |
| GCGS0911 | 0.25 | 0.06 | 16 | 0.25 | LAX | 2011 | MSM | 1 |
| GCGS0912 | 0.25 | 0.125 | 16 | 0.5 | LAX | 2012 | MSMW | 1 |
| GCGS0913 | 0.25 | 0.125 | 16 | 1 | LVG | 2011 | MSM | 1 |
| GCGS0914 | 0.25 | 0.06 | 16 | 1 | LVG | 2012 | MSW | 1 |
| GCGS0915 | 0.25 | 0.125 | 16 | 0.5 | LVG | 2012 | MSM | 1 |
| GCGS0916 | 0.25 | 0.06 | 16 | 1 | LVG | 2013 | MSW | 1 |
| GCGS0917 | 0.25 | 0.06 | 16 | 1 | LVG | 2013 | MSM | 1 |
| GCGS0918 | 0.25 | 0.06 | 16 | 1 | LVG | 2013 | MSM | 1 |
| GCGS0919 | 0.25 | 0.06 | 16 | 0.5 | LVG | 2013 | MSW | 1 |
| GCGS0920 | 0.25 | 0.125 | 8 | 0.5 | MIA | 2005 | MSW | 1 |
| GCGS0921 | 0.25 | 0.06 | 16 | 0.5 | MIA | 2006 | MSMW | 1 |
| GCGS0922 | 0.25 | 0.06 | 8 | 0.5 | MIA | 2012 | MSMW | 1 |
| GCGS0923 | 0.25 | 0.125 | 16 | 0.5 | MIA | 2013 | MSMW | 1 |
| GCGS0924 | 0.25 | 0.06 | 16 | 1 | MIN | 2011 | MSM | 1 |
| GCGS0925 | 0.25 | 0.06 | 16 | 1 | MIN | 2011 | MSM | 1 |
| GCGS0927 | 0.25 | 0.06 | 16 | 1 | NOR | 2012 | MSW | 1 |
| GCGS0929 | 0.25 | 0.06 | 8 | 1 | NYC | 2012 | MSM | 1 |
| GCGS0930 | 0.25 | 0.03 | 16 | 1 | ORA | 2006 | MSW | 1 |
| GCGS0932 | 0.25 | 0.06 | 32 | 0.5 | ORA | 2012 | MSM | 1 |
| GCGS0933 | 0.125 | 0.063 | 16 | 1 | ORA | 2012 | MSM | 1 |
| GCGS0934 | 0.063 | 0.032 | 2 | 0.063 | ORA | 2013 | MSW | 1 |
| GCGS0935 | 0.25 | 0.03 | 8 | 0.5 | ORA | 2013 | MSM | 1 |
| GCGS0936 | 0.25 | 0.06 | 32 | 1 | PHI | 2011 | MSMW | 1 |
| GCGS0937 | 0.25 | 0.125 | 32 | 0.5 | PHI | 2011 | MSM | 1 |
| GCGS0938 | 1 | 0.25 | 16 | 2 | PHI | 2012 | MSM | 1 |
| GCGS0939 | 0.25 | 0.06 | 16 | 0.5 | PHI | 2013 | MSMW | 1 |
| GCGS0940 | 0.25 | 0.03 | 8 | 0.5 | PHI | 2013 | MSM | 1 |
| GCGS0942 | 0.25 | 0.06 | 16 | 1 | PHX | 2012 | MSM | 1 |
| GCGS0943 | 0.25 | 0.06 | 16 | 1 | PON | 2012 | MSMW | 1 |
| GCGS0944 | 0.25 | 0.06 | 16 | 0.5 | POR | 2005 | MSW | 1 |
| GCGS0945 | 0.25 | 0.06 | 16 | 0.25 | POR | 2011 | MSM | 1 |
| GCGS0946 | 0.25 | 0.125 | 16 | 0.25 | POR | 2011 | MSMW | 1 |
| GCGS0947 | 0.25 | 0.06 | 16 | 0.25 | POR | 2011 | MSM | 1 |
| GCGS0948 | 0.25 | 0.06 | 16 | 0.25 | POR | 2011 | MSM | 1 |
| GCGS0949 | 0.25 | 0.06 | 8 | 0.25 | POR | 2011 | MSM | 1 |
| GCGS0950 | 0.25 | 0.125 | 16 | 0.25 | POR | 2011 | MSM | 1 |
| GCGS0951 | 0.25 | 0.06 | 16 | 0.25 | POR | 2011 | MSM | 1 |
| GCGS0952 | 0.25 | 0.06 | 16 | 0.25 | POR | 2011 | MSM | 1 |
| GCGS0953 | 0.25 | 0.06 | 16 | 0.25 | POR | 2011 | MSM | 1 |
| GCGS0954 | 0.25 | 0.06 | 16 | 0.25 | POR | 2011 | MSMW | 1 |
| GCGS0955 | 0.25 | 0.06 | 8 | 0.25 | POR | 2011 | MSM | 1 |
| GCGS0956 | 0.25 | 0.06 | 16 | 0.25 | POR | 2011 | MSM | 1 |
| GCGS0957 | 0.25 | 0.06 | 16 | 0.25 | POR | 2011 | MSM | 1 |
| GCGS0958 | 0.25 | 0.125 | 16 | 0.5 | POR | 2011 | MSM | 1 |
| GCGS0959 | 0.25 | 0.125 | 16 | 0.5 | POR | 2012 | MSM | 1 |
| GCGS0960 | 0.25 | 0.125 | 8 | 0.5 | POR | 2012 | MSMW | 1 |
| GCGS0961 | 0.25 | 0.125 | 16 | 0.5 | POR | 2012 | MSM | 1 |
| GCGS0962 | 0.25 | 0.06 | 16 | 0.5 | POR | 2012 | MSM | 1 |
| GCGS0963 | 0.25 | 0.06 | 16 | 0.5 | POR | 2013 | MSM | 1 |
| GCGS0965 | 0.25 | 0.06 | 16 | 0.5 | SDG | 2011 | MSM | 1 |
| GCGS0966 | 0.25 | 0.125 | 32 | 1 | SDG | 2011 | MSM | 1 |
| GCGS0967 | 0.25 | 0.03 | 16 | 0.5 | SDG | 2011 | MSM | 1 |
| GCGS0968 | 0.125 | 0.032 | 4 | 0.5 | SDG | 2011 | MSW | 1 |
| GCGS0969 | 0.25 | 0.06 | 16 | 0.5 | SDG | 2011 | MSM | 1 |
| GCGS0970 | 0.25 | 0.03 | 16 | 0.5 | SDG | 2011 | MSW | 1 |
| GCGS0971 | 0.25 | 0.06 | 16 | 1 | SDG | 2011 | MSM | 1 |
| GCGS0972 | 0.25 | 0.06 | 16 | 1 | SDG | 2011 | MSM | 1 |
| GCGS0973 | 0.25 | 0.06 | 8 | 0.5 | SDG | 2011 | MSM | 1 |
| GCGS0974 | 0.25 | 0.06 | 16 | 1 | SDG | 2011 | MSMW | 1 |
| GCGS0975 | 0.25 | 0.06 | 32 | 0.5 | SDG | 2011 | MSM | 1 |
| GCGS0976 | 0.25 | 0.06 | 32 | 0.25 | SDG | 2011 | MSW | 1 |
| GCGS0977 | 0.25 | 0.06 | 16 | 0.5 | SDG | 2012 | MSM | 1 |
| GCGS0978 | 0.25 | 0.06 | 16 | 0.5 | SDG | 2012 | MSM | 1 |
| GCGS0979 | 0.25 | 0.06 | 16 | 0.5 | SDG | 2012 | MSM | 1 |
| GCGS0980 | 0.25 | 0.06 | 16 | 0.5 | SDG | 2012 | MSM | 1 |
| GCGS0981 | 0.25 | 0.06 | 16 | 0.5 | SDG | 2012 | MSW | 1 |
| GCGS0982 | 0.25 | 0.06 | 16 | 1 | SDG | 2012 | MSW | 1 |
| GCGS0983 | 0.25 | 0.06 | 16 | 1 | SDG | 2012 | MSM | 1 |
| GCGS0984 | 0.25 | 0.06 | 16 | 0.25 | SDG | 2012 | MSM | 1 |
| GCGS0985 | 0.25 | 0.06 | 16 | 1 | SDG | 2012 | MSM | 1 |
| GCGS0986 | 0.25 | 0.06 | 16 | 0.25 | SDG | 2012 | MSM | 1 |
| GCGS0987 | 0.25 | 0.06 | 16 | 0.5 | SDG | 2013 | MSM | 1 |
| GCGS0988 | 0.25 | 0.03 | 8 | 0.5 | SDG | 2013 | MSW | 1 |
| GCGS0989 | 0.25 | 0.03 | 16 | 0.5 | SDG | 2013 | MSW | 1 |
| GCGS0990 | 0.125 | 0.032 | 16 | 0.5 | SDG | 2013 | MSM | 1 |
| GCGS0991 | 0.125 | 0.032 | 16 | 0.5 | SDG | 2013 | MSW | 1 |
| GCGS0992 | 0.25 | 0.06 | 16 | 0.5 | SEA | 2011 | MSM | 1 |
| GCGS0993 | 0.25 | 0.06 | 16 | 0.25 | SEA | 2011 | MSM | 1 |
| GCGS0994 | 0.25 | 0.06 | 16 | 0.25 | SEA | 2011 | MSW | 1 |
| GCGS0995 | 0.25 | 0.125 | 16 | 0.25 | SEA | 2011 | MSM | 1 |
| GCGS0996 | 0.25 | 0.06 | 16 | 0.5 | SEA | 2012 | MSW | 1 |
| GCGS0997 | 0.25 | 0.06 | 16 | 0.5 | SEA | 2012 | MSM | 1 |
| GCGS0998 | 0.25 | 0.125 | 16 | 0.5 | SEA | 2012 | MSM | 1 |
| GCGS0999 | 0.25 | 0.06 | 16 | 0.5 | SFO | 2011 | MSM | 1 |
| GCGS1000 | 0.25 | 0.06 | 16 | 0.25 | SFO | 2011 | MSM | 1 |
| GCGS1001 | 0.25 | 0.125 | 32 | 0.5 | SFO | 2011 | MSW | 1 |
| GCGS1002 | 0.25 | 0.06 | 8 | 0.25 | SFO | 2011 | MSM | 1 |
| GCGS1003 | 0.25 | 0.06 | 16 | 0.25 | SFO | 2011 | MSW | 1 |
| GCGS1004 | 0.25 | 0.125 | 16 | 0.25 | SFO | 2011 | MSM | 1 |
| GCGS1005 | 0.25 | 0.06 | 16 | 0.5 | SFO | 2011 | MSM | 1 |
| GCGS1006 | 0.25 | 0.06 | 16 | 1 | SFO | 2012 | MSM | 1 |
| GCGS1007 | 0.25 | 0.125 | 16 | 1 | SFO | 2012 | MSM | 1 |
| GCGS1008 | 0.25 | 0.06 | 16 | 0.5 | SFO | 2013 | MSM | 1 |
| GCGS1009 | 0.25 | 0.125 | 16 | 0.5 | SFO | 2013 | MSM | 1 |
| GCGS1010 | 0.25 | 0.06 | 16 | 0.5 | SFO | 2013 | MSM | 1 |
| GCGS1011 | 0.25 | 0.06 | 16 | 0.5 | SFO | 2013 | MSW | 1 |
| GCGS1012 | 0.25 | 0.06 | 16 | 1 | PHX | 2011 | MSMW | 1 |
| GCGS1016 | 0.5 | 0.125 | 16 | 1 | MIN | 2011 | MSM | 1 |
| GCGS1042 | 0.25 | 0.125 | 16 | 1 | LAX | 2007 | MSW | 1 |
| GCGS1049 | 0.125 | 0.125 | 16 | 1 | LVG | 2007 | MSW | 1 |
| GCGS1051 | 0.125 | 0.063 | 16 | 1 | LVG | 2007 | MSW | 1 |
| GCGS1052 | 0.125 | 0.063 | 16 | 1 | LVG | 2007 | MSW | 1 |
| GCGS1056 | 0.125 | 0.063 | 16 | 1 | LVG | 2007 | MSW | 1 |
| GCGS1057 | 1 | 0.25 | 6 | 0.5 | LVG | 2008 | MSW | 1 |
| GCGS1068 | 0.125 | 0.063 | 16 | 0.5 | ORA | 2008 | MSM | 1 |
| GCGS1078 | 0.25 | 0.125 | 16 | 0.5 | PHX | 2008 | MSW | 1 |
| GCGS0071 | 0.25 | 0.03 | 0.015 | 0.5 | SDG | 2009 | MSM | 2 |
| GCGS0073 | 0.25 | 0.06 | 0.015 | 0.25 | SDG | 2009 | MSMW | 2 |
| GCGS0149 | 0.25 | 0.03 | 0.015 | 0.5 | LVG | 2010 | MSW | 2 |
| GCGS0153 | 0.25 | 0.03 | 0.015 | 0.5 | LVG | 2010 | MSW | 2 |
| GCGS0161 | 0.25 | 0.03 | 0.015 | 0.5 | ORA | 2010 | MSM | 2 |
| GCGS0167 | 0.25 | 0.03 | 0.015 | 0.5 | PHX | 2010 | MSM | 2 |
| GCGS0169 | 0.25 | 0.03 | 0.015 | 0.5 | PHX | 2010 | MSM | 2 |
| GCGS0171 | 0.25 | 0.06 | 0.015 | 0.5 | PHX | 2010 | MSM | 2 |
| GCGS0173 | 0.25 | 0.06 | 0.015 | 1 | PHX | 2010 | MSM | 2 |
| GCGS0175 | 0.25 | 0.06 | 0.015 | 0.5 | PHX | 2010 | MSMW | 2 |
| GCGS0191 | 0.25 | 0.03 | 0.015 | 0.5 | SDG | 2010 | MSM | 2 |
| GCGS0193 | 0.25 | 0.03 | 0.015 | 0.5 | SDG | 2010 | MSW | 2 |
| GCGS0197 | 0.25 | 0.032 | 0.004 | 0.5 | SDG | 2010 | MSM | 2 |
| GCGS0199 | 0.25 | 0.063 | 0.004 | 0.5 | SDG | 2010 | MSW | 2 |
| GCGS0201 | 0.25 | 0.063 | 0.004 | 0.5 | SDG | 2010 | MSM | 2 |
| GCGS0203 | 0.25 | 0.06 | 0.015 | 0.5 | SDG | 2010 | MSM | 2 |
| GCGS0207 | 0.25 | 0.06 | 0.015 | 0.5 | SDG | 2010 | MSM | 2 |
| GCGS0209 | 0.25 | 0.03 | 0.015 | 0.5 | SDG | 2010 | MSM | 2 |
| GCGS0211 | 0.25 | 0.03 | 0.015 | 0.25 | SDG | 2010 | MSM | 2 |
| GCGS0213 | 0.5 | 0.063 | 0.004 | 0.5 | SDG | 2010 | MSM | 2 |
| GCGS0215 | 0.25 | 0.03 | 0.015 | 0.25 | SDG | 2010 | MSM | 2 |
| GCGS0217 | 0.25 | 0.03 | 0.015 | 0.5 | SDG | 2010 | MSM | 2 |
| GCGS0219 | 0.25 | 0.03 | 0.015 | 0.5 | SDG | 2010 | MSM | 2 |
| GCGS0221 | 0.25 | 0.03 | 0.015 | 0.5 | SDG | 2010 | MSW | 2 |
| GCGS0223 | 0.25 | 0.03 | 0.015 | 0.5 | SDG | 2010 | MSM | 2 |
| GCGS0225 | 0.25 | 0.03 | 0.015 | 0.5 | SDG | 2010 | MSM | 2 |
| GCGS0820 | 0.125 | 0.015 | 0.015 | 0.5 | ALB | 2010 | MSM | 2 |
| GCGS0821 | 0.125 | 0.03 | 0.015 | 0.5 | ALB | 2011 | MSM | 2 |
| GCGS0853 | 0.125 | 0.06 | 0.015 | 0.5 | PHX | 2011 | MSM | 2 |
| GCGS0890 | 0.25 | 0.06 | 0.015 | 0.25 | DEN | 2011 | MSM | 2 |
| GCGS0931 | 0.25 | 0.03 | 0.015 | 0.5 | ORA | 2011 | MSMW | 2 |
| GCGS0941 | 0.25 | 0.03 | 0.015 | 0.5 | PHX | 2011 | MSM | 2 |
| GCGS0964 | 0.25 | 0.03 | 0.015 | 0.25 | SDG | 2011 | MSM | 2 |

**Supplemental Table 3.**

| **mtrA** |  |  |  |  |
| --- | --- | --- | --- | --- |
|  | **Alternative start** | Present | Absent |  |
|  | Reduced Susceptibility | 4 | 290 |  |
|  | Susceptible | 10 | 798 |  |
|  |  |  |  |  |
|  | **AA Position 36** | C | R |  |
|  | Reduced Susceptibility | 285 | 9 |  |
|  | Susceptible | 785 | 23 |  |
|  |  |  |  |  |
|  | **AA Position 178** | P | L |  |
|  | Reduced Susceptibility | 8 | 286 |  |
|  | Susceptible | 12 | 796 |  |
|  |  |  |  |  |
| **norM** |  |  |  |  |
|  | **AA Position 295** | M | I |  |
|  | Reduced Susceptibility | 291 | 3 |  |
|  | Susceptible | 788 | 20 |  |
|  |  |  |  |  |
|  | **AA Position 357** | S | P |  |
|  | Reduced Susceptibility | 2 | 292 |  |
|  | Susceptible | 36 | 772 |  |
|  |  |  |  |  |
|  | **AA Position 457** | K | E |  |
|  | Reduced Susceptibility | 237 | 57 |  |
|  | Susceptible | 307 | 501 |  |
|  |  |  |  |  |
| **macA** |  |  |  |  |
|  | **AA Position 22** | H | S |  |
|  | Reduced Susceptibility | 275 | 19 |  |
|  | Susceptible | 767 | 41 |  |
|  |  |  |  |  |
|  | **AA Position 31** | S | A |  |
|  | Reduced Susceptibility | 203 | 91 |  |
|  | Susceptible | 697 | 111 |  |
|  |  |  |  |  |
|  | **AA Position 97** | I | L |  |
|  | Reduced Susceptibility | 9 | 285 |  |
|  | Susceptible | 21 | 787 |  |
|  |  |  |  |  |
|  | **AA Position 98** | N | D |  |
|  | Reduced Susceptibility | 66 | 228 |  |
|  | Susceptible | 65 | 743 |  |
|  |  |  |  |  |
|  | **AA Position 129** | A | T |  |
|  | Reduced Susceptibility | 223 | 71 |  |
|  | Susceptible | 746 | 62 |  |
|  |  |  |  |  |
|  | **AA Position 192** | S | A |  |
|  | Reduced Susceptibility | 3 | 291 |  |
|  | Susceptible | 13 | 795 |  |
|  |  |  |  |  |
|  | **AA Position 207** | A | T |  |
|  | Reduced Susceptibility | 5 | 289 |  |
|  | Susceptible | 14 | 794 |  |
|  |  |  |  |  |
|  | **AA Position 341** | A | V |  |
|  | Reduced Susceptibility | 180 | 114 |  |
|  | Susceptible | 539 | 269 |  |
|  |  |  |  |  |
|  | **AA Position 384** | A | V |  |
|  | Reduced Susceptibility | 232 | 62 |  |
|  | Susceptible | 623 | 185 |  |
|  |  |  |  |  |
|  | **AA Position 385** | M | L |  |
|  | Reduced Susceptibility | 62 | 232 |  |
|  | Susceptible | 185 | 623 |  |
|  |  |  |  |  |
| **macB** |  |  |  |  |
|  | **AA Position 12** | C | Y |  |
|  | Reduced Susceptibility | 286 | 8 |  |
|  | Susceptible | 792 | 16 |  |
|  |  |  |  |  |
|  | **AA Position 239** | Q | R |  |
|  | Reduced Susceptibility | 262 | 32 |  |
|  | Susceptible | 760 | 48 |  |
|  |  |  |  |  |
|  | **AA Position 577** | - | I | V |
|  | Reduced Susceptibility | 0 | 291 | 3 |
|  | Susceptible | 7 | 776 | 25 |

**Supplemental Table 4.**

| mtr mosaic cluster / isolate number | Identifier | Azithromycin MIC (µg/mL) | Clinic | Year |
| --- | --- | --- | --- | --- |
| 1 | GCGS0275 | 2 | KCY | 2000 |
| 1 | GCGS0276 | 1 | KCY | 2000 |
| 1 | GCGS0249 | 4 | KCY | 2000 |
| 1 | GCGS0374 | 1 | KCY | 2000 |
| 2 | GCGS0138 | 16 | LAX | 2010 |
| 2 | GCGS0128 | 8 | LAX | 2010 |
| 2 | GCGS0136 | 8 | LAX | 2010 |
| 2 | GCGS0038 | 8 | LAX | 2009 |
| 3 | GCGS0402 | 4 | MIA | 2013 |
| 3 | GCGS0403 | 2 | MIA | 2013 |
| 3 | GCGS0611 | 2 | MIA | 2013 |
| 3 | GCGS0405 | 2 | MIA | 2013 |
| 3 | GCGS0404 | 2 | MIA | 2013 |
| 3 | GCGS0406 | 2 | MIA | 2013 |
| 3 | GCGS0587 | 2 | LA2 | 2013 |
| 3 | GCGS0497 | 8 | SDG | 2013 |
| 3 | GCGS0355 | 2 | DAL | 2013 |
| 3 | GCGS0522 | 1 | PHI | 2013 |
| 3 | GCGS0658 | 1 | PHI | 2012 |
| 3 | GCGS0574 | 2 | DAL | 2012 |
| 3 | GCGS0575 | 2 | DAL | 2013 |
| 4 | GCGS0296 | 2 | SDG | 2005 |
| 4 | GCGS0298 | 2 | SDG | 2005 |
| 4 | GCGS0297 | 2 | SDG | 2005 |
| 5 | GCGS0273 | 2 | FBG | 2000 |
| 5 | GCGS0525 | 2 | ATL | 2000 |
| 6 | GCGS0481 | 2 | POR | 2006 |
| 7 | GCGS0834 | 2 | LAX | 2012 |
